# Supplementary figures and images for: LC3-associated phagocytosis of neutrophils triggers tumor ferroptotic cell death in glioblastoma (part 1 of 2)
Source: EMBO J. 2024 May 28;43(13):4. doi: 10.1038/s44318-024-00130-4 (PMC11217441; doi:10.1038/s44318-024-00130-4)

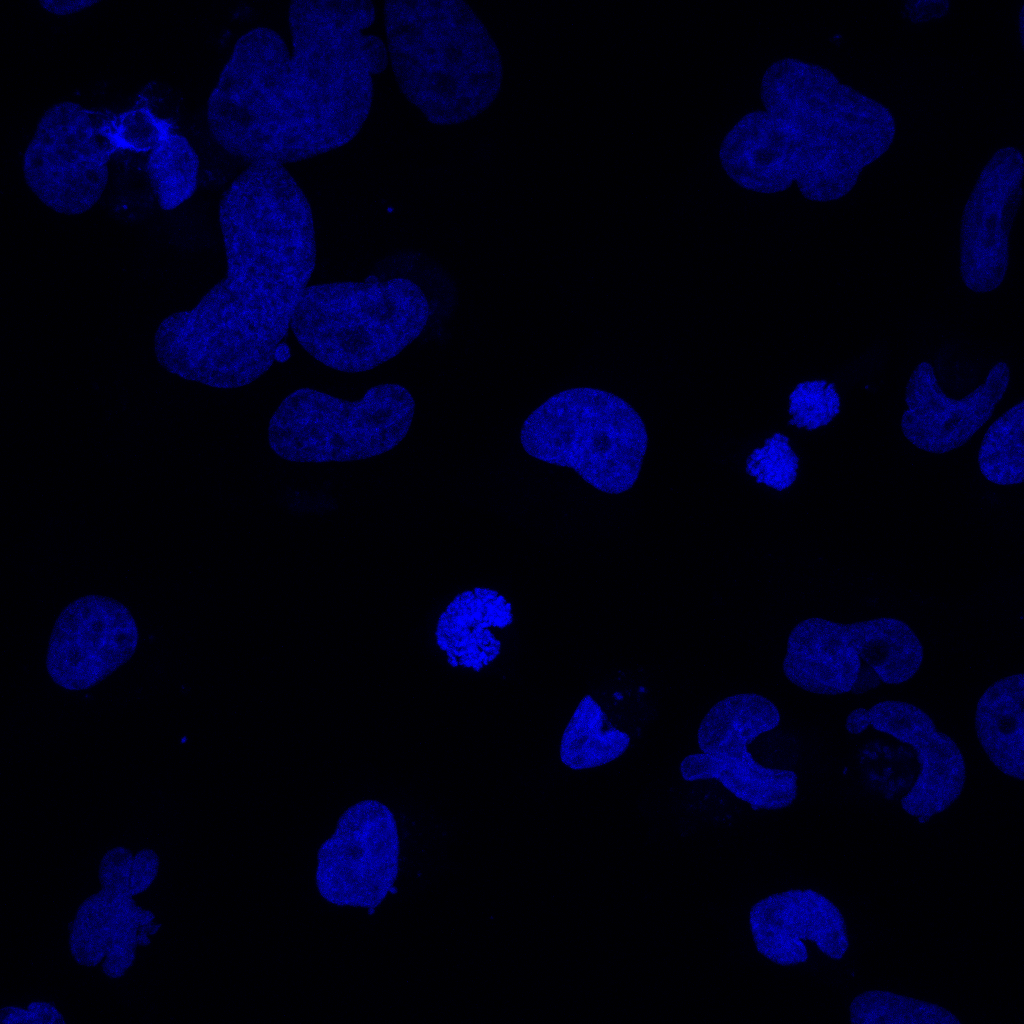

Supplement: Supplementary file 2 — Source data Fig. 1 [file 44318_2024_130_MOESM2_ESM.zip › Figure 1/1J/DMSO/DAPI.tif]

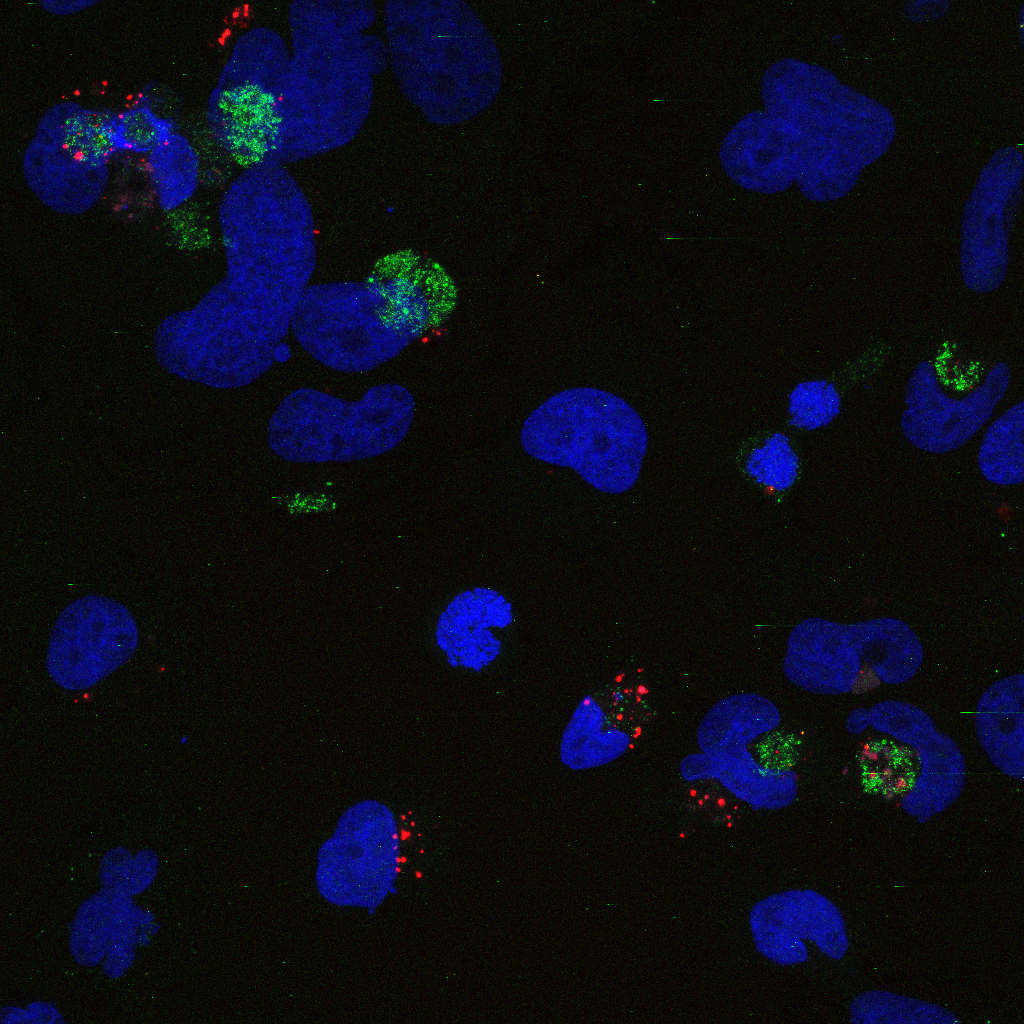

Supplement: Supplementary file 2 — Source data Fig. 1 [file 44318_2024_130_MOESM2_ESM.zip › Figure 1/1J/DMSO/Merged.tif]

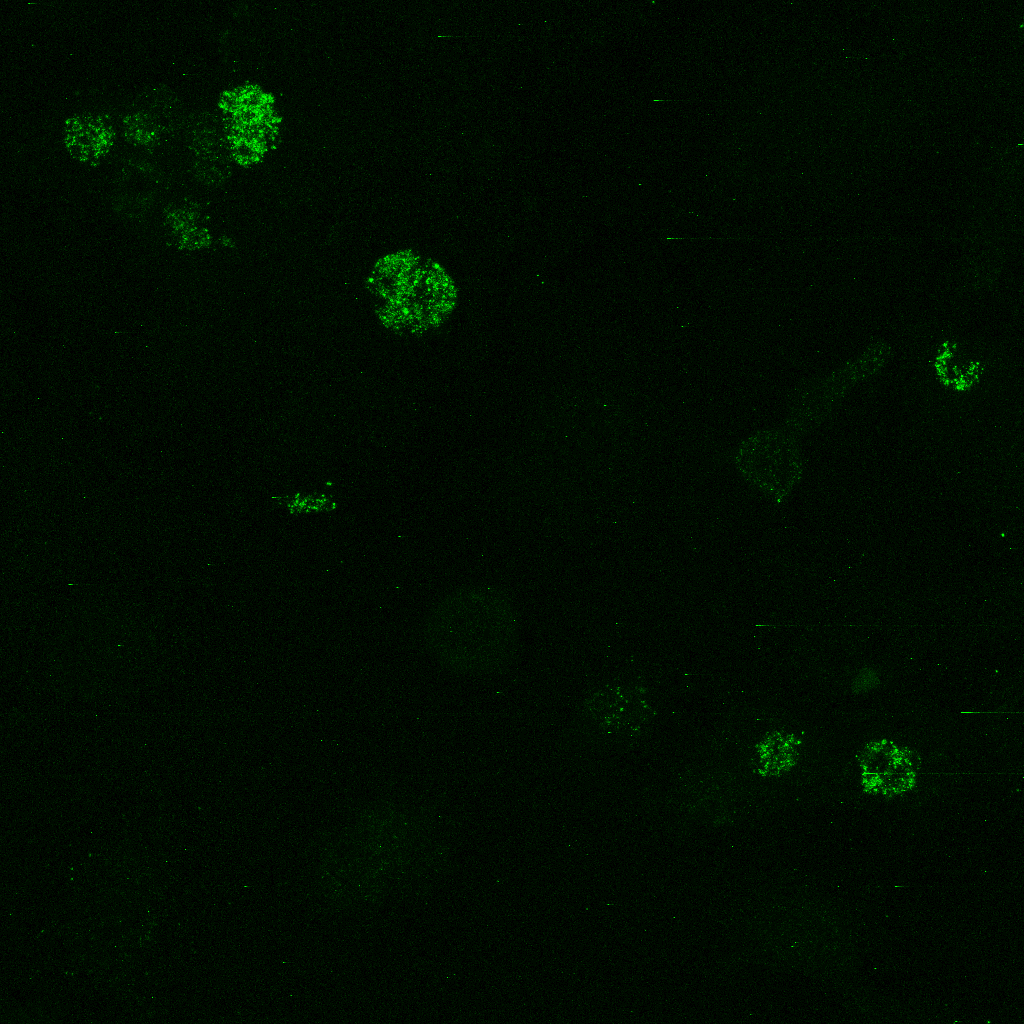

Supplement: Supplementary file 2 — Source data Fig. 1 [file 44318_2024_130_MOESM2_ESM.zip › Figure 1/1J/DMSO/MPO.tif]

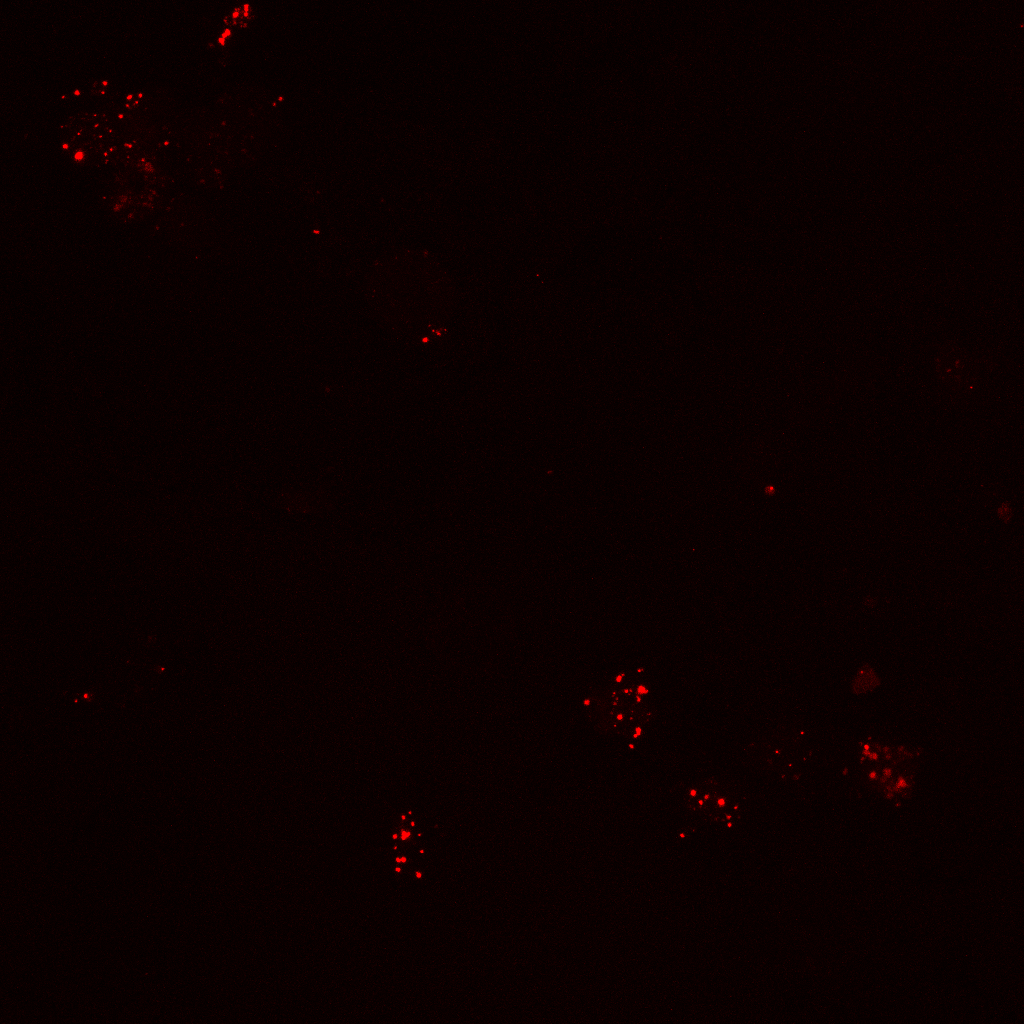

Supplement: Supplementary file 2 — Source data Fig. 1 [file 44318_2024_130_MOESM2_ESM.zip › Figure 1/1J/DMSO/PKH26.tif]

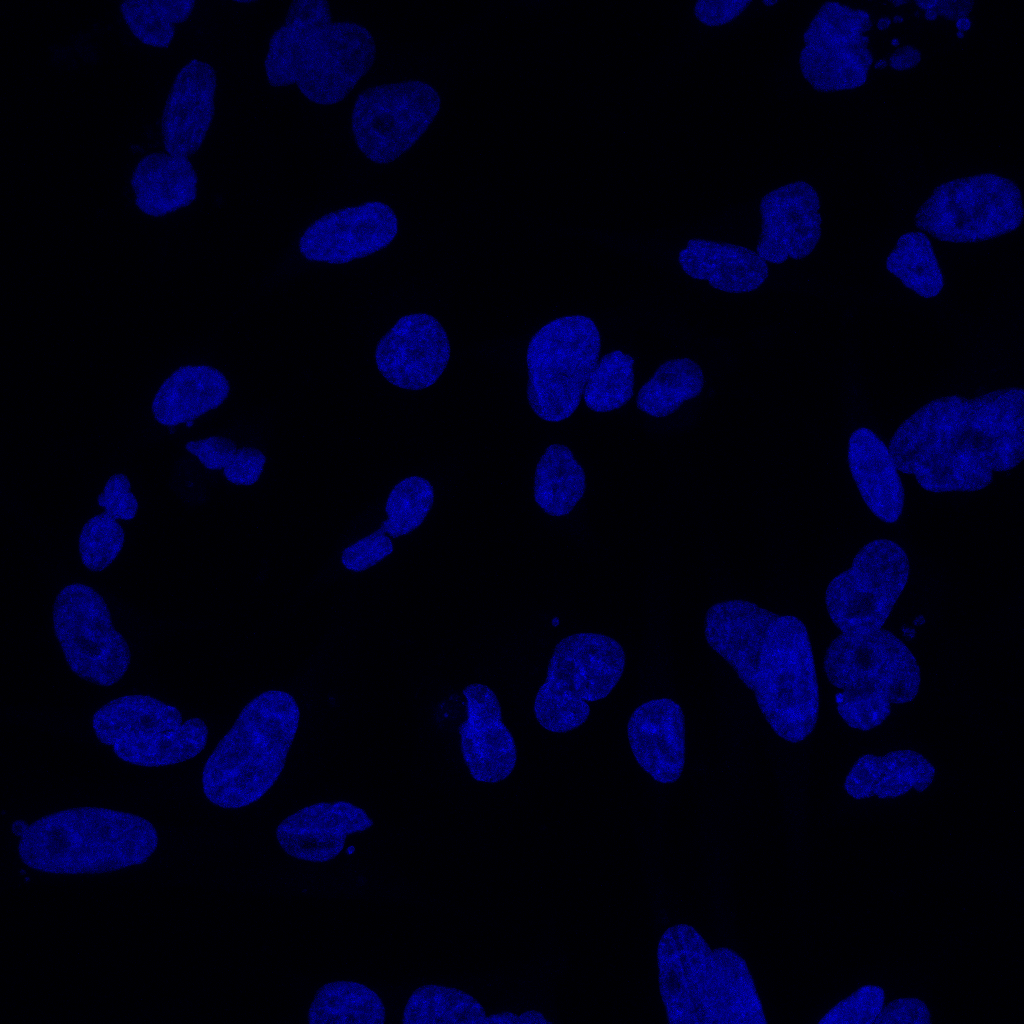

Supplement: Supplementary file 2 — Source data Fig. 1 [file 44318_2024_130_MOESM2_ESM.zip › Figure 1/1J/Fluvastatin/DAPI.tif]

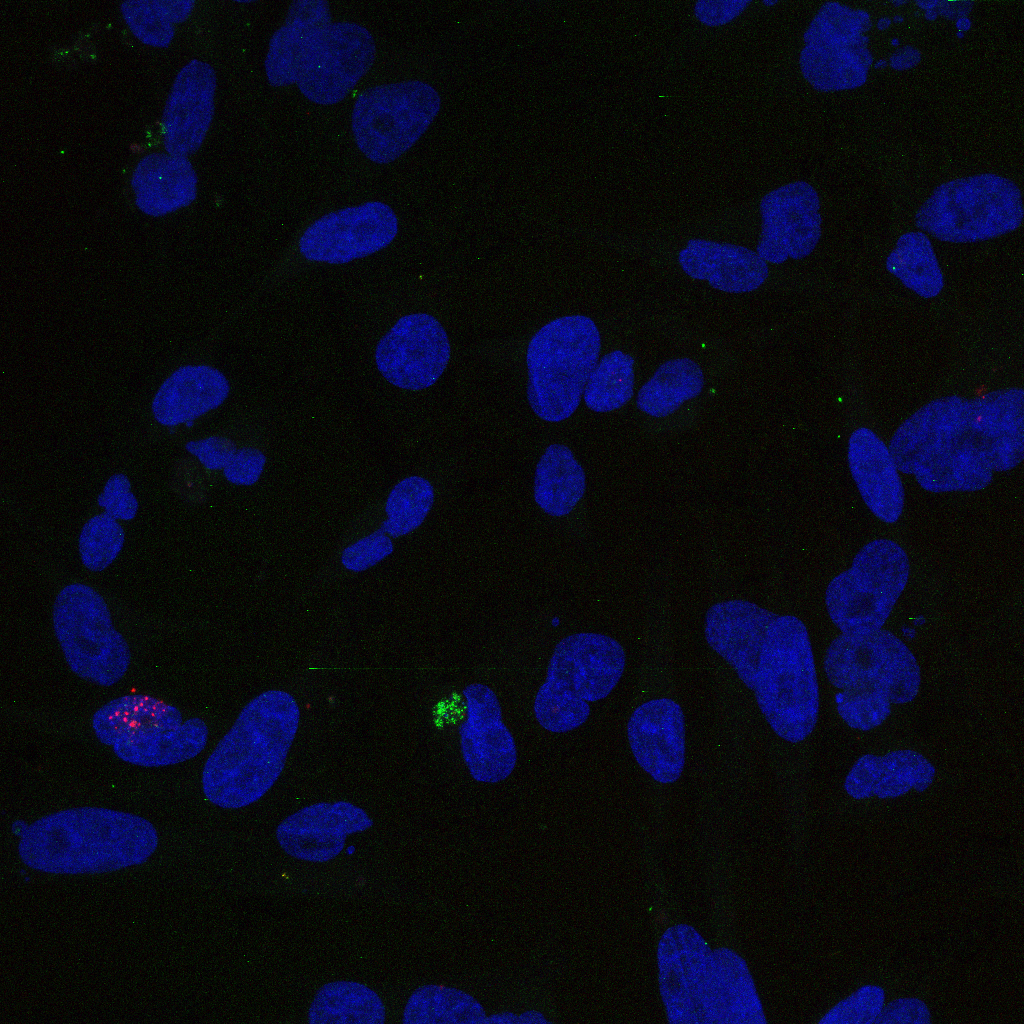

Supplement: Supplementary file 2 — Source data Fig. 1 [file 44318_2024_130_MOESM2_ESM.zip › Figure 1/1J/Fluvastatin/Merged.tif]

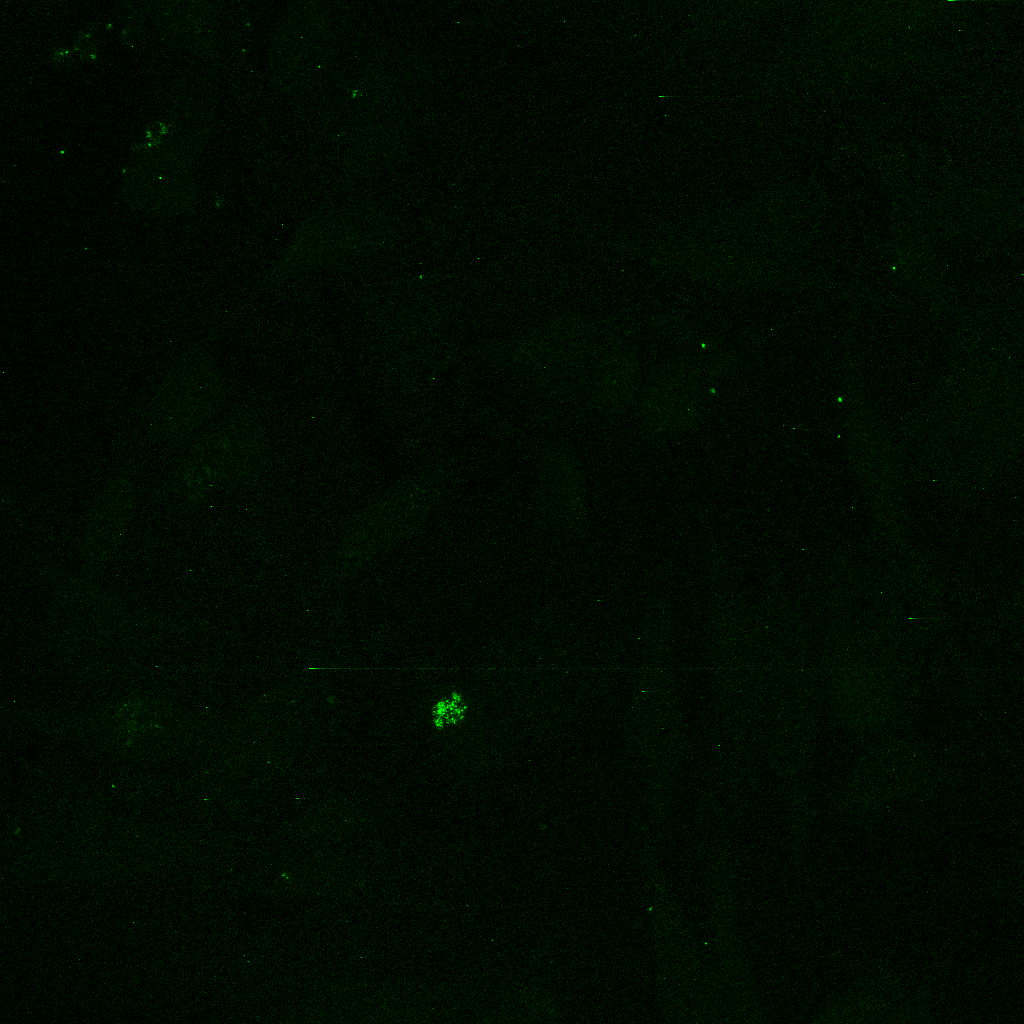

Supplement: Supplementary file 2 — Source data Fig. 1 [file 44318_2024_130_MOESM2_ESM.zip › Figure 1/1J/Fluvastatin/MPO.tif]

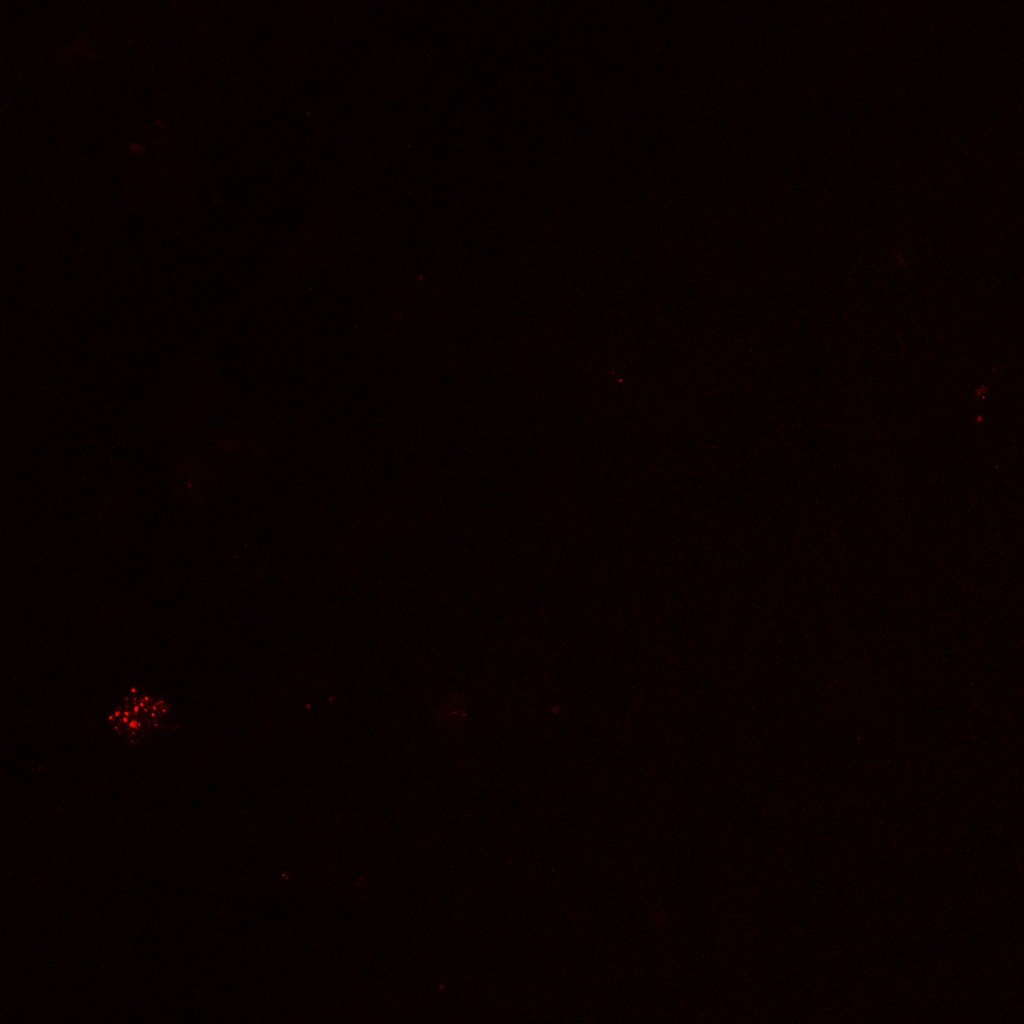

Supplement: Supplementary file 2 — Source data Fig. 1 [file 44318_2024_130_MOESM2_ESM.zip › Figure 1/1J/Fluvastatin/PKH26.tif]

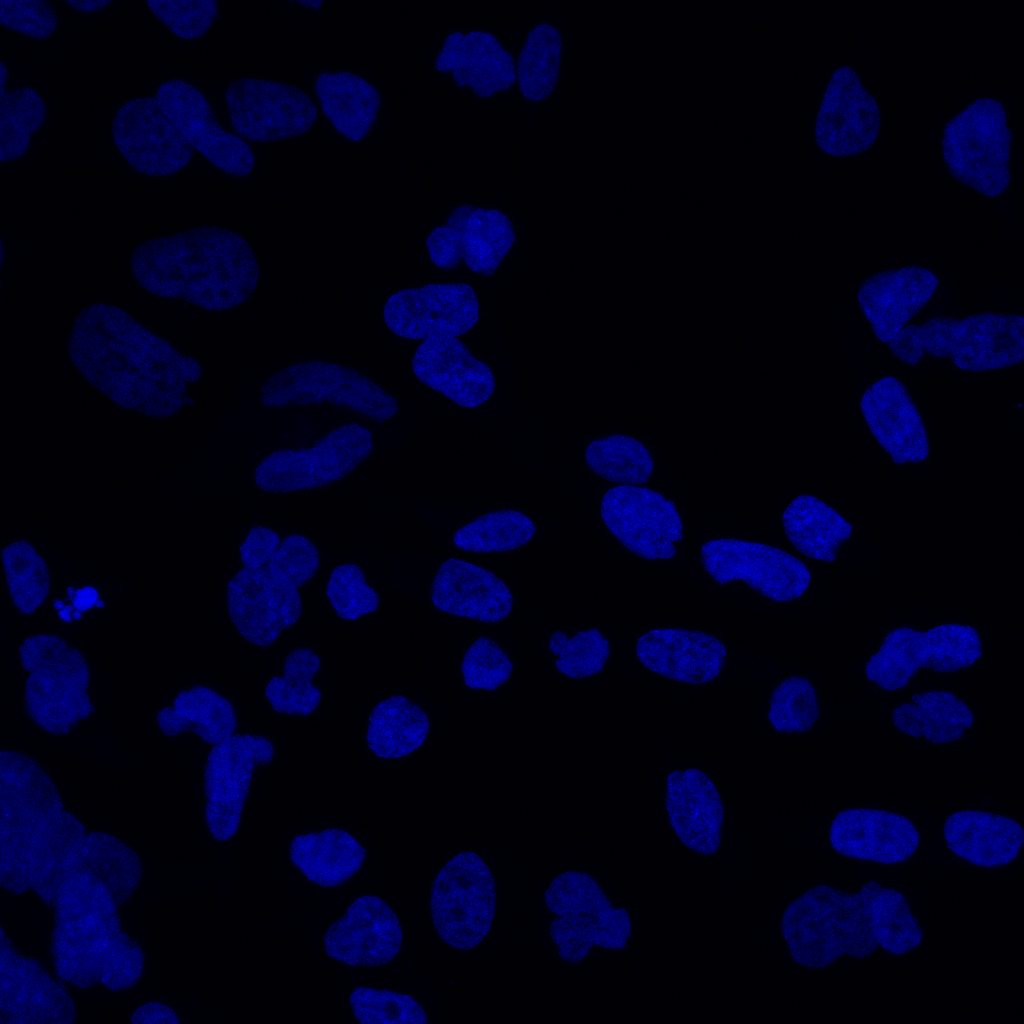

Supplement: Supplementary file 2 — Source data Fig. 1 [file 44318_2024_130_MOESM2_ESM.zip › Figure 1/1J/GGTI-298/DAPI.tif]

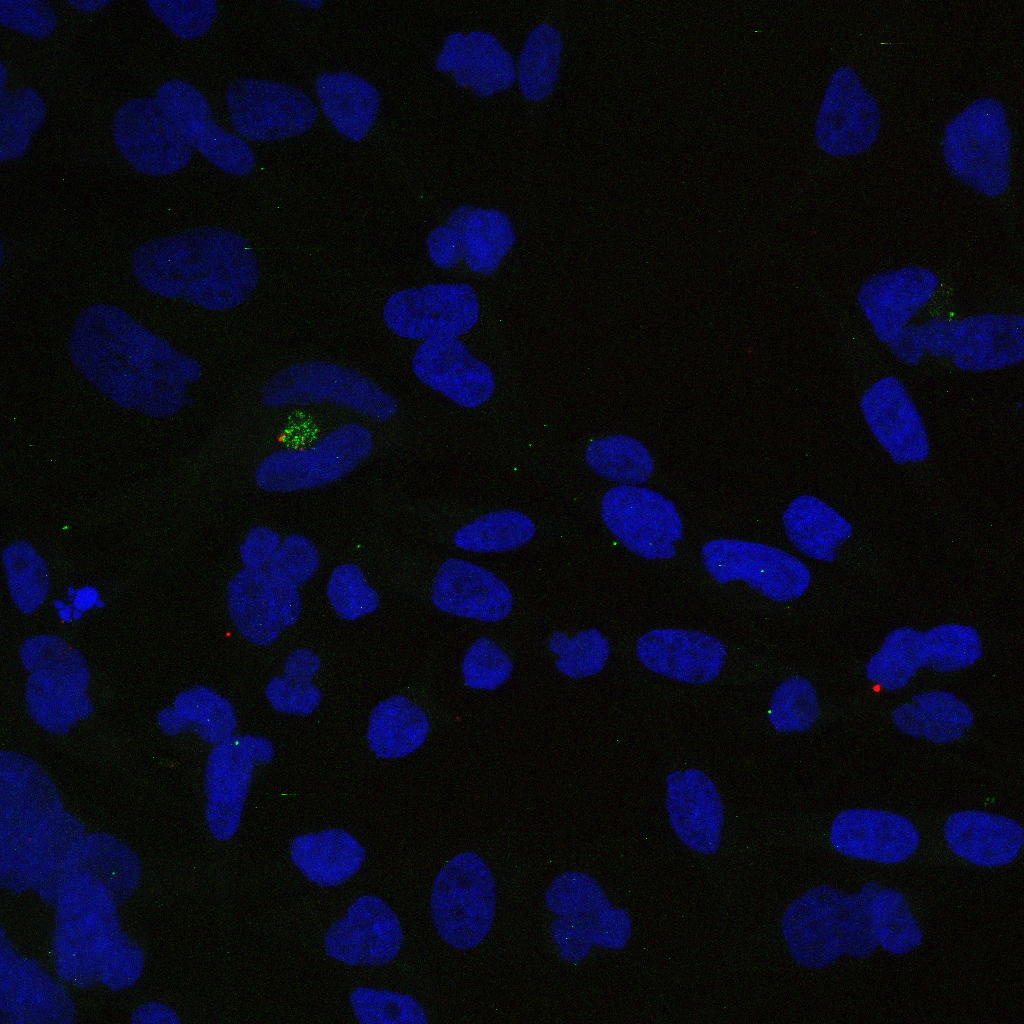

Supplement: Supplementary file 2 — Source data Fig. 1 [file 44318_2024_130_MOESM2_ESM.zip › Figure 1/1J/GGTI-298/Merged.tif]

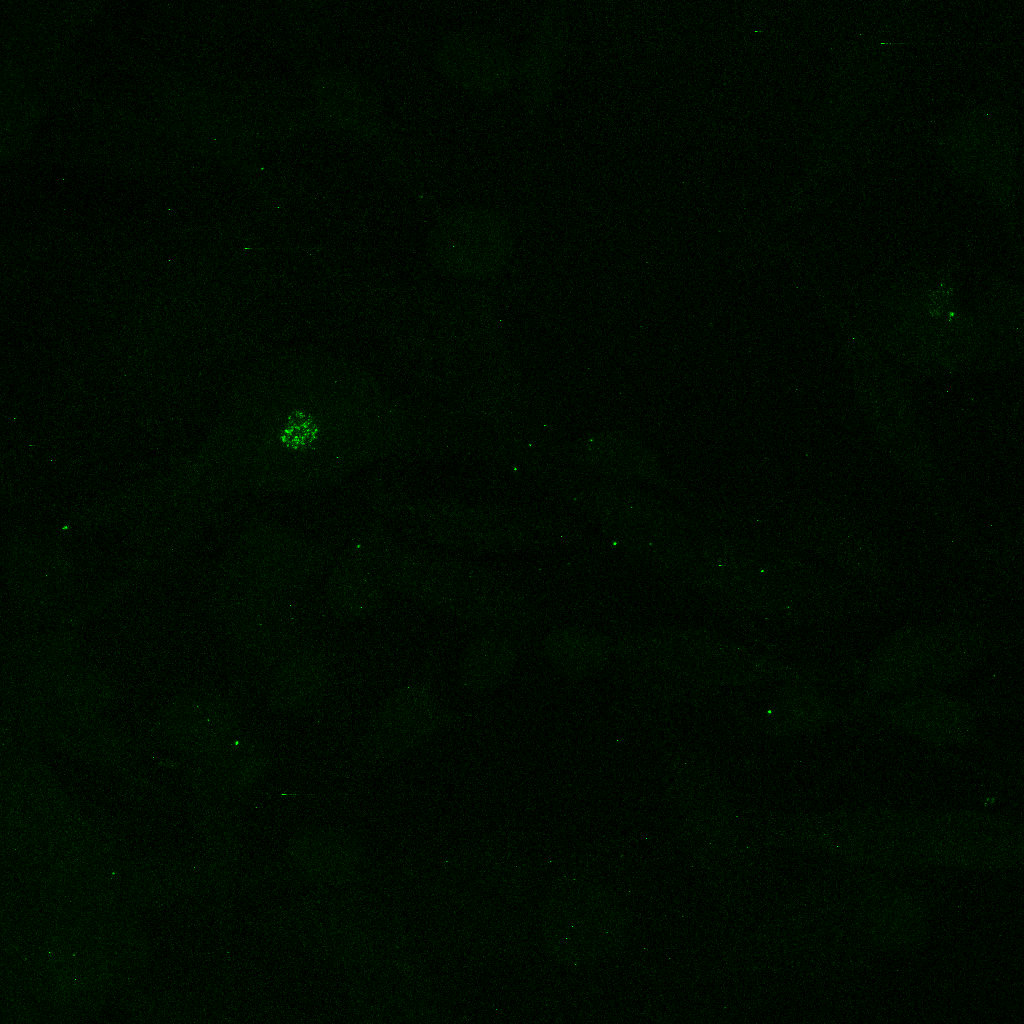

Supplement: Supplementary file 2 — Source data Fig. 1 [file 44318_2024_130_MOESM2_ESM.zip › Figure 1/1J/GGTI-298/MPO.tif]

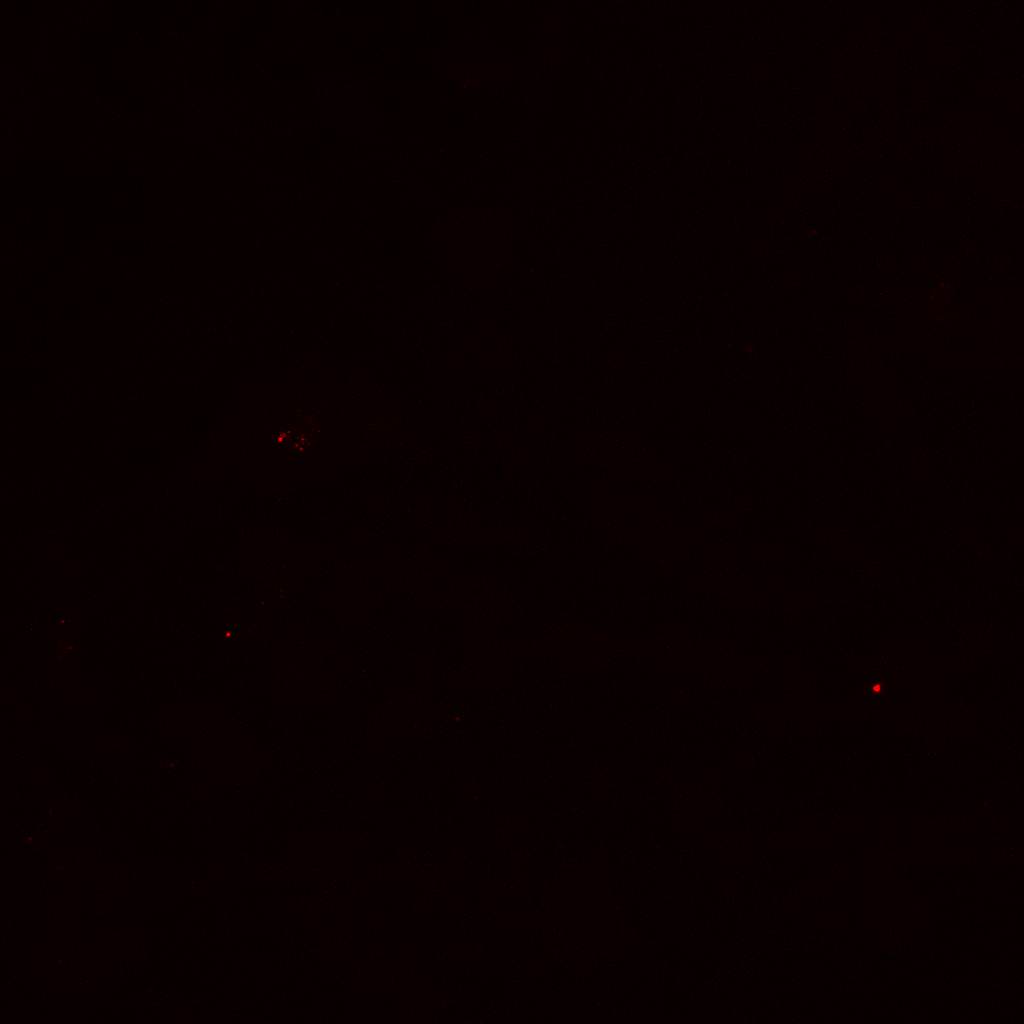

Supplement: Supplementary file 2 — Source data Fig. 1 [file 44318_2024_130_MOESM2_ESM.zip › Figure 1/1J/GGTI-298/PKH26.tif]

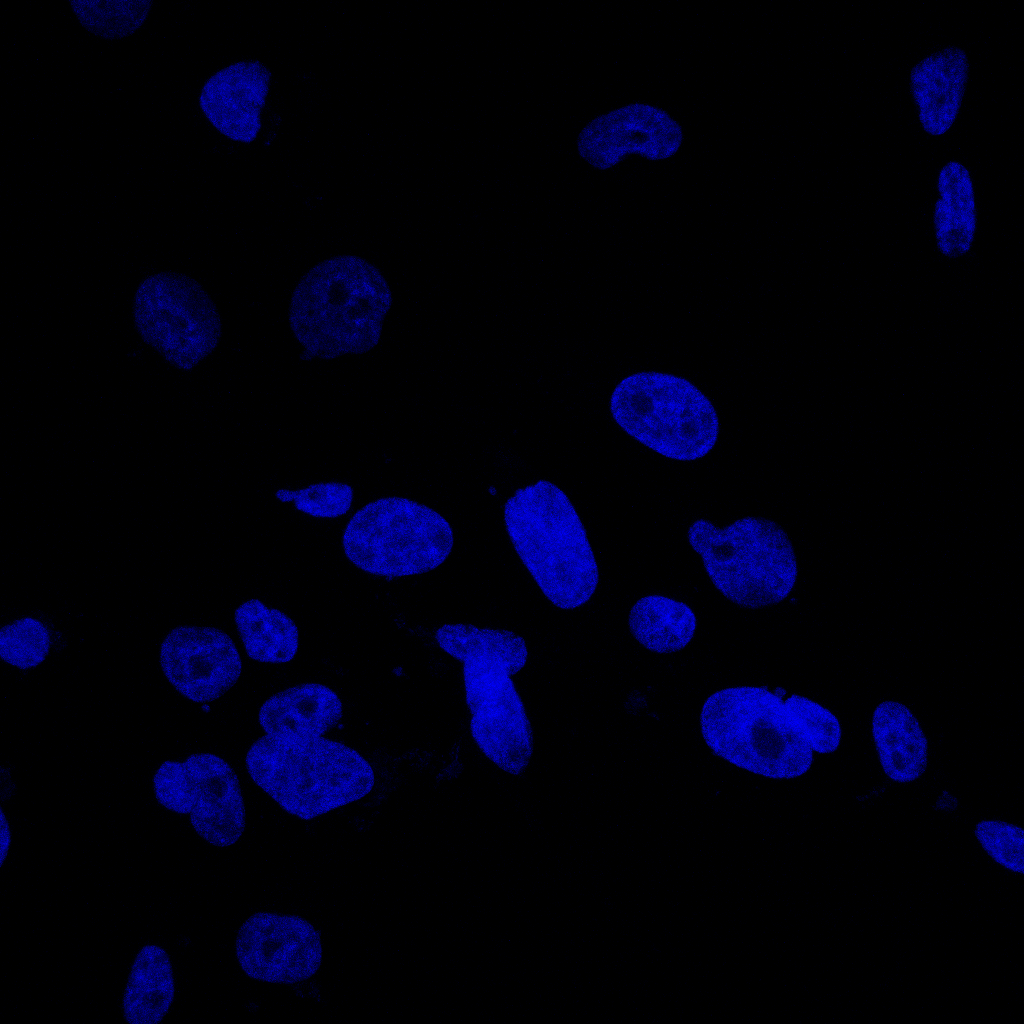

Supplement: Supplementary file 2 — Source data Fig. 1 [file 44318_2024_130_MOESM2_ESM.zip › Figure 1/1J/Simvastatin/DAPI.tif]

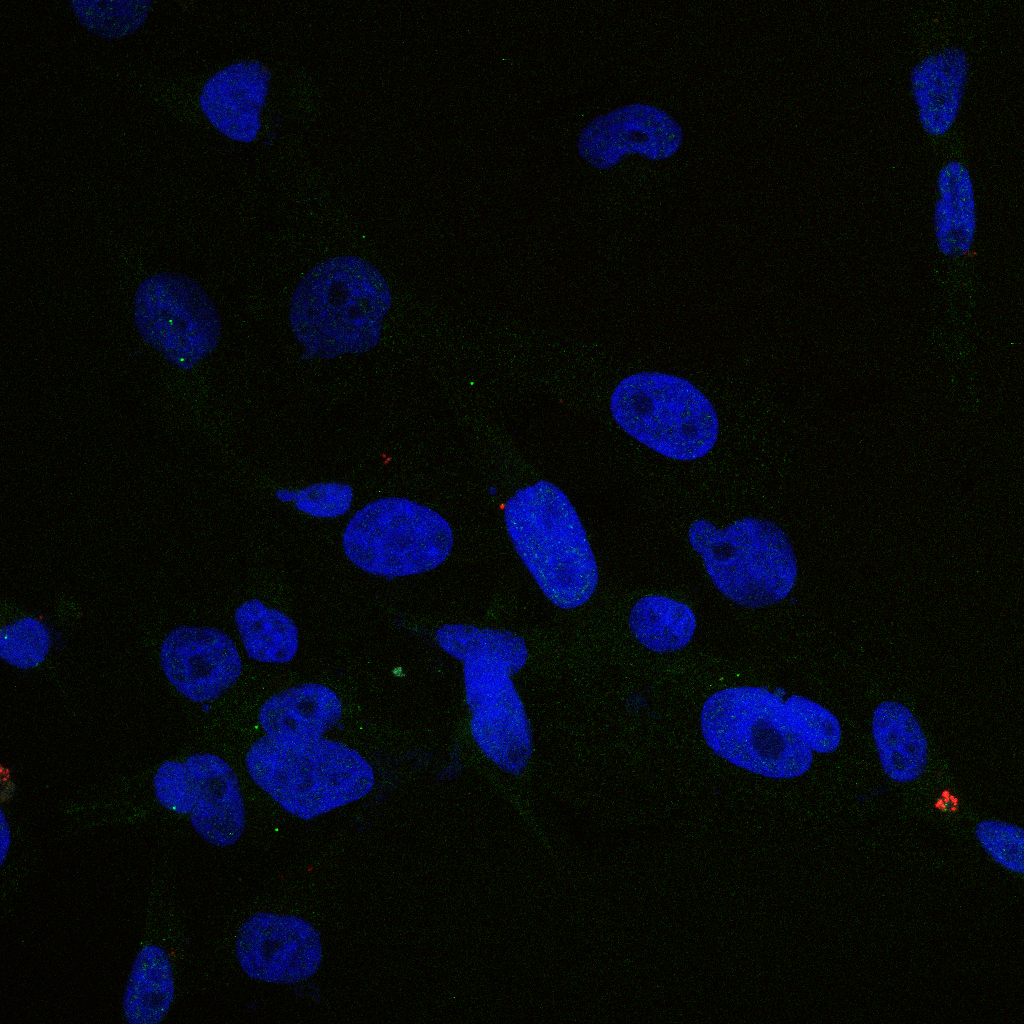

Supplement: Supplementary file 2 — Source data Fig. 1 [file 44318_2024_130_MOESM2_ESM.zip › Figure 1/1J/Simvastatin/Merged.tif]

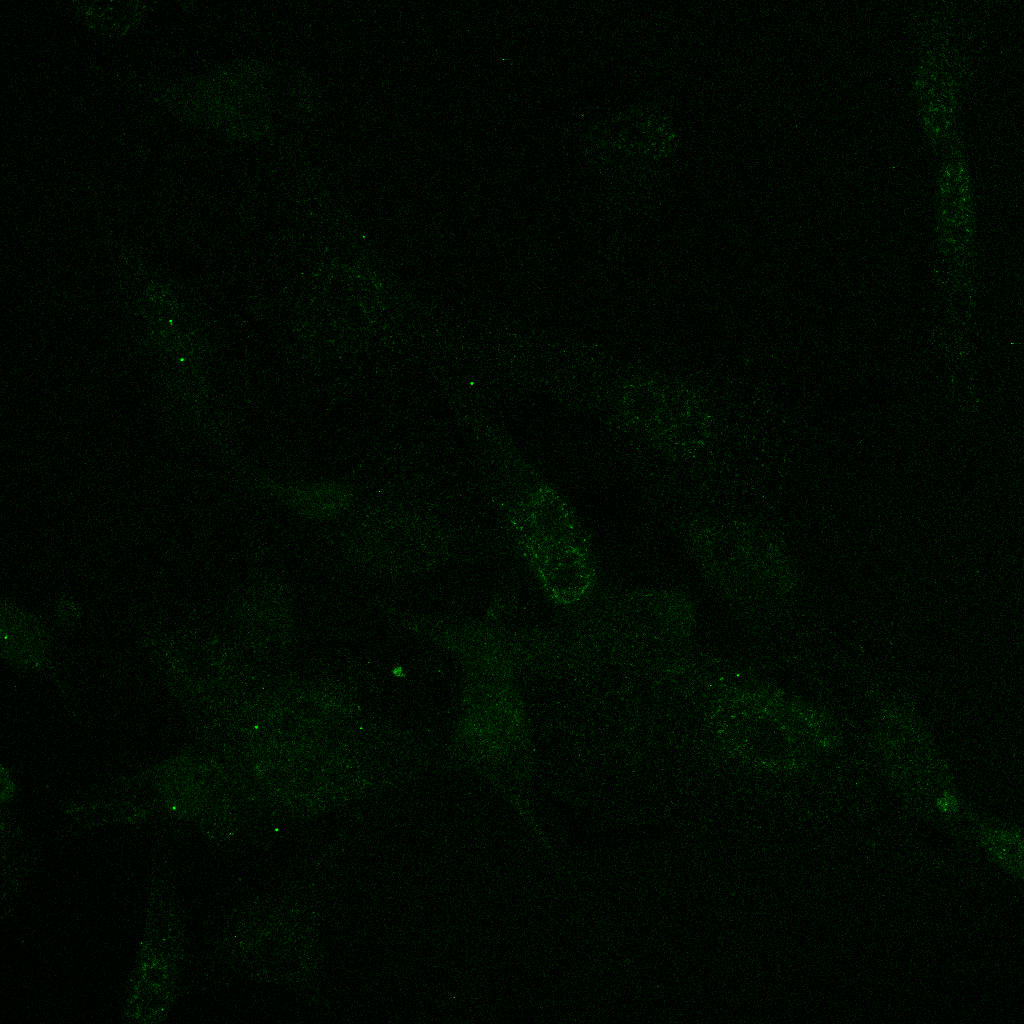

Supplement: Supplementary file 2 — Source data Fig. 1 [file 44318_2024_130_MOESM2_ESM.zip › Figure 1/1J/Simvastatin/MPO.tif]

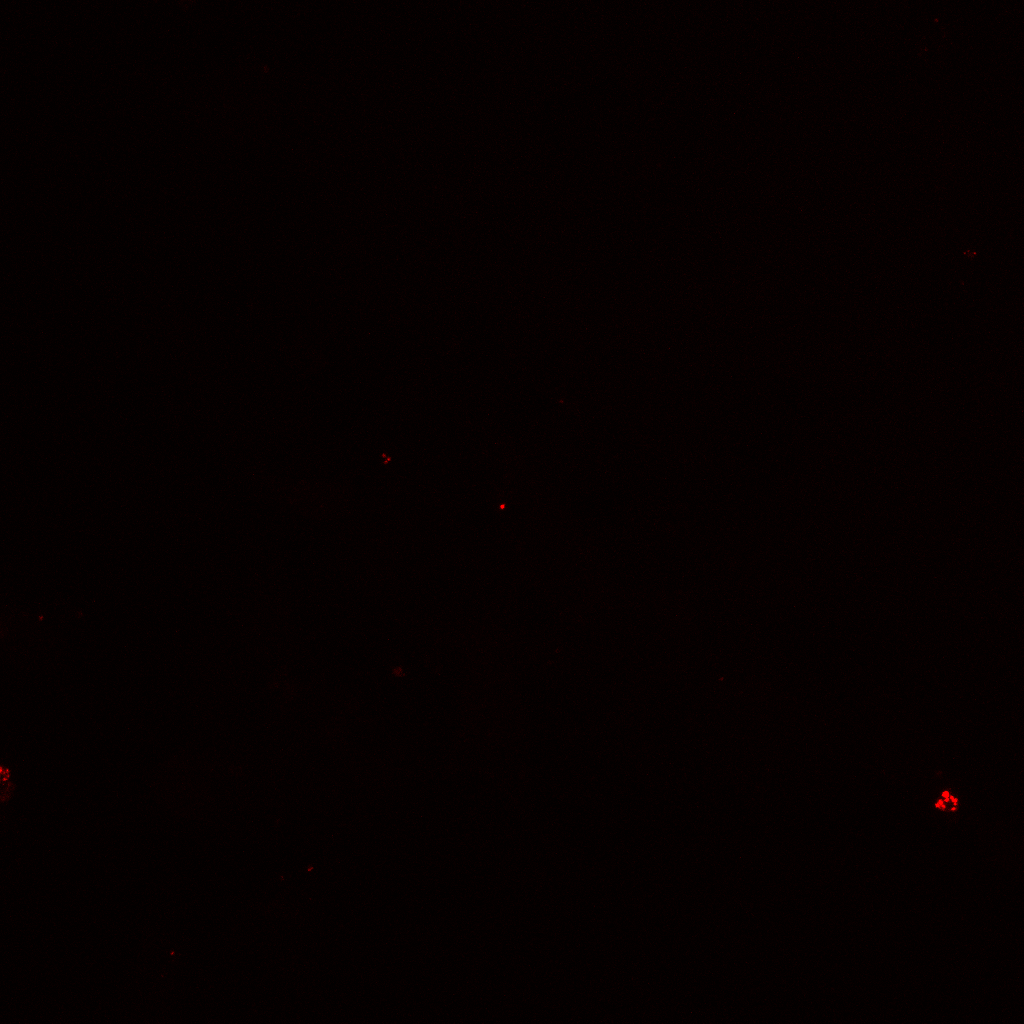

Supplement: Supplementary file 2 — Source data Fig. 1 [file 44318_2024_130_MOESM2_ESM.zip › Figure 1/1J/Simvastatin/PKH26.tif]

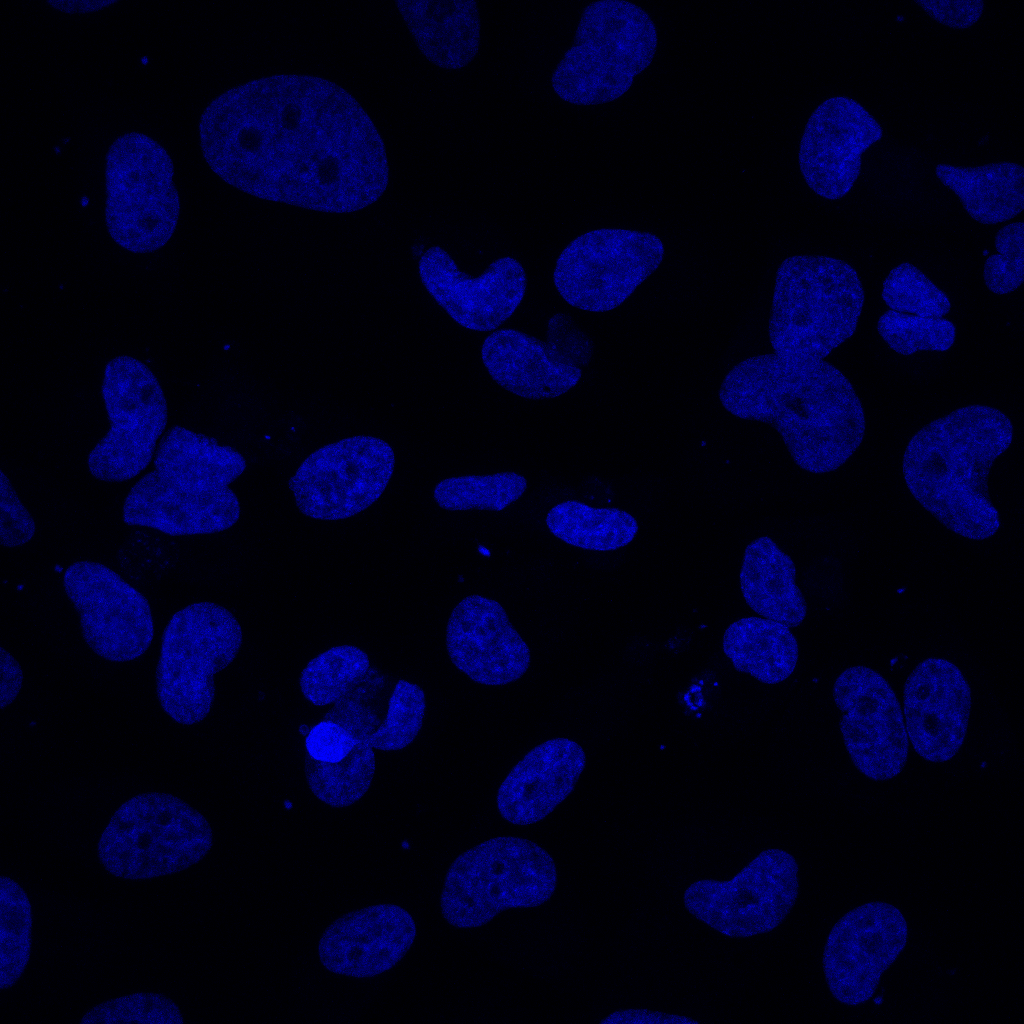

Supplement: Supplementary file 2 — Source data Fig. 1 [file 44318_2024_130_MOESM2_ESM.zip › Figure 1/1J/YM-53601/DAPI.tif]

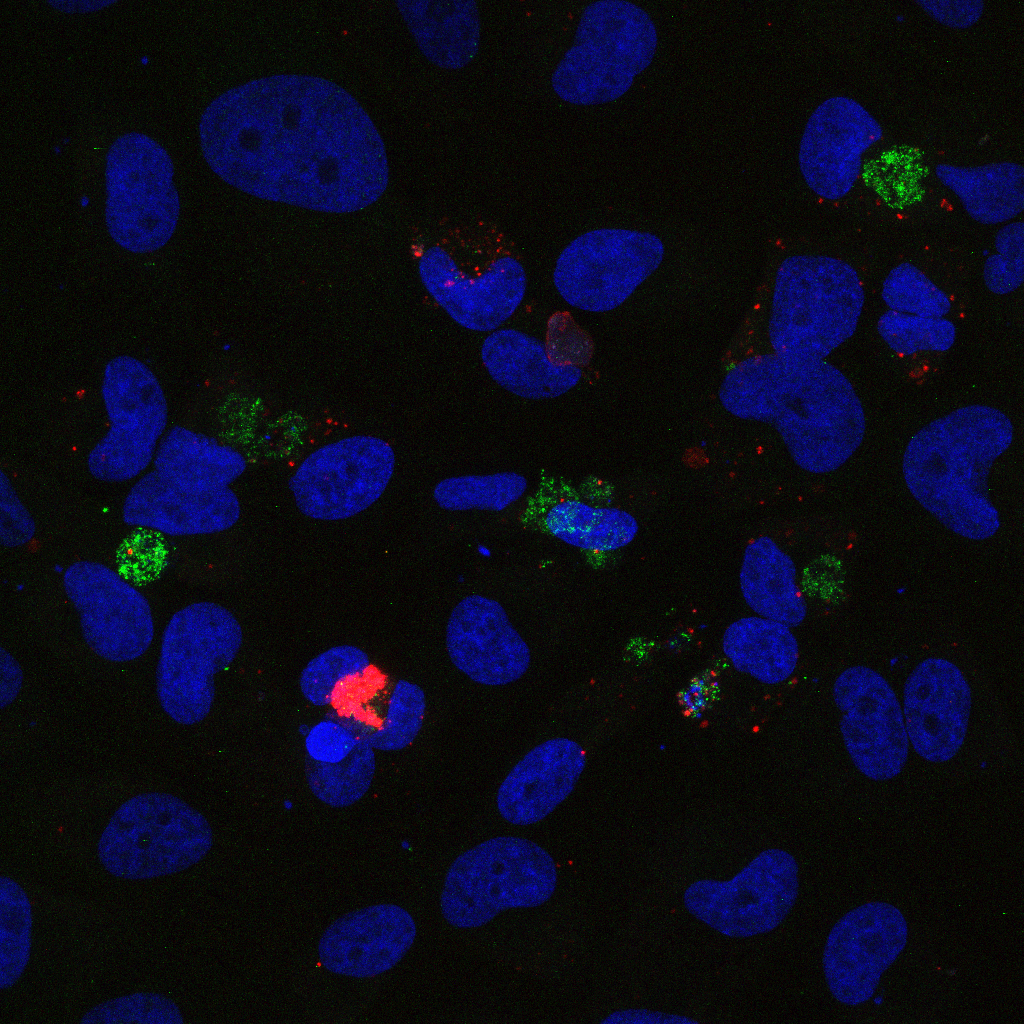

Supplement: Supplementary file 2 — Source data Fig. 1 [file 44318_2024_130_MOESM2_ESM.zip › Figure 1/1J/YM-53601/Merged.tif]

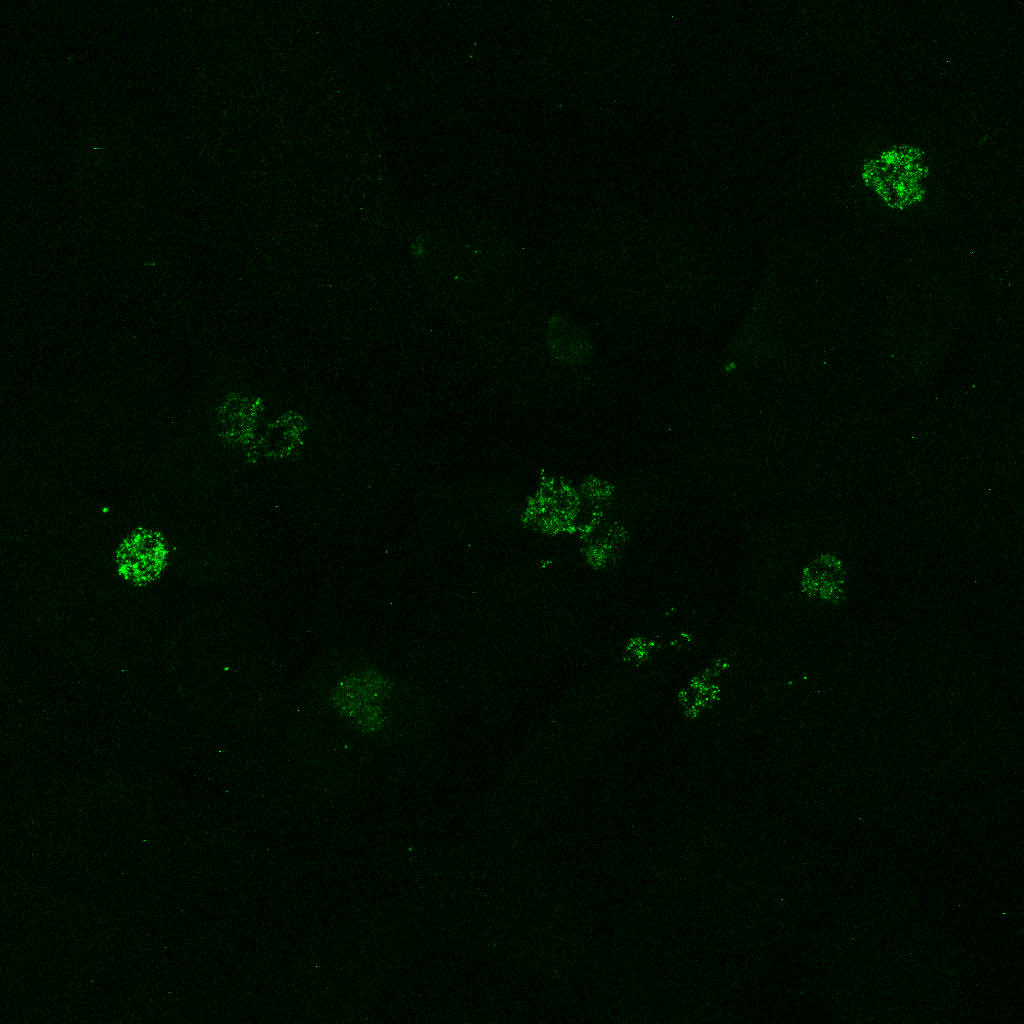

Supplement: Supplementary file 2 — Source data Fig. 1 [file 44318_2024_130_MOESM2_ESM.zip › Figure 1/1J/YM-53601/MPO.tif]

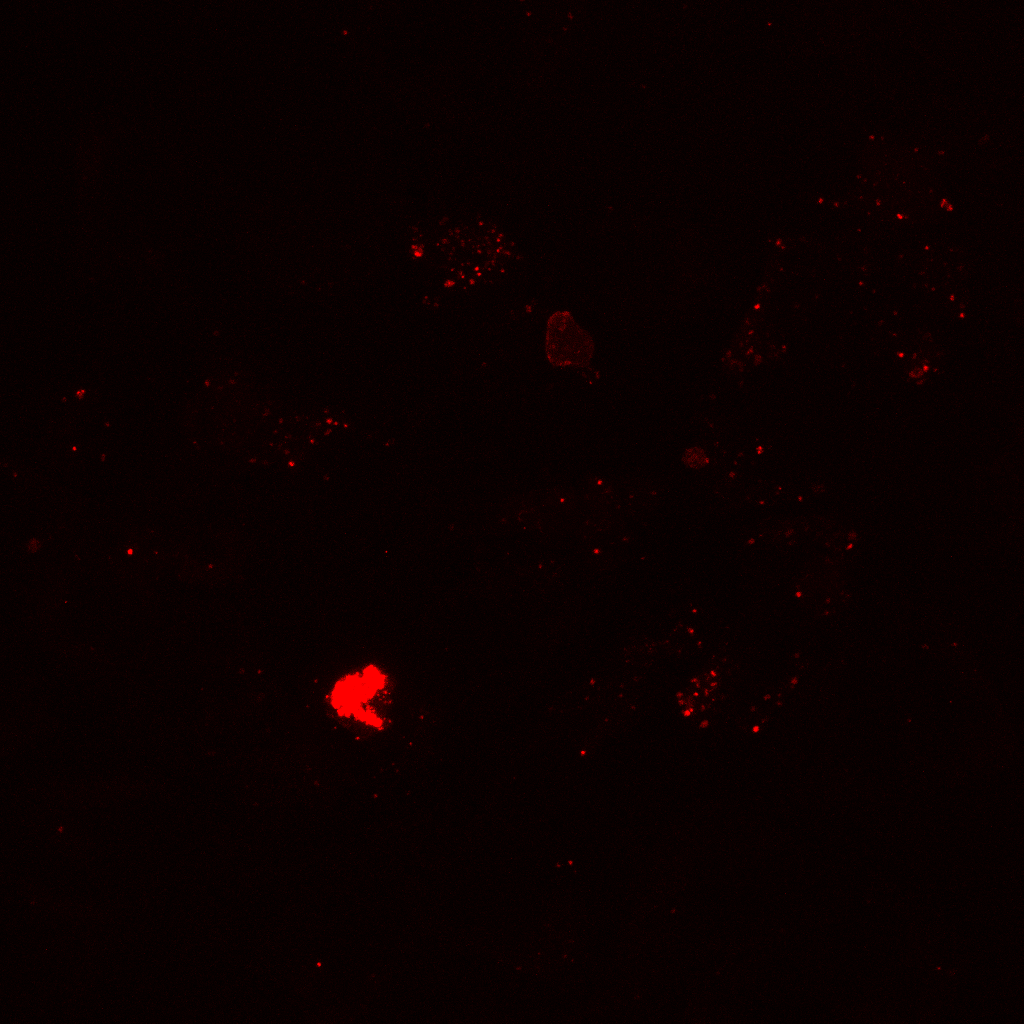

Supplement: Supplementary file 2 — Source data Fig. 1 [file 44318_2024_130_MOESM2_ESM.zip › Figure 1/1J/YM-53601/PKH26.tif]

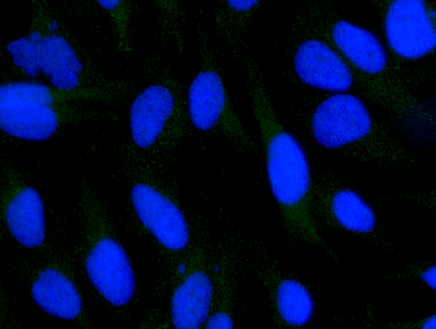

Supplement: Supplementary file 3 — Source data Fig. 2 [file 44318_2024_130_MOESM3_ESM.zip › Figure 2/2D/2D/Alone/55-56-57 merged cropped.tif]

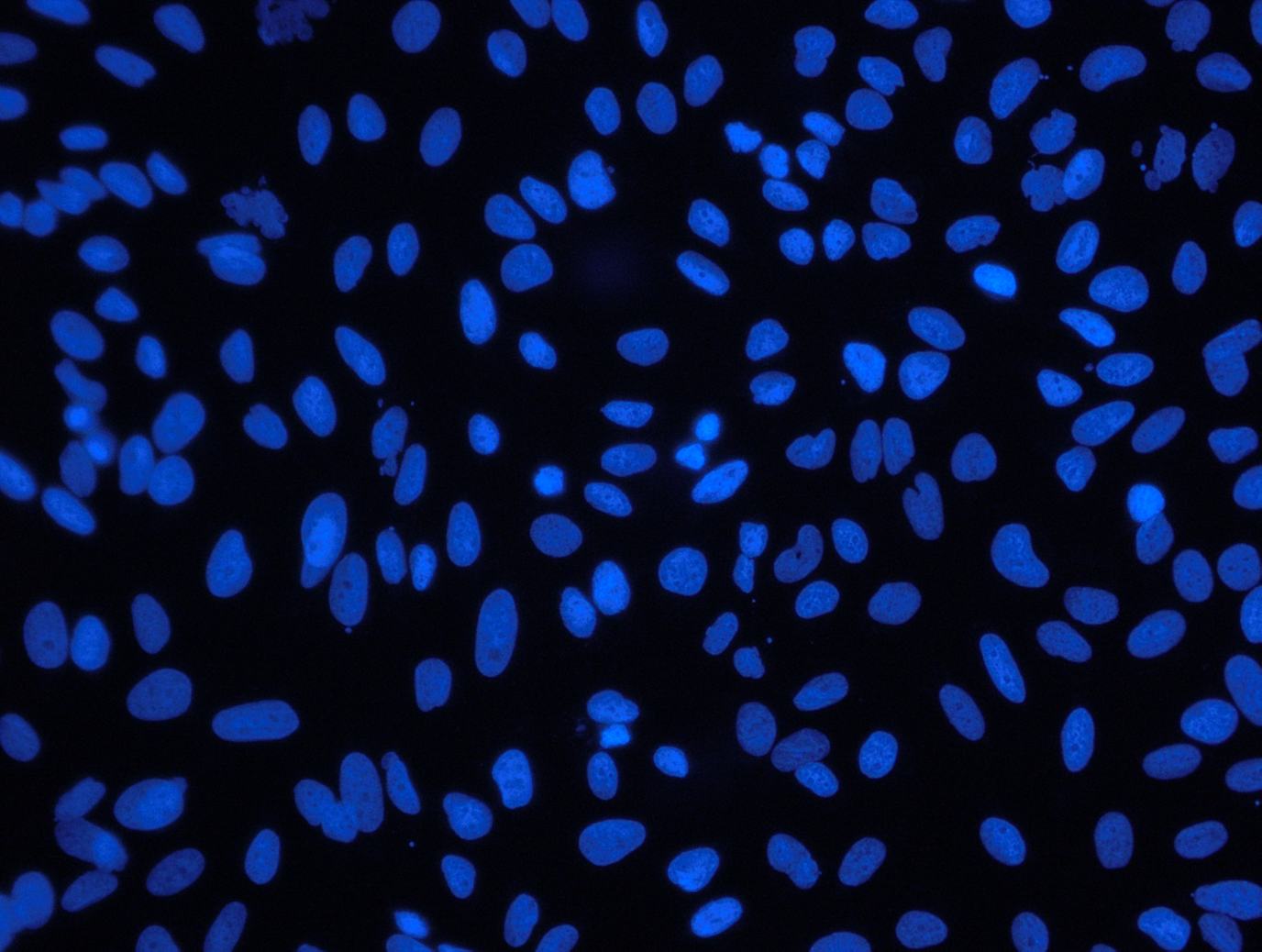

Supplement: Supplementary file 3 — Source data Fig. 2 [file 44318_2024_130_MOESM3_ESM.zip › Figure 2/2D/2D/Alone/Image_55.tif]

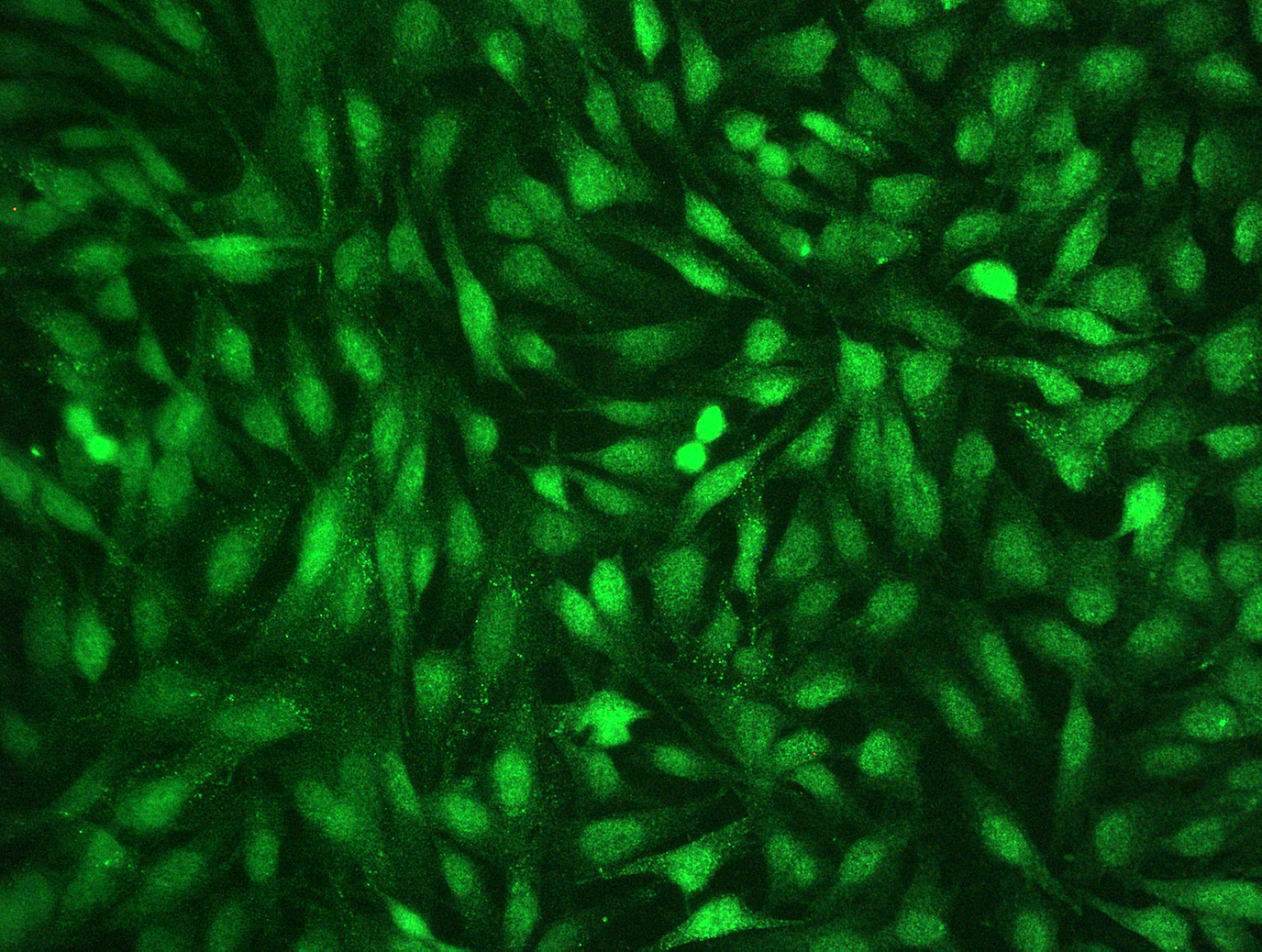

Supplement: Supplementary file 3 — Source data Fig. 2 [file 44318_2024_130_MOESM3_ESM.zip › Figure 2/2D/2D/Alone/Image_56.tif]

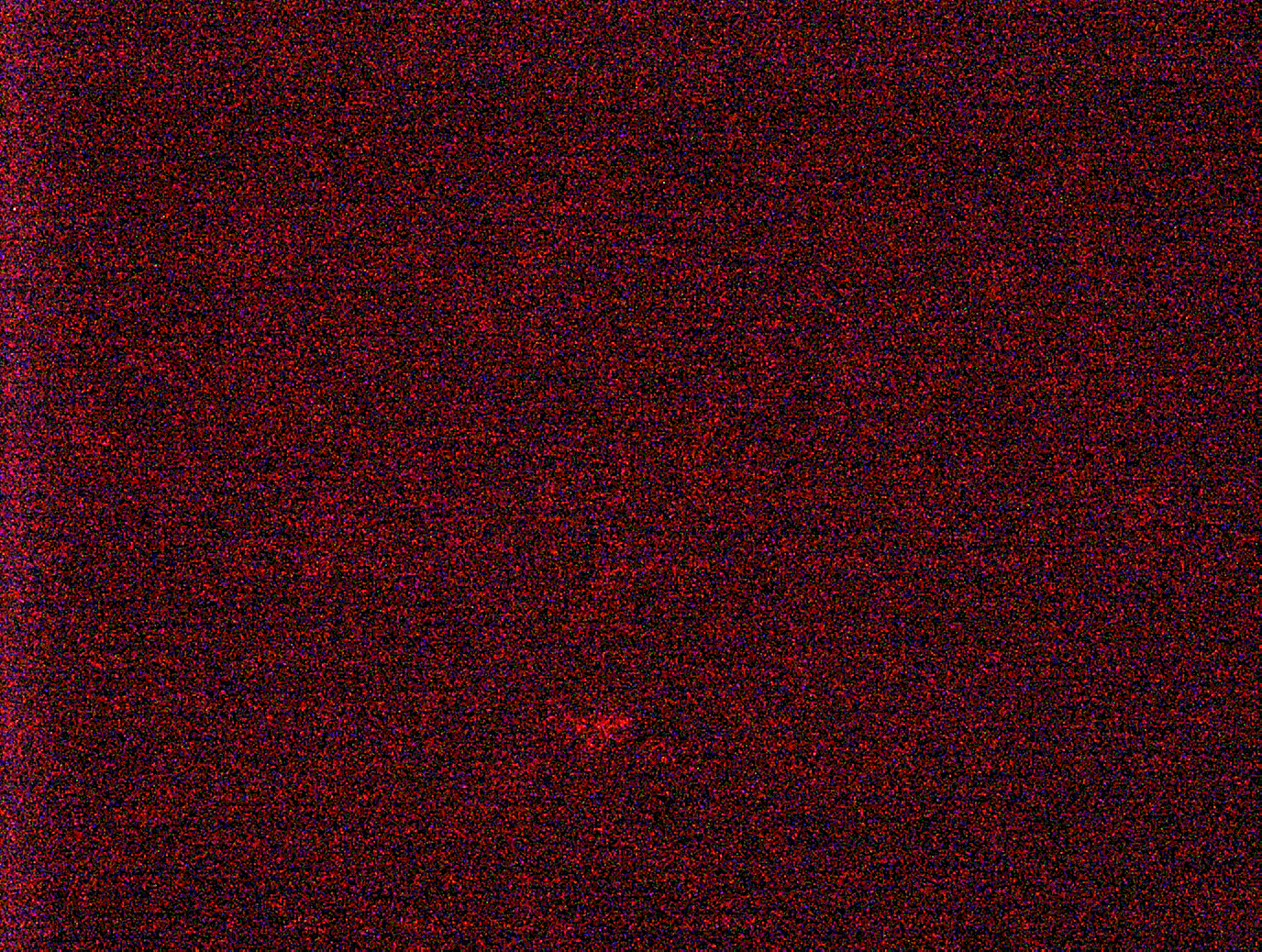

Supplement: Supplementary file 3 — Source data Fig. 2 [file 44318_2024_130_MOESM3_ESM.zip › Figure 2/2D/2D/Alone/Image_57.tif]

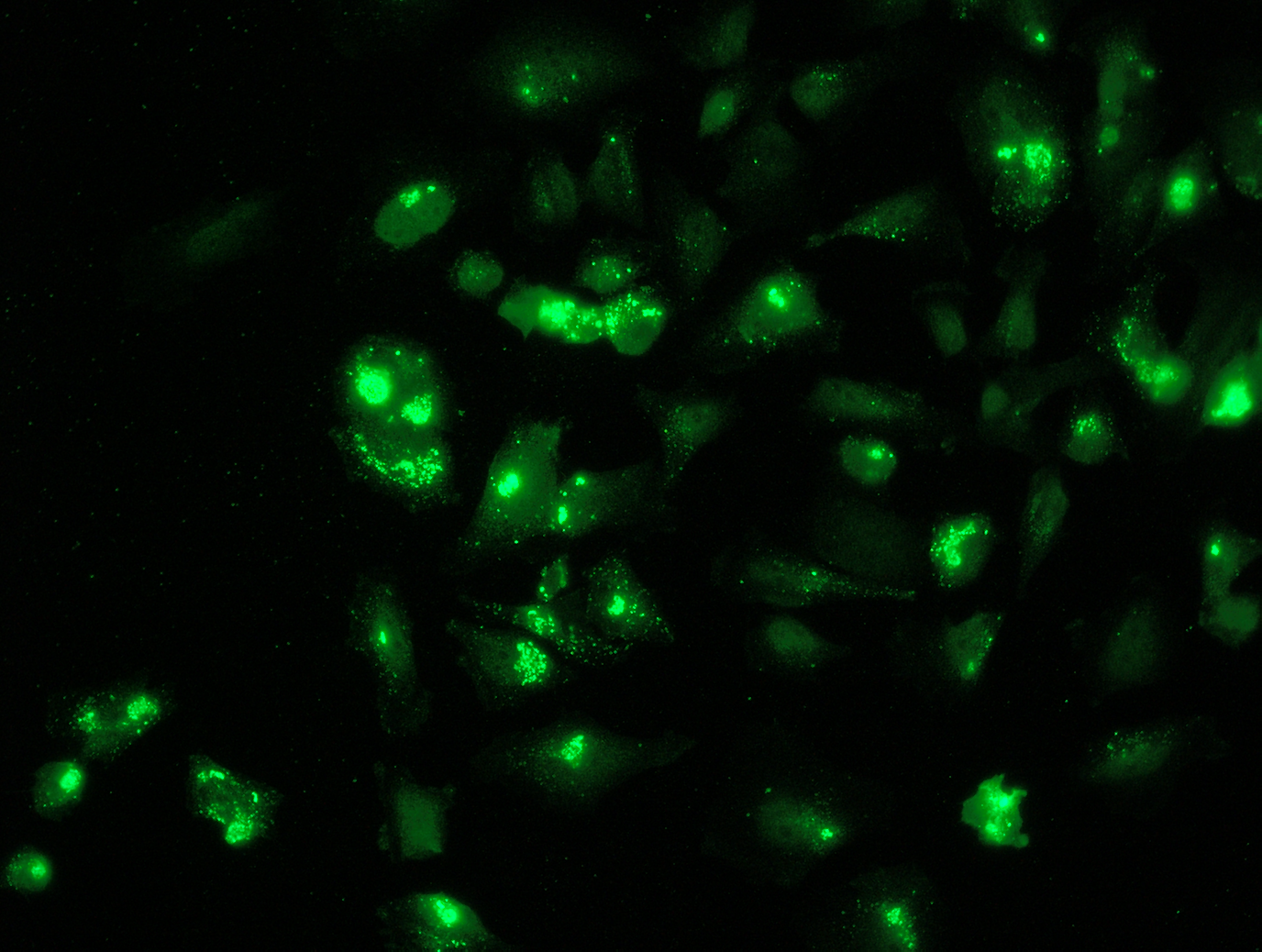

Supplement: Supplementary file 3 — Source data Fig. 2 [file 44318_2024_130_MOESM3_ESM.zip › Figure 2/2D/2D/w Cell/Image_78.tif]

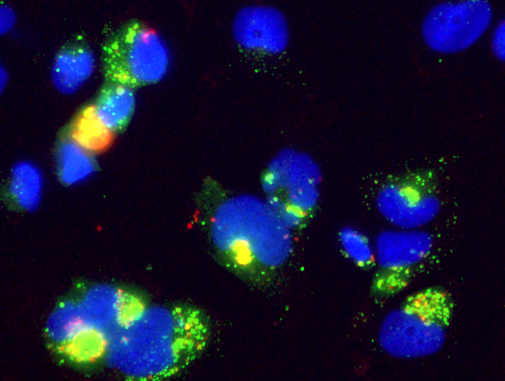

Supplement: Supplementary file 3 — Source data Fig. 2 [file 44318_2024_130_MOESM3_ESM.zip › Figure 2/2D/2D/w Cell/Image_78-79-80 merged cropped.tif]

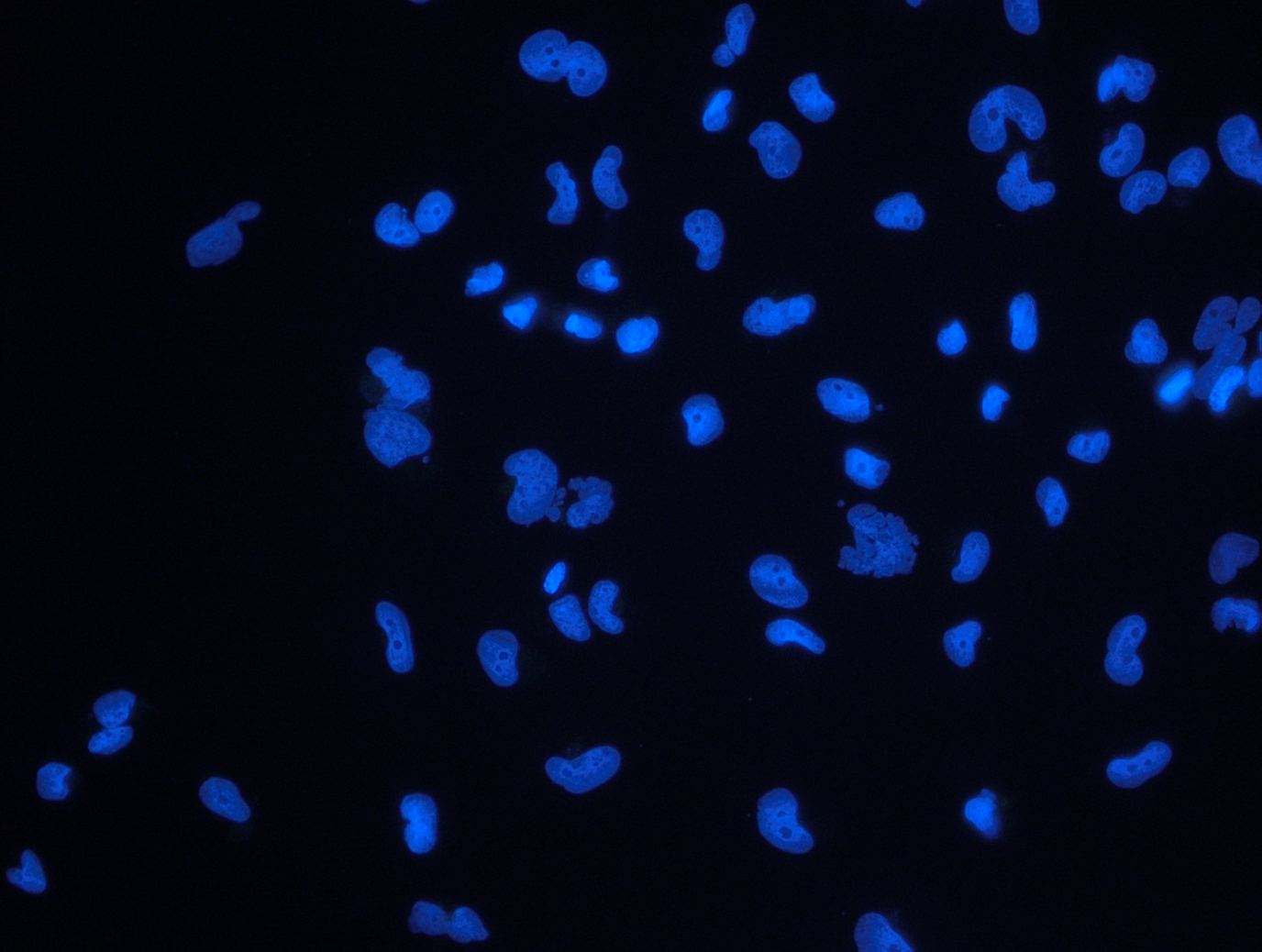

Supplement: Supplementary file 3 — Source data Fig. 2 [file 44318_2024_130_MOESM3_ESM.zip › Figure 2/2D/2D/w Cell/Image_79.tif]

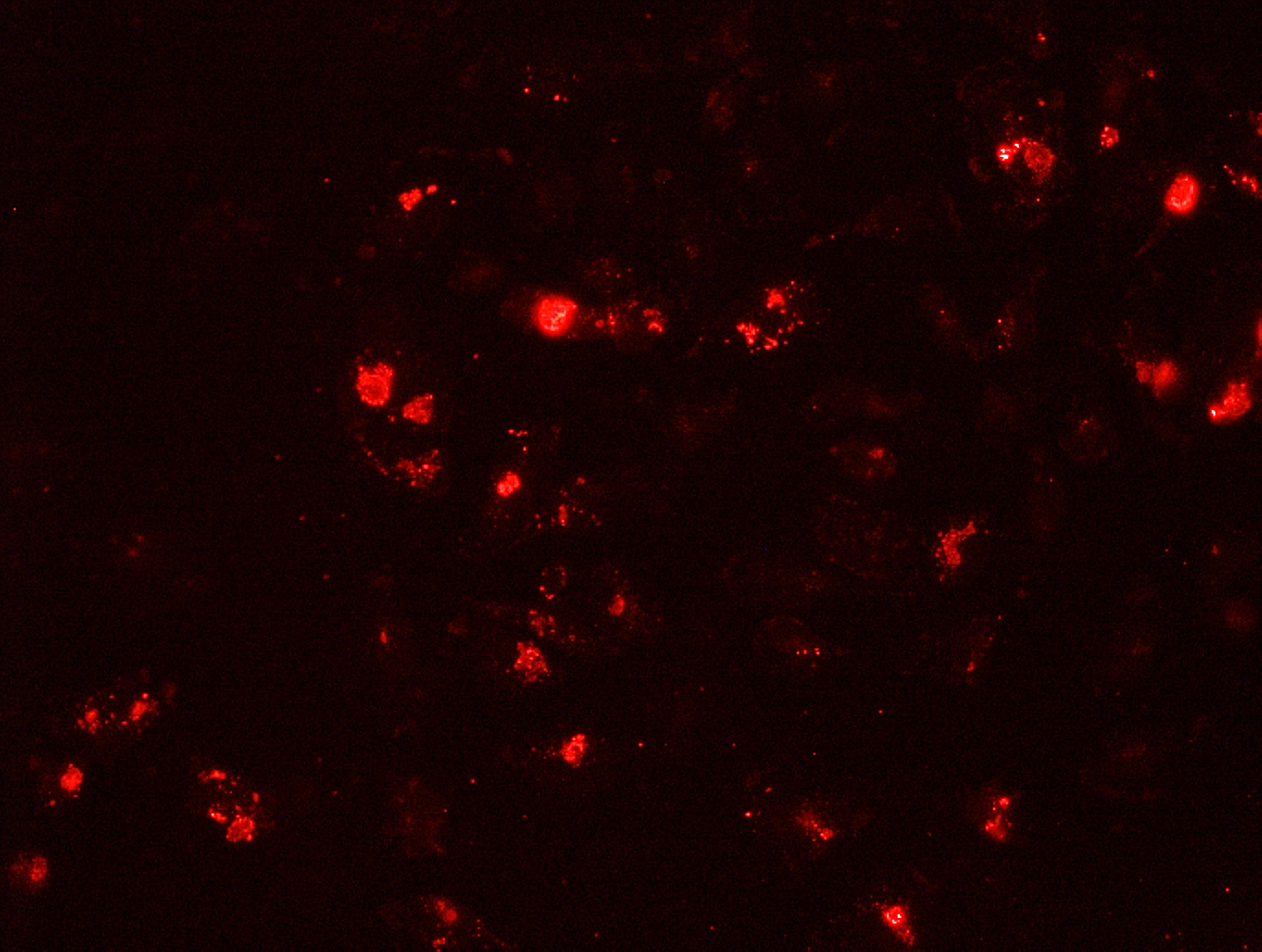

Supplement: Supplementary file 3 — Source data Fig. 2 [file 44318_2024_130_MOESM3_ESM.zip › Figure 2/2D/2D/w Cell/Image_80.tif]

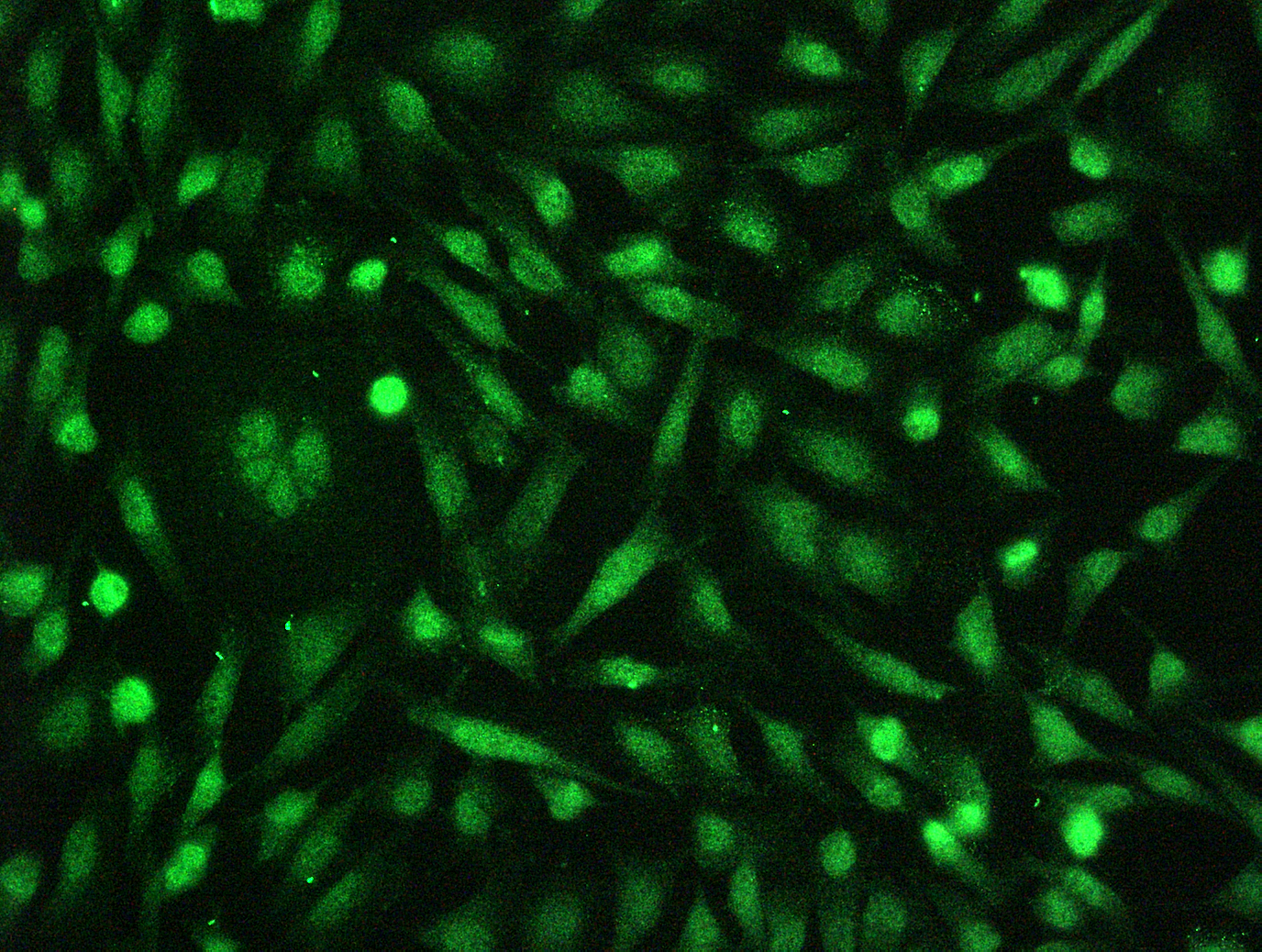

Supplement: Supplementary file 3 — Source data Fig. 2 [file 44318_2024_130_MOESM3_ESM.zip › Figure 2/2D/2D/w CM/Image_87.tif]

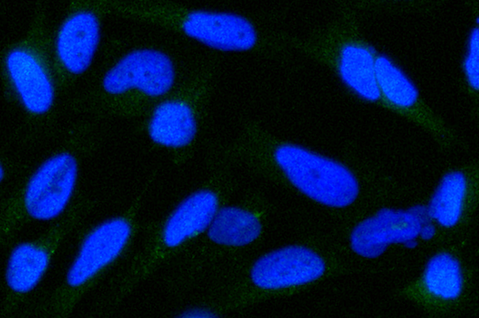

Supplement: Supplementary file 3 — Source data Fig. 2 [file 44318_2024_130_MOESM3_ESM.zip › Figure 2/2D/2D/w CM/Image_87-88-89 merged cropped.tif]

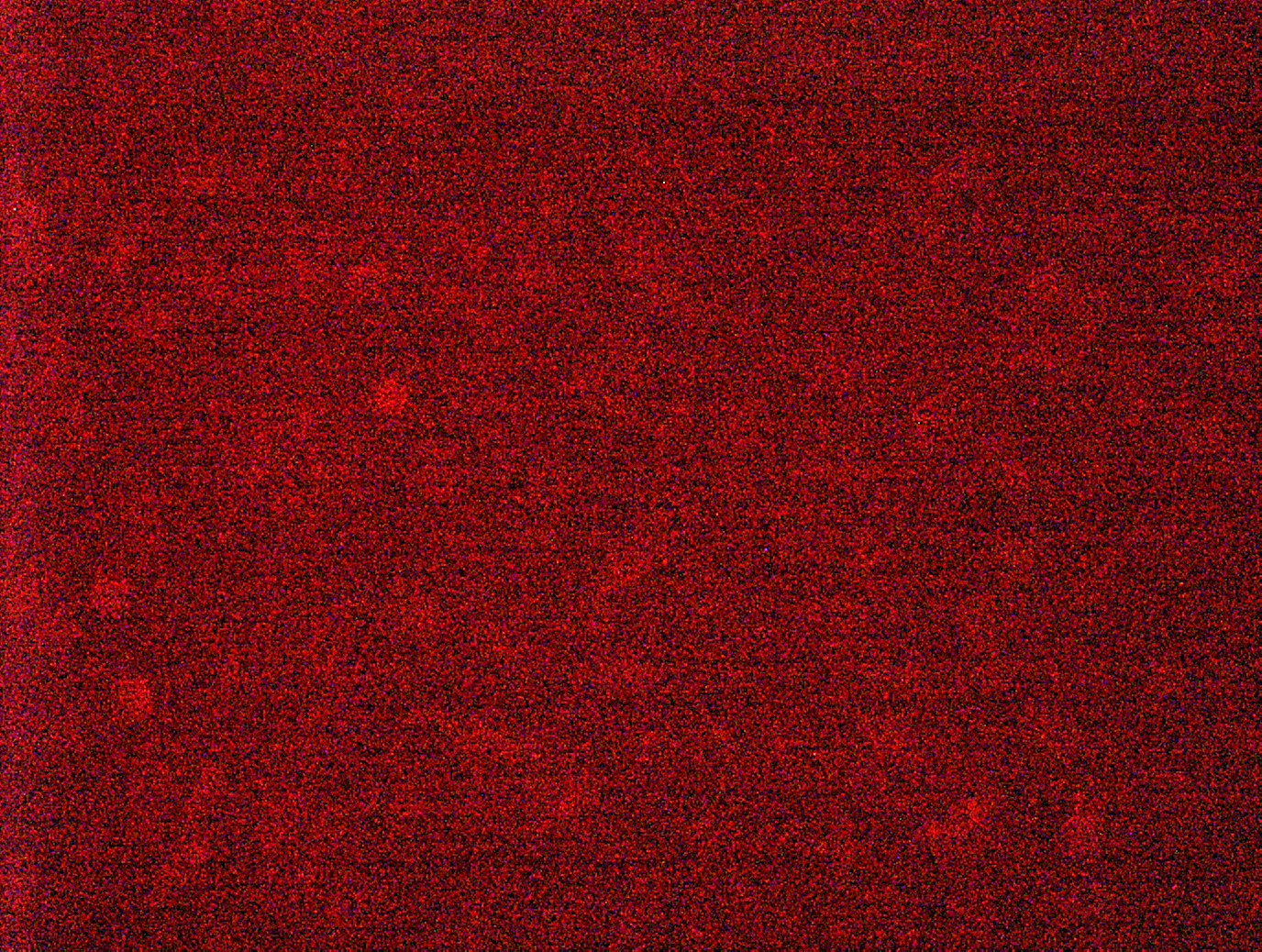

Supplement: Supplementary file 3 — Source data Fig. 2 [file 44318_2024_130_MOESM3_ESM.zip › Figure 2/2D/2D/w CM/Image_88.tif]

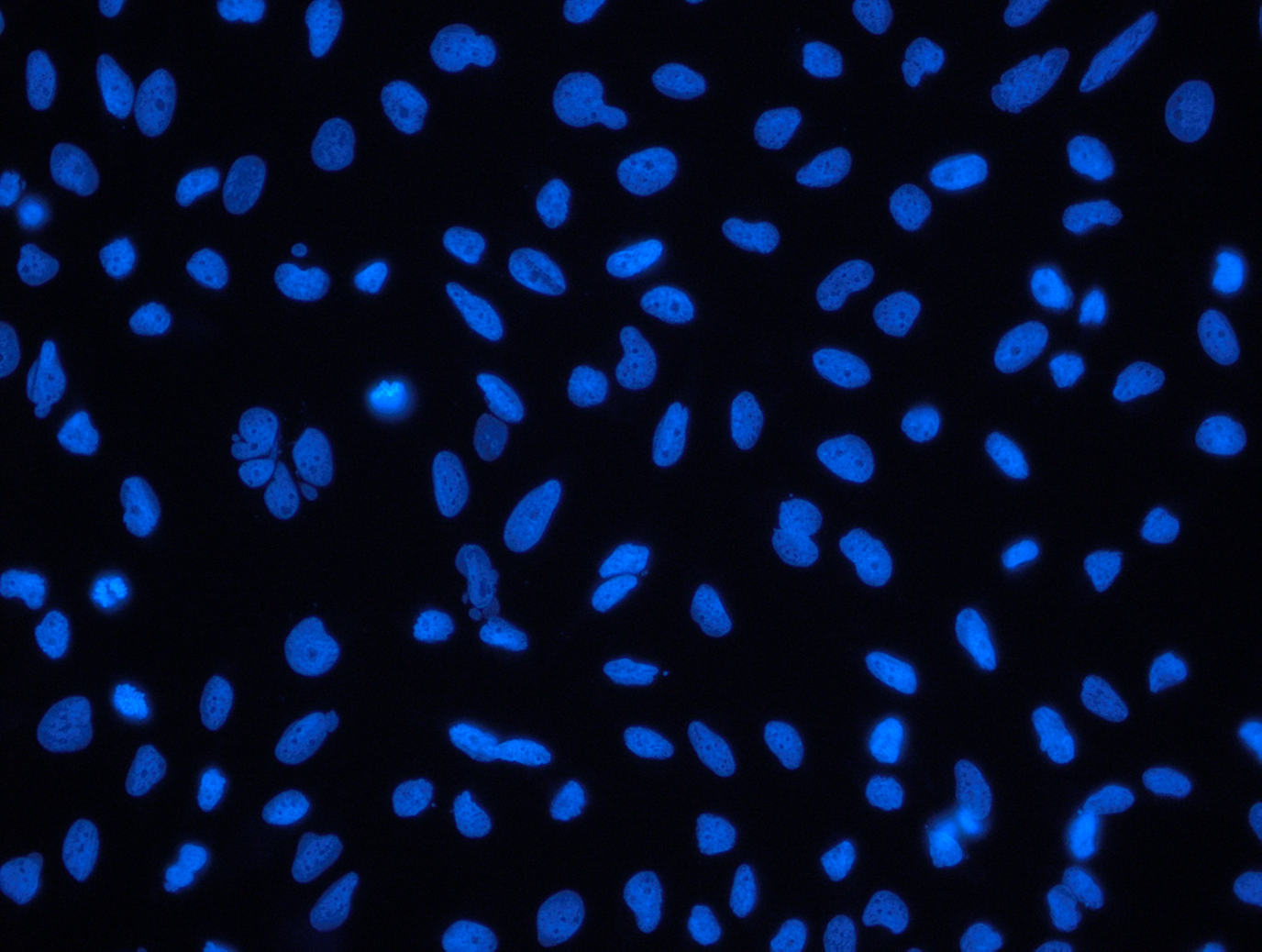

Supplement: Supplementary file 3 — Source data Fig. 2 [file 44318_2024_130_MOESM3_ESM.zip › Figure 2/2D/2D/w CM/Image_89.tif]

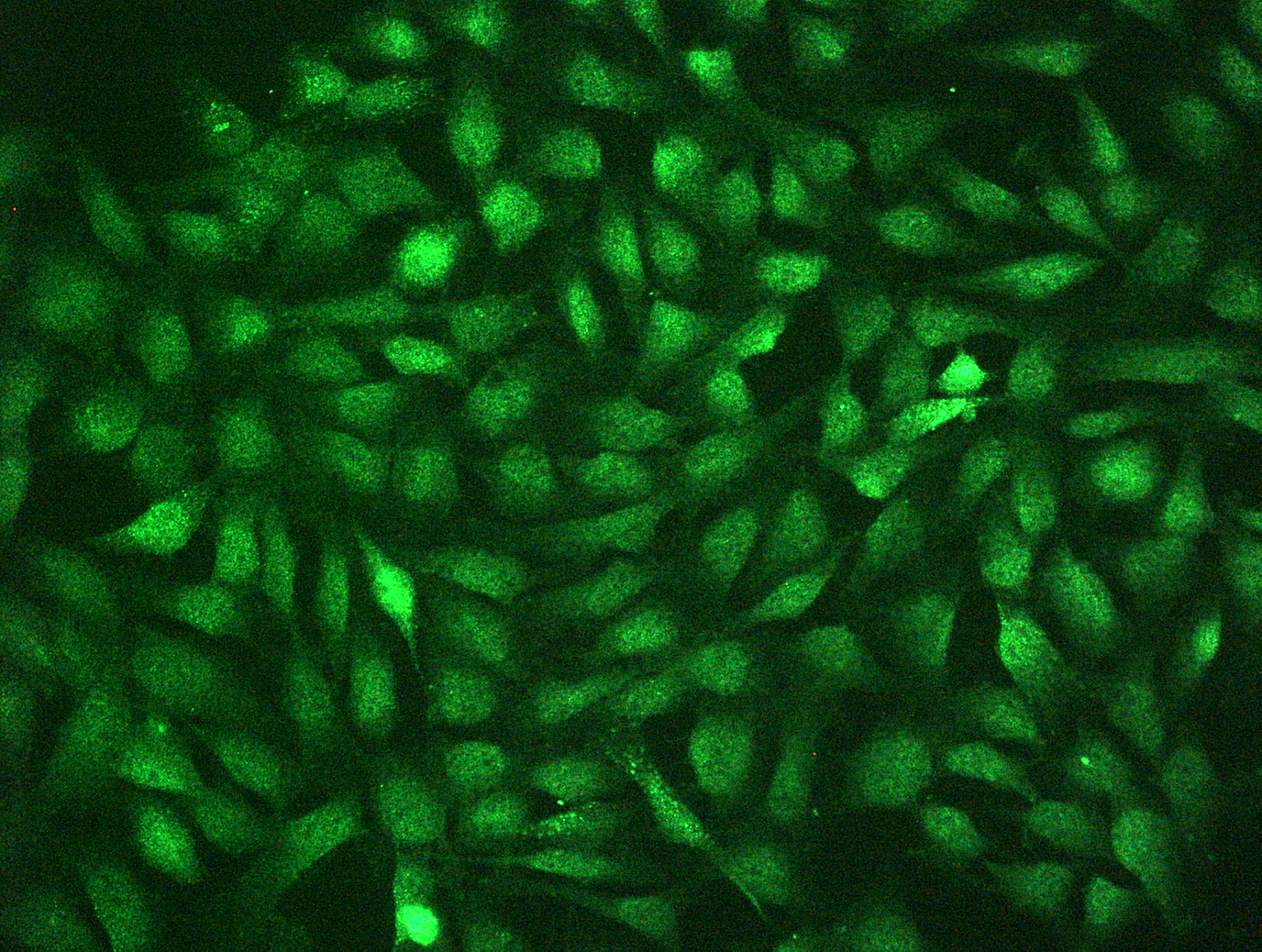

Supplement: Supplementary file 3 — Source data Fig. 2 [file 44318_2024_130_MOESM3_ESM.zip › Figure 2/2D/2D/w Insert/Image_428.tif]

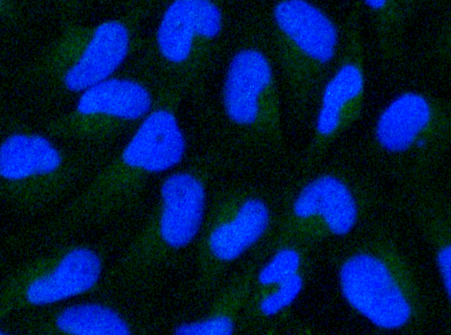

Supplement: Supplementary file 3 — Source data Fig. 2 [file 44318_2024_130_MOESM3_ESM.zip › Figure 2/2D/2D/w Insert/Image_428-429-430 merged cropped.tif]

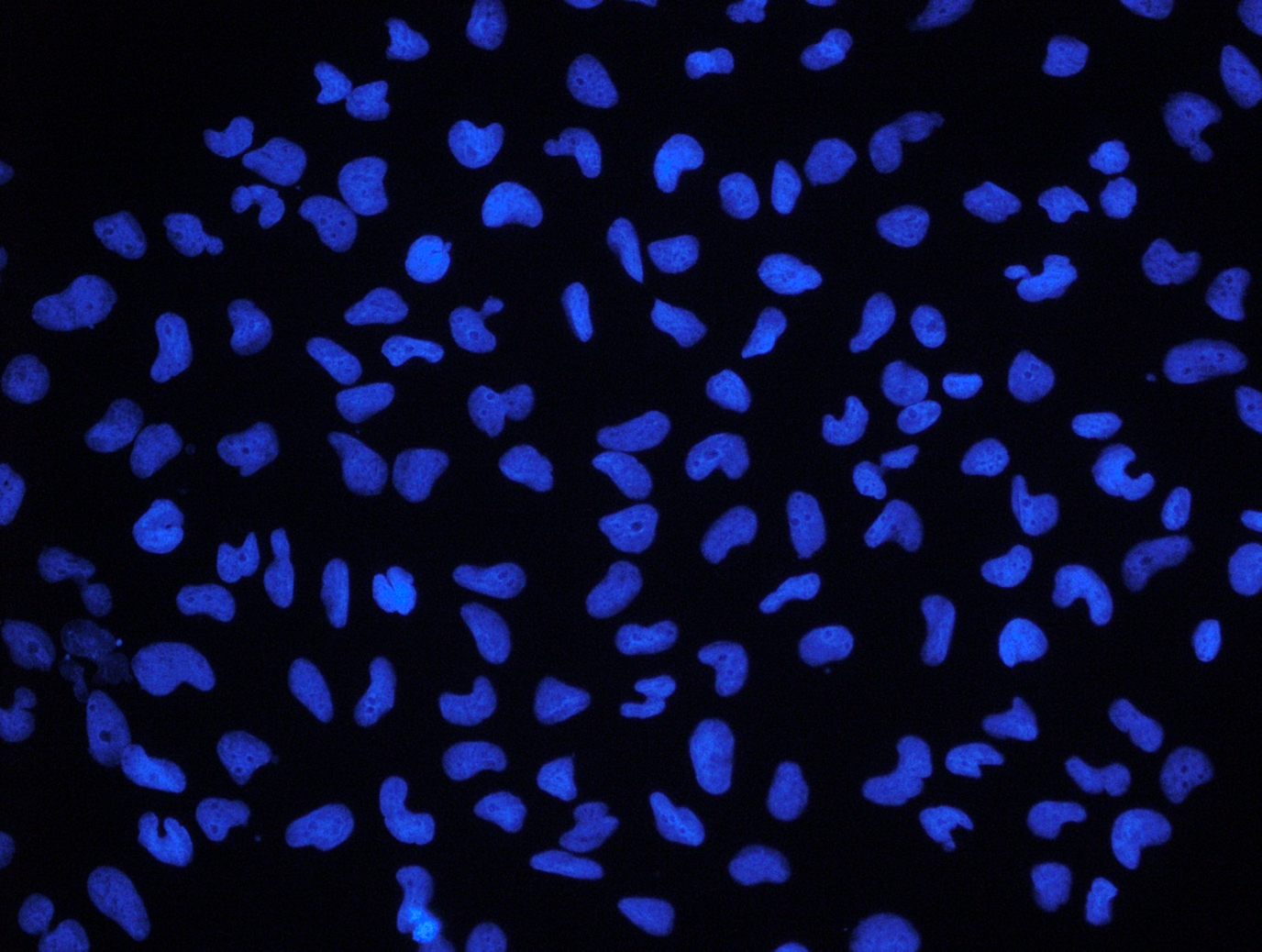

Supplement: Supplementary file 3 — Source data Fig. 2 [file 44318_2024_130_MOESM3_ESM.zip › Figure 2/2D/2D/w Insert/Image_429.tif]

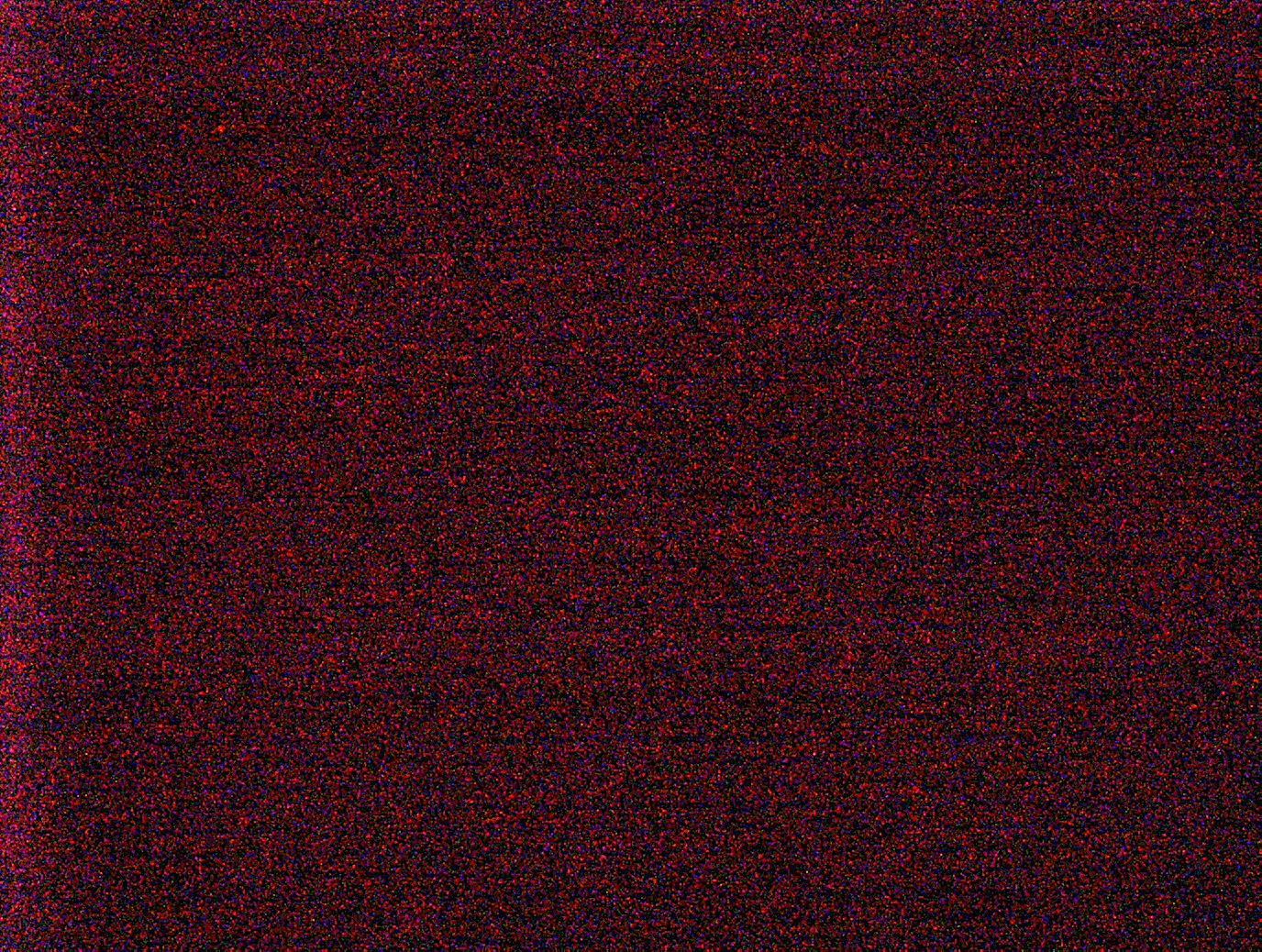

Supplement: Supplementary file 3 — Source data Fig. 2 [file 44318_2024_130_MOESM3_ESM.zip › Figure 2/2D/2D/w Insert/Image_430.tif]

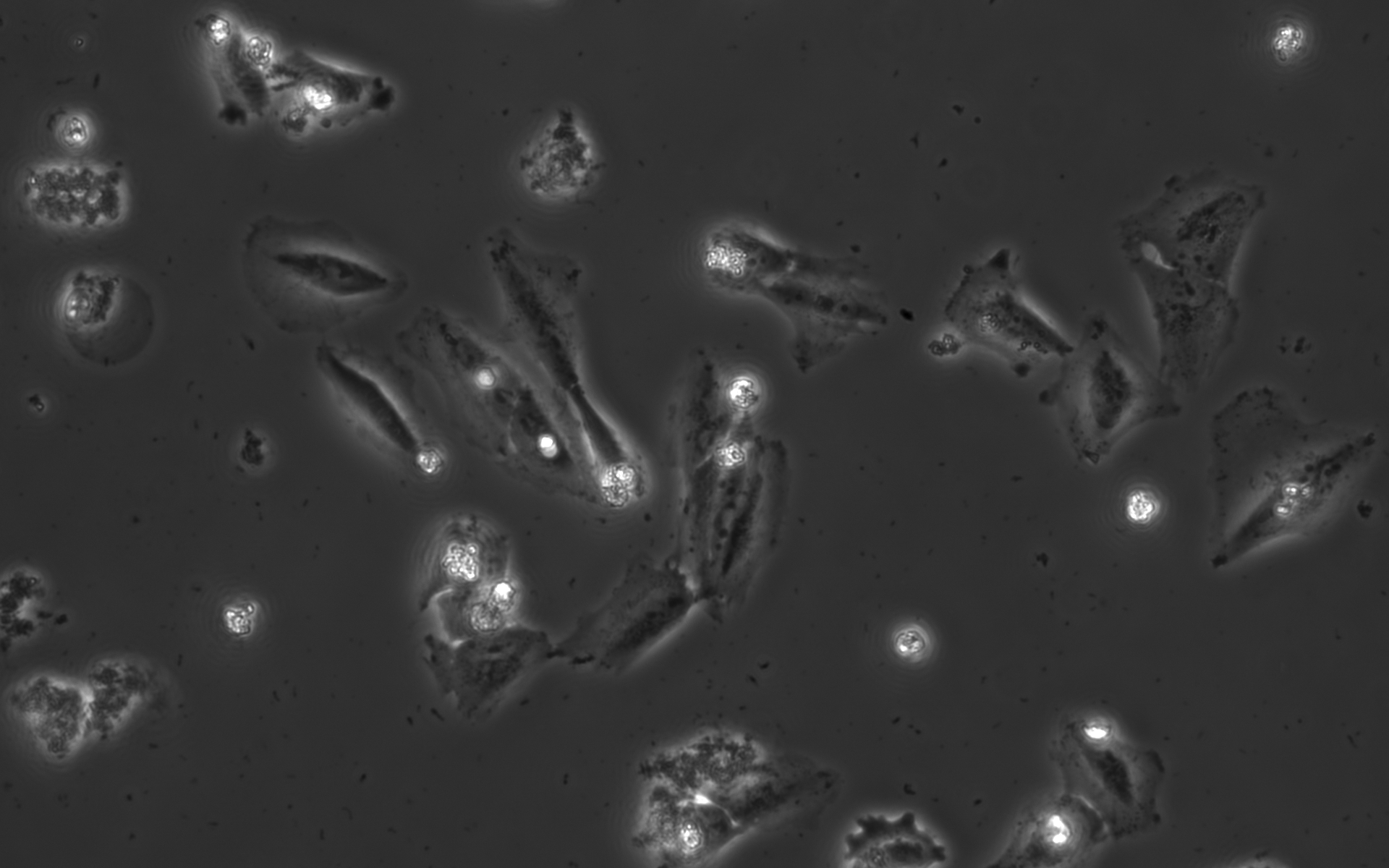

Supplement: Supplementary file 4 — Source data Fig. 3 [file 44318_2024_130_MOESM4_ESM.zip › Figure 3/3A/differentiated HL60/bf (rgb).tif]

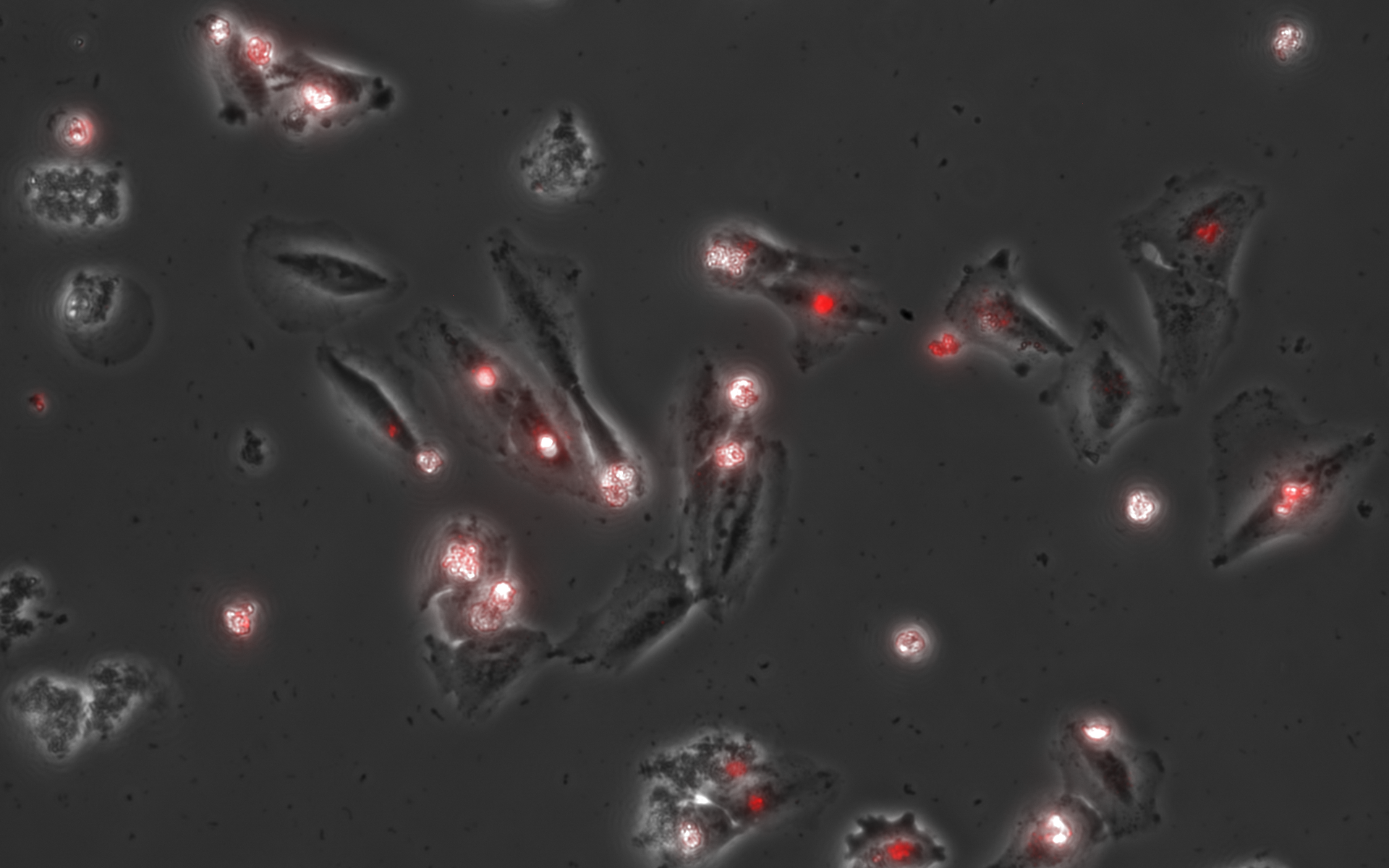

Supplement: Supplementary file 4 — Source data Fig. 3 [file 44318_2024_130_MOESM4_ESM.zip › Figure 3/3A/differentiated HL60/composite (rgb).tif]

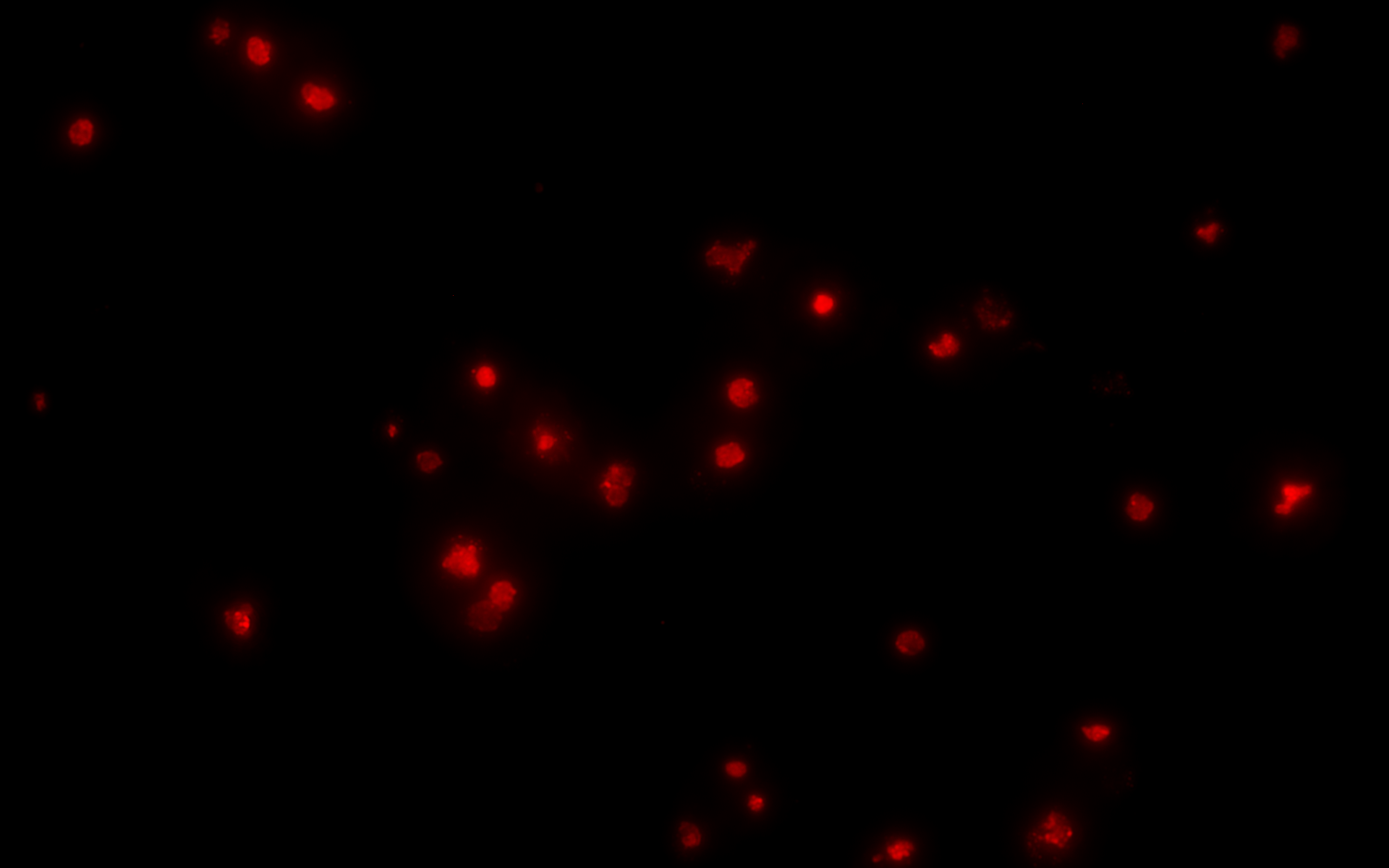

Supplement: Supplementary file 4 — Source data Fig. 3 [file 44318_2024_130_MOESM4_ESM.zip › Figure 3/3A/differentiated HL60/PKH26 (rgb)(1).tif]

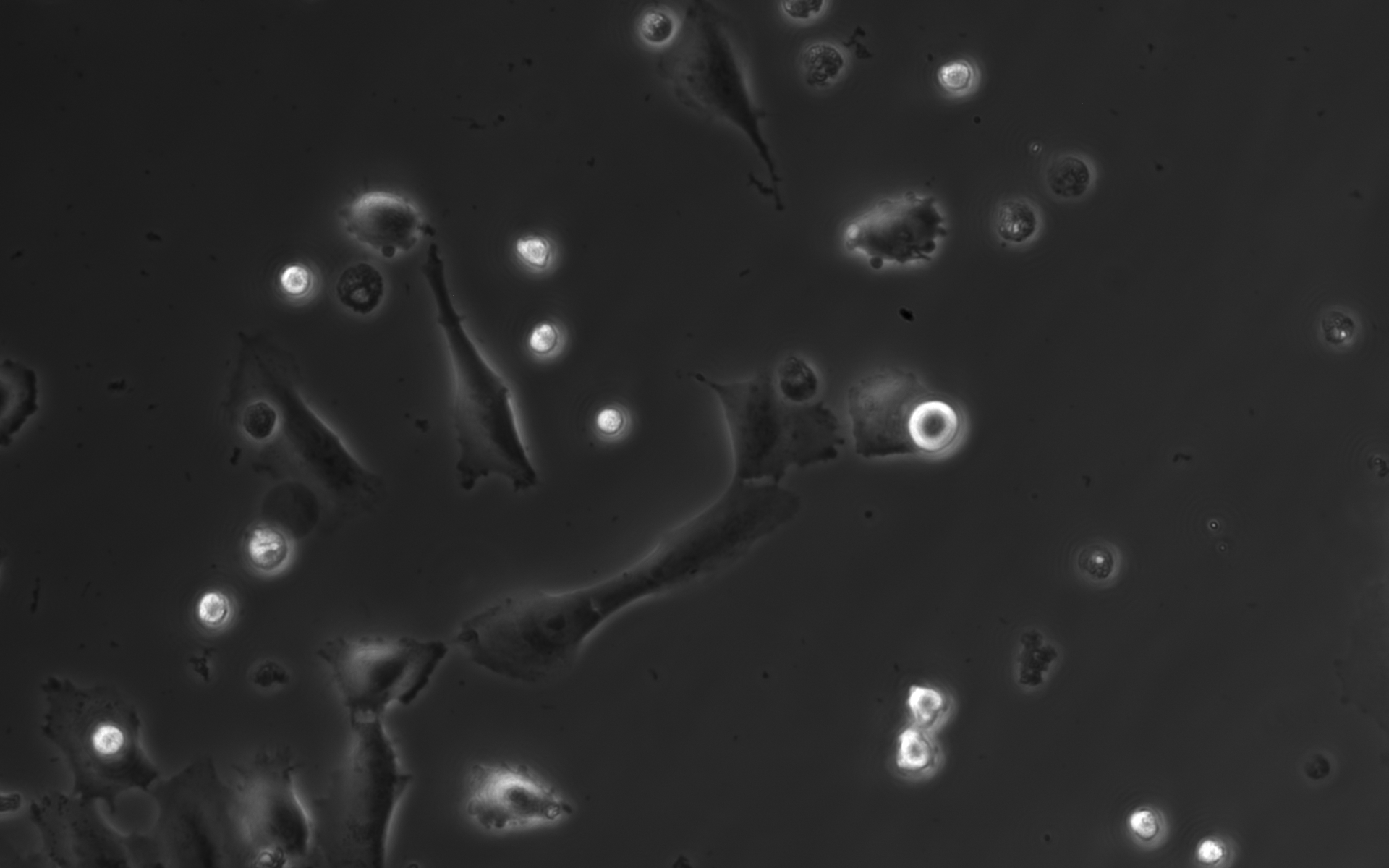

Supplement: Supplementary file 4 — Source data Fig. 3 [file 44318_2024_130_MOESM4_ESM.zip › Figure 3/3A/non-differentiated HL60/bf (rgb).tif]

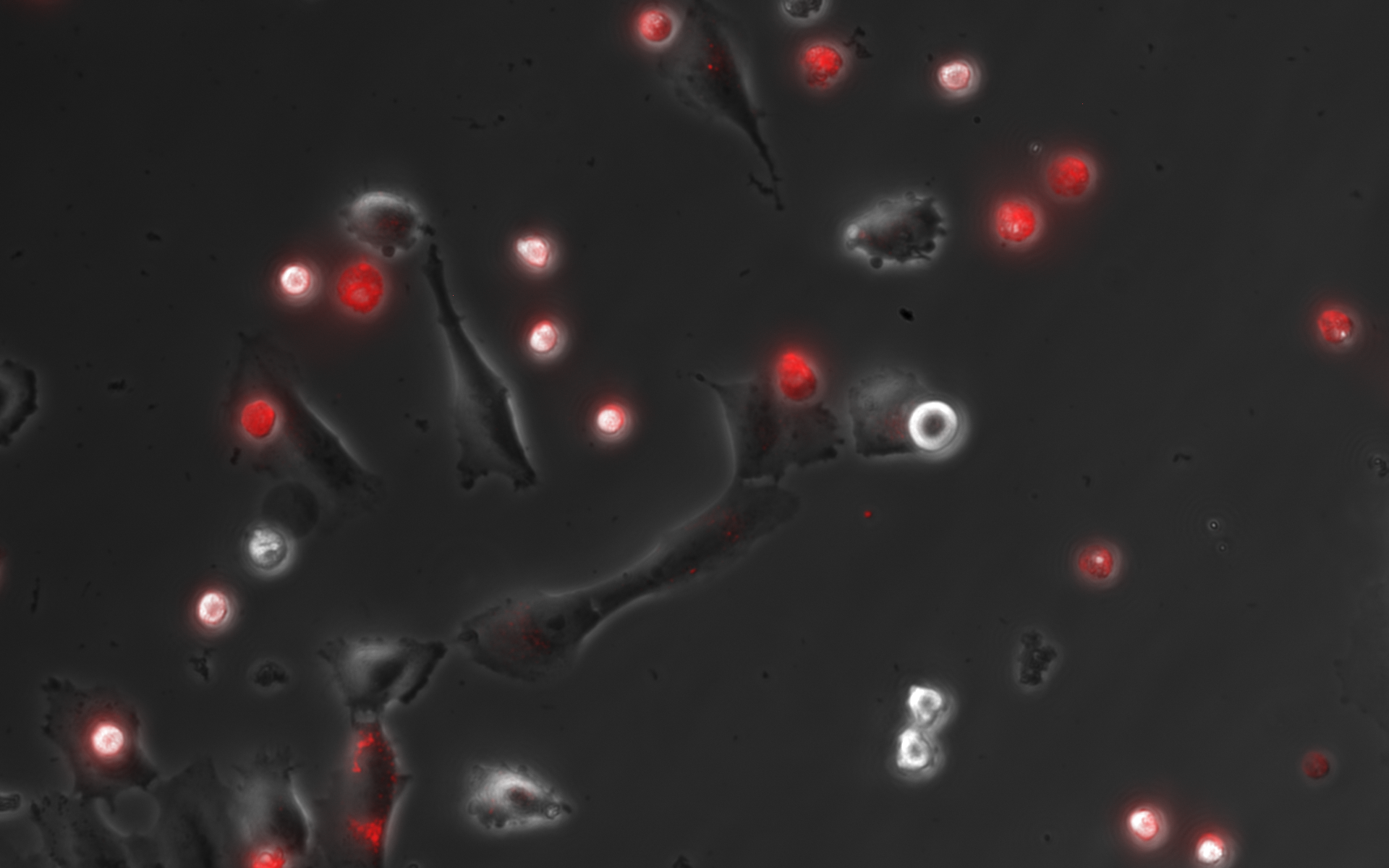

Supplement: Supplementary file 4 — Source data Fig. 3 [file 44318_2024_130_MOESM4_ESM.zip › Figure 3/3A/non-differentiated HL60/composite (rgb).tif]

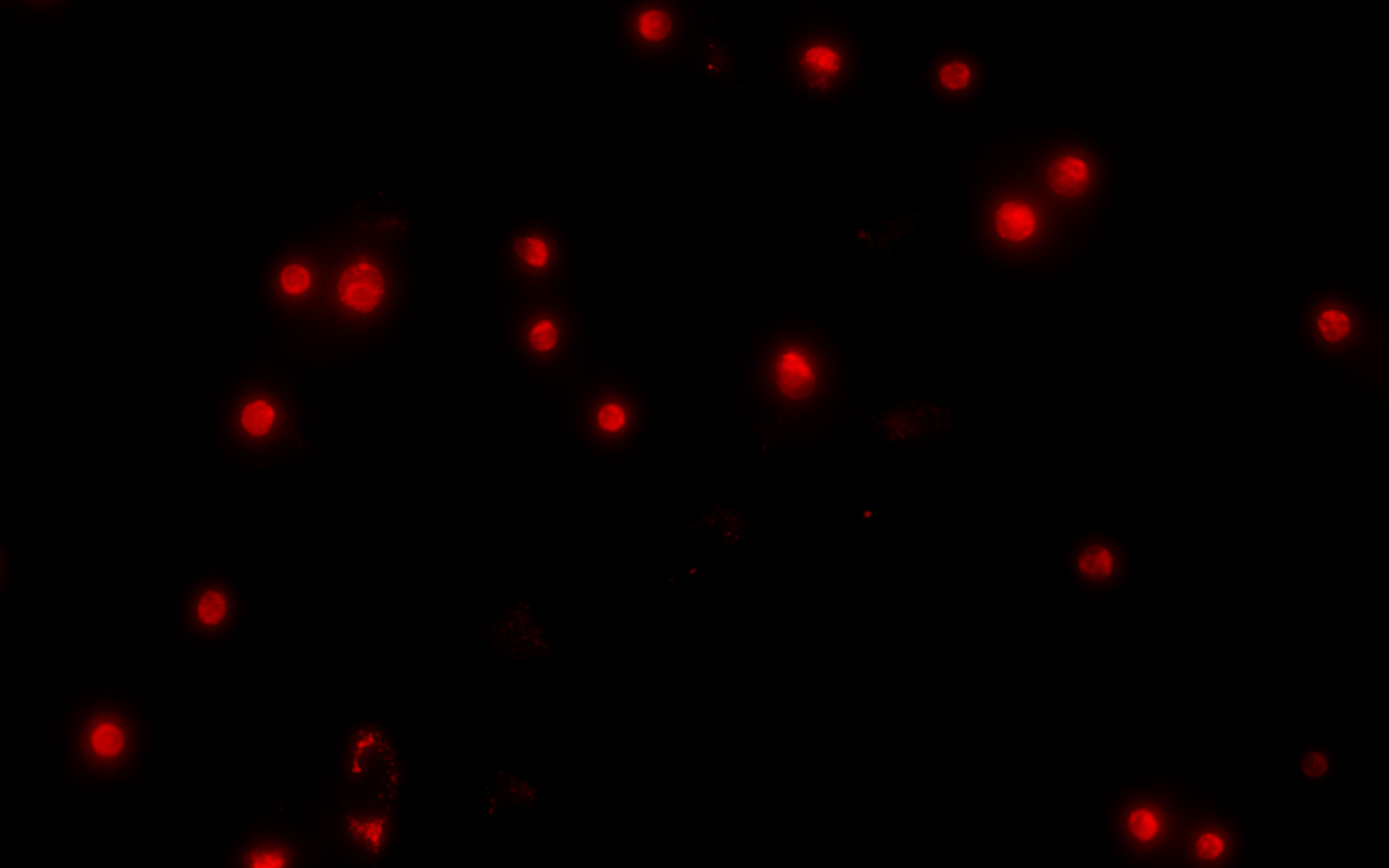

Supplement: Supplementary file 4 — Source data Fig. 3 [file 44318_2024_130_MOESM4_ESM.zip › Figure 3/3A/non-differentiated HL60/PKH26 (rgb).tif]

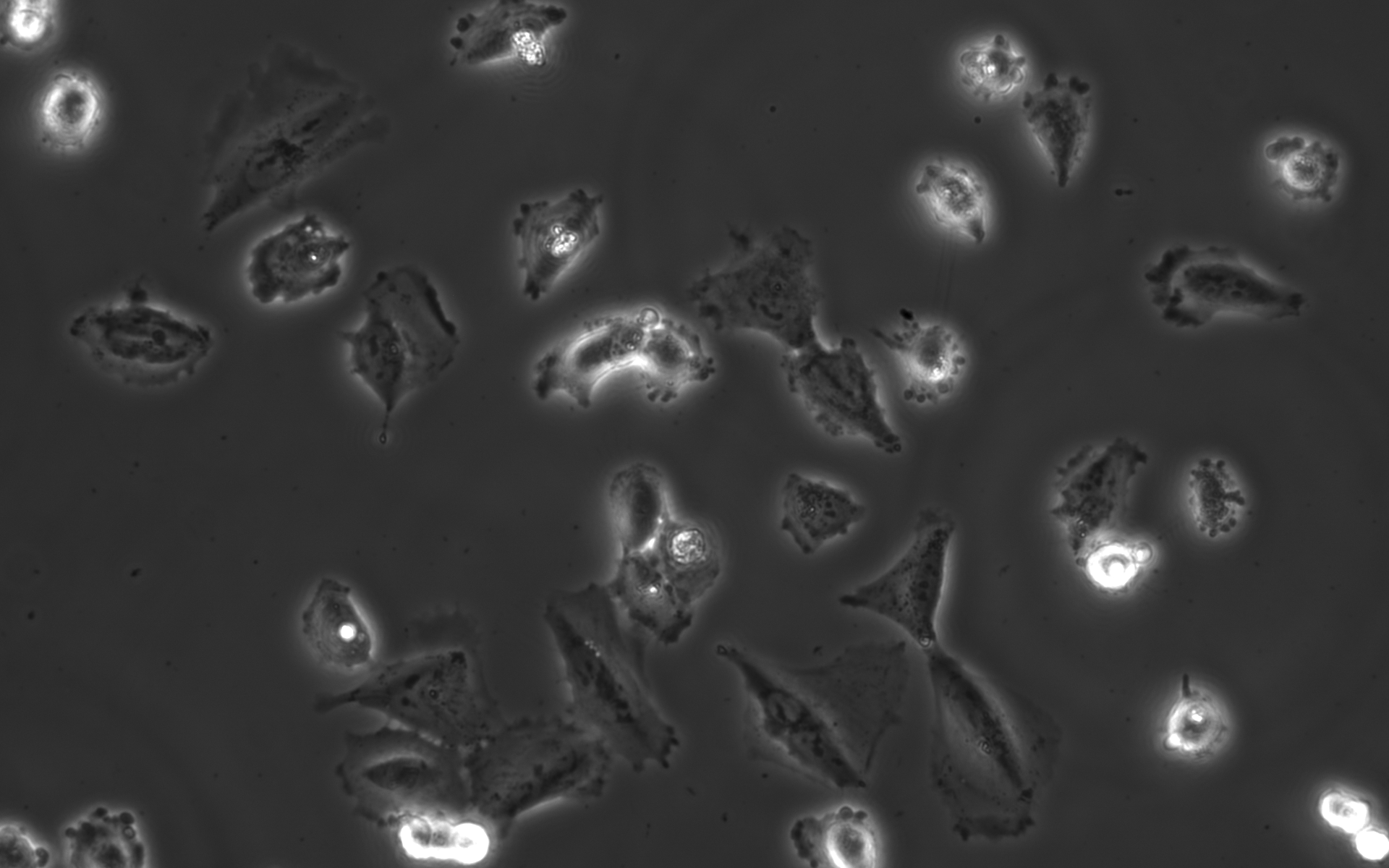

Supplement: Supplementary file 4 — Source data Fig. 3 [file 44318_2024_130_MOESM4_ESM.zip › Figure 3/3C/differentiated HL60/bf (rgb).tif]

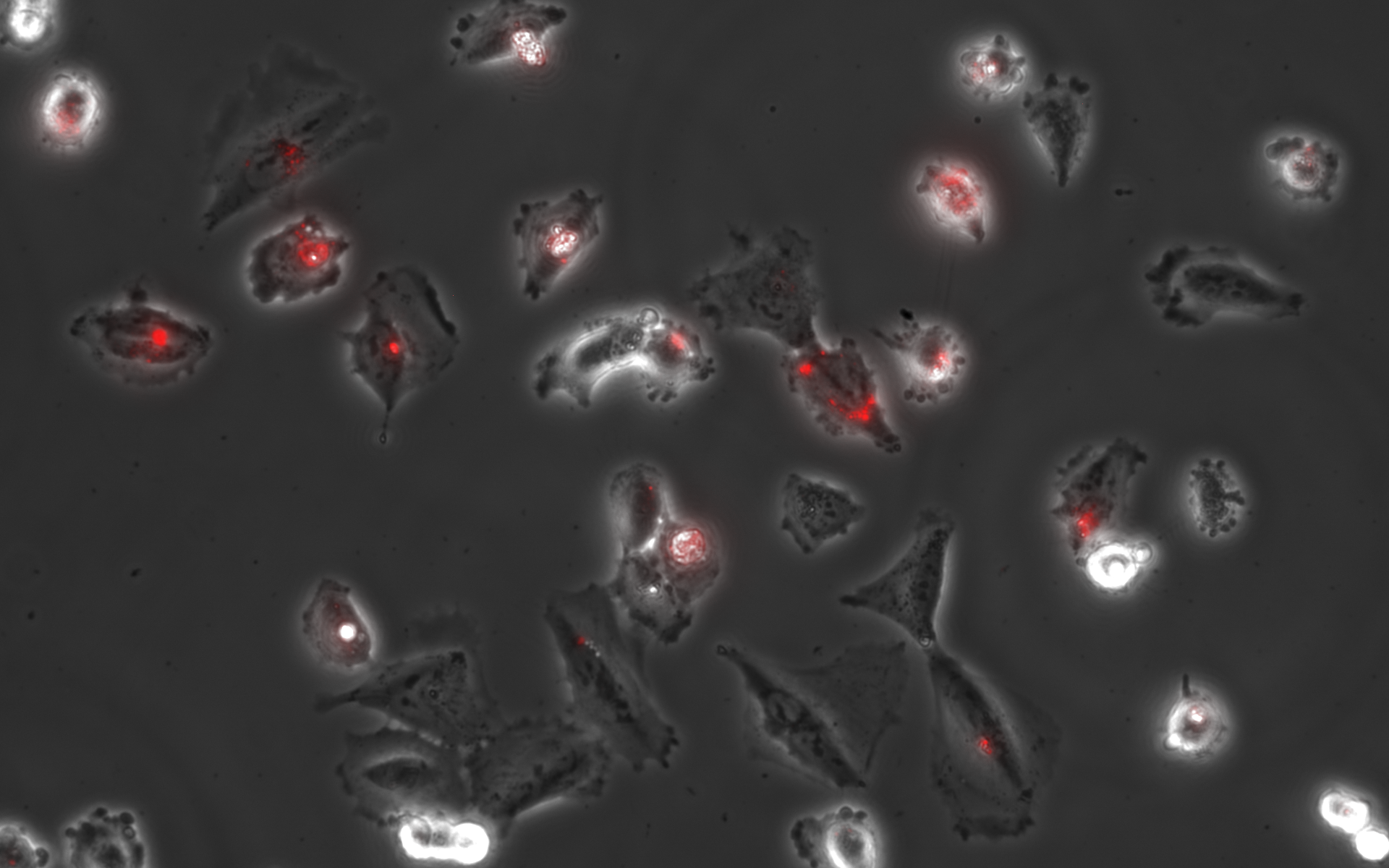

Supplement: Supplementary file 4 — Source data Fig. 3 [file 44318_2024_130_MOESM4_ESM.zip › Figure 3/3C/differentiated HL60/composite (rgb).tif]

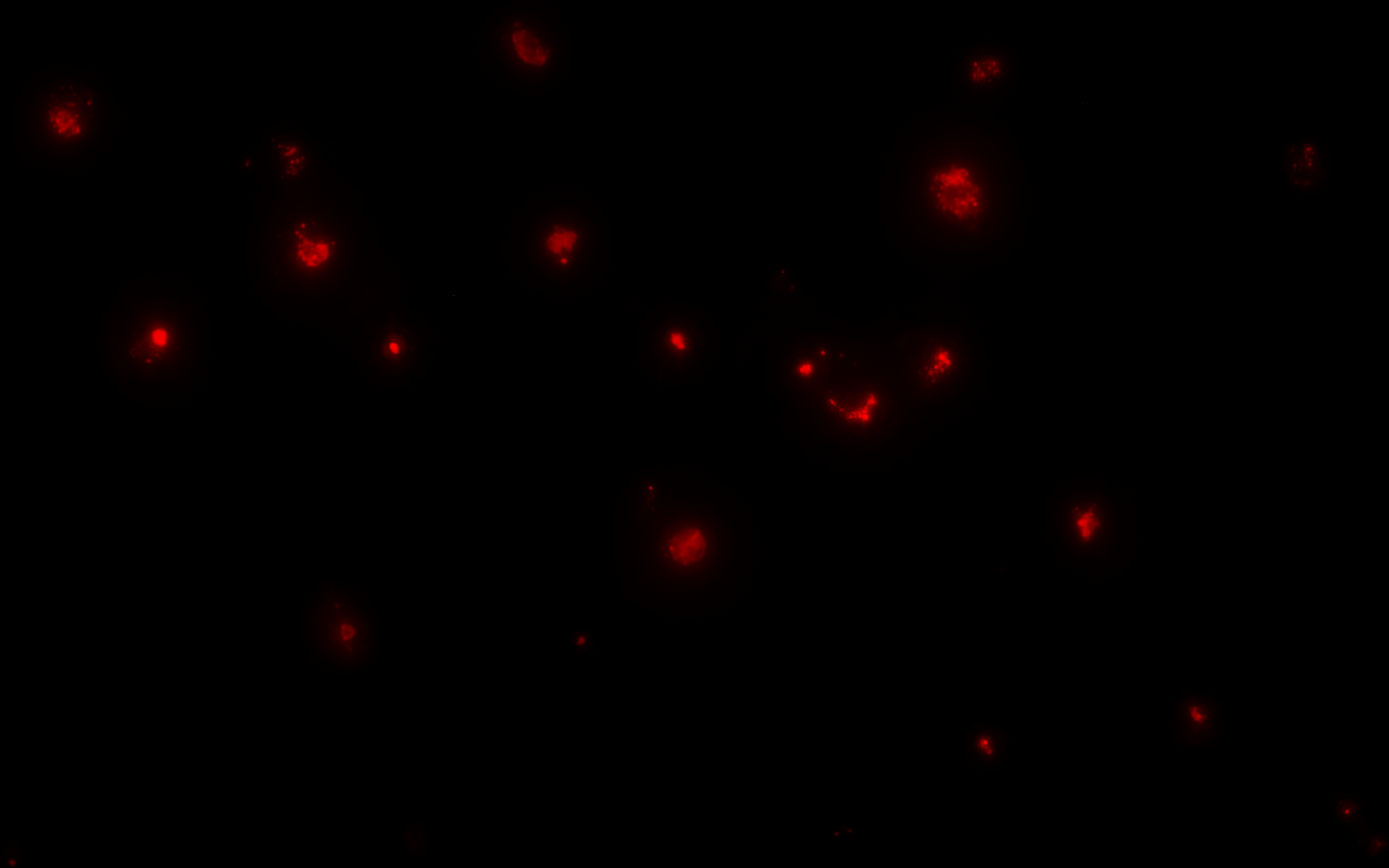

Supplement: Supplementary file 4 — Source data Fig. 3 [file 44318_2024_130_MOESM4_ESM.zip › Figure 3/3C/differentiated HL60/PKH26 (rgb).tif]

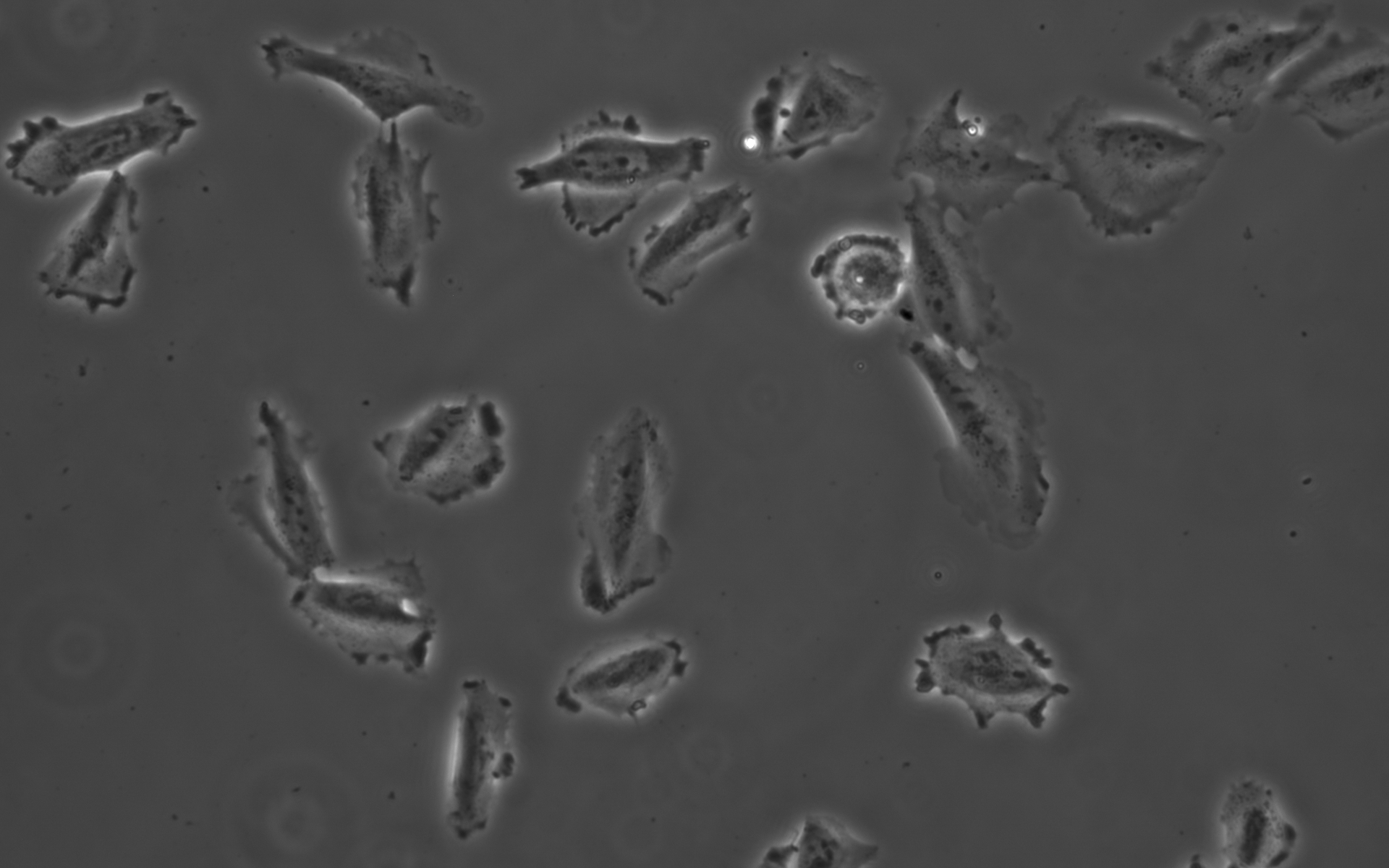

Supplement: Supplementary file 4 — Source data Fig. 3 [file 44318_2024_130_MOESM4_ESM.zip › Figure 3/3C/non-differentiated HL60/bf (rgb).tif]

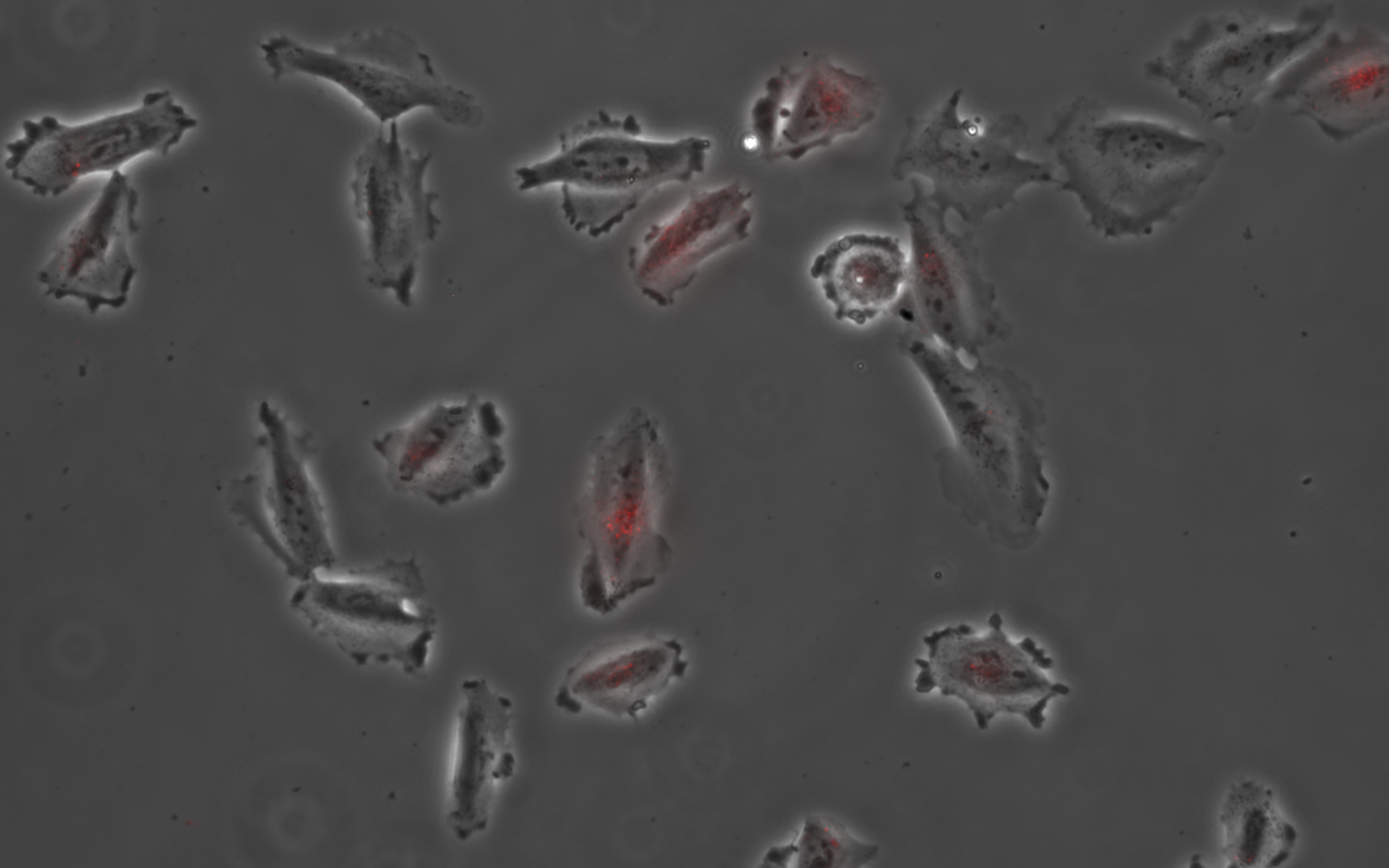

Supplement: Supplementary file 4 — Source data Fig. 3 [file 44318_2024_130_MOESM4_ESM.zip › Figure 3/3C/non-differentiated HL60/composite (rgb).tif]

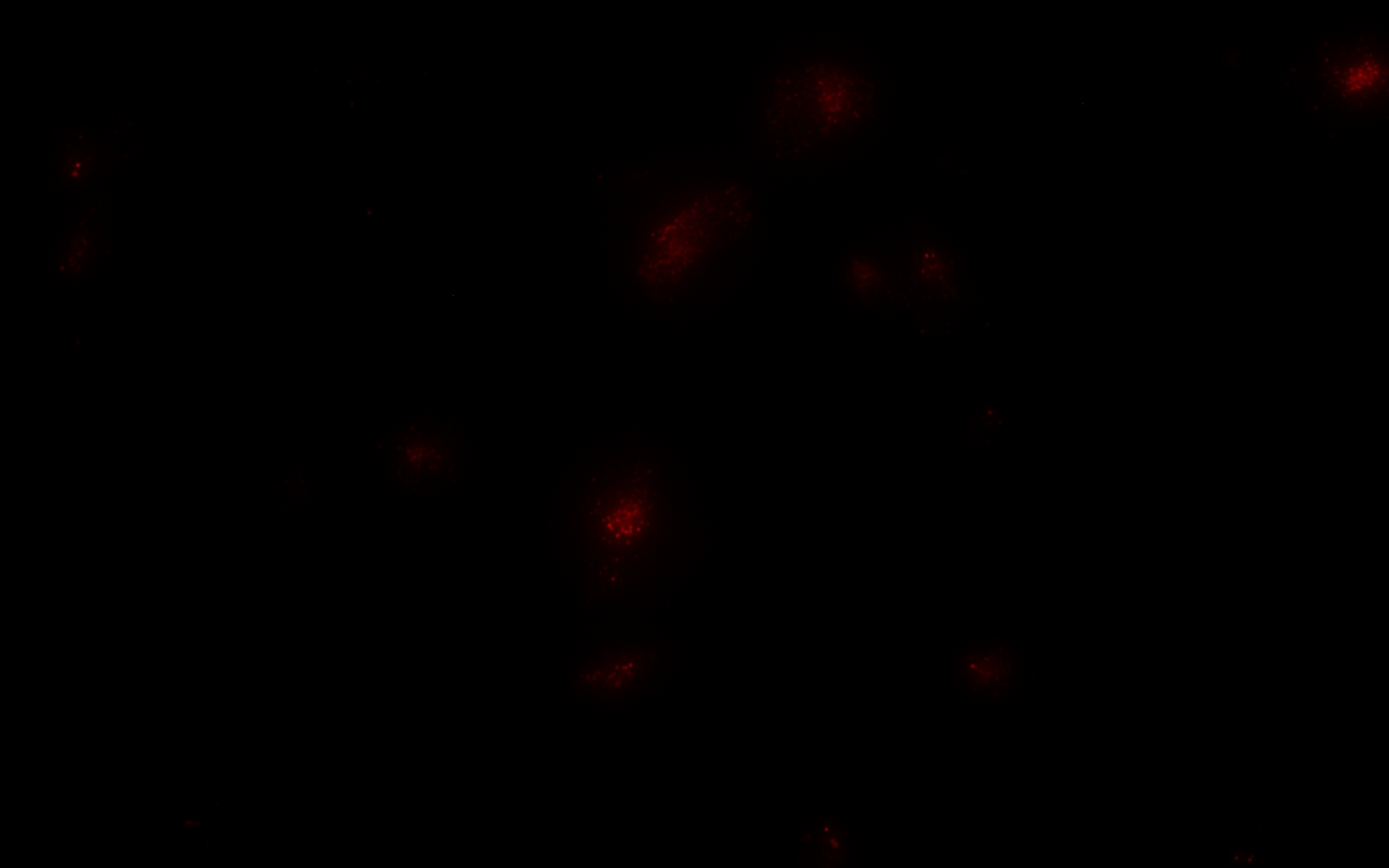

Supplement: Supplementary file 4 — Source data Fig. 3 [file 44318_2024_130_MOESM4_ESM.zip › Figure 3/3C/non-differentiated HL60/PKH26 (rgb).tif]

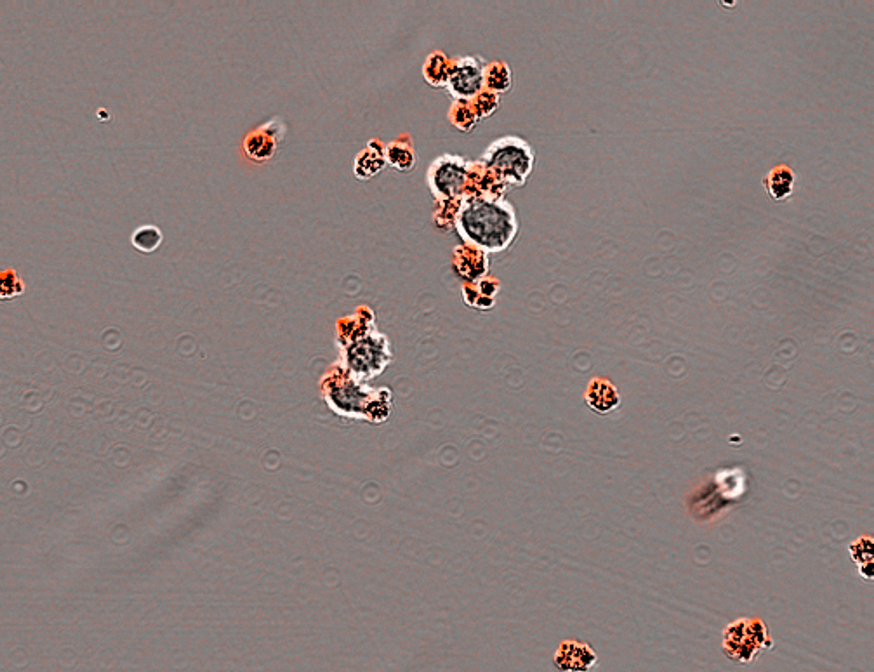

Supplement: Supplementary file 4 — Source data Fig. 3 [file 44318_2024_130_MOESM4_ESM.zip › Figure 3/3F/LN229-4SA PKH26-HL60 1 mM RGDS coculture.tif]

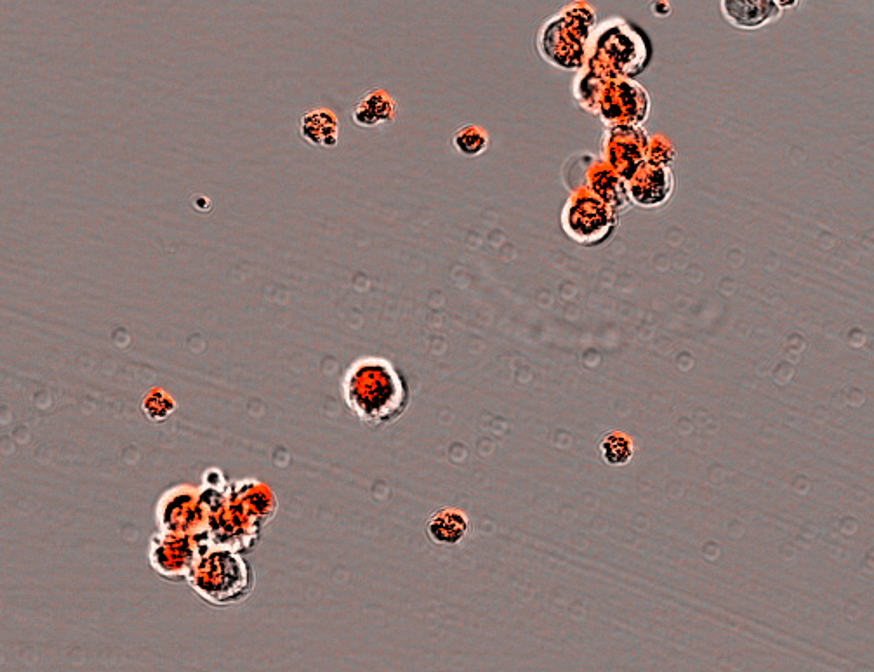

Supplement: Supplementary file 4 — Source data Fig. 3 [file 44318_2024_130_MOESM4_ESM.zip › Figure 3/3F/LN229-4SA PKH26-HL60 pbs coculture.tif]

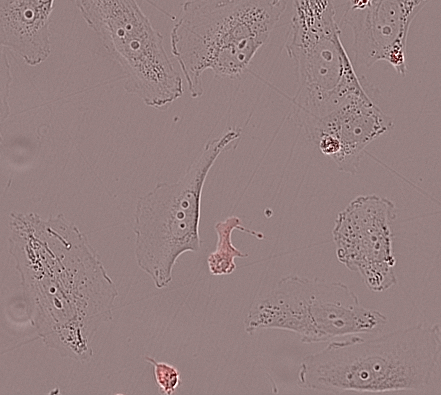

Supplement: Supplementary file 5 — Source data Fig. 4 [file 44318_2024_130_MOESM5_ESM.zip › Figure 4/4A/4A.tif]

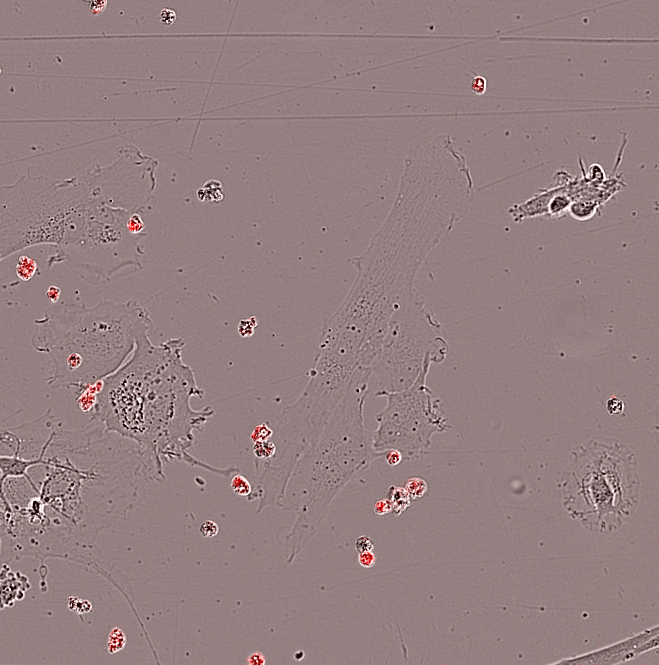

Supplement: Supplementary file 5 — Source data Fig. 4 [file 44318_2024_130_MOESM5_ESM.zip › Figure 4/4B/4B.tif]

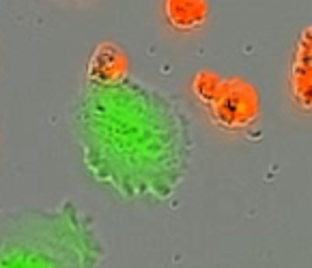

Supplement: Supplementary file 5 — Source data Fig. 4 [file 44318_2024_130_MOESM5_ESM.zip › Figure 4/4C/0 min.tif]

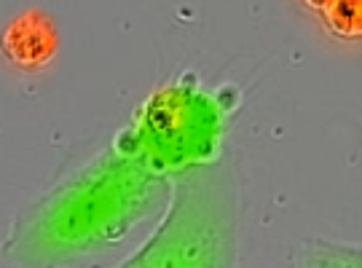

Supplement: Supplementary file 5 — Source data Fig. 4 [file 44318_2024_130_MOESM5_ESM.zip › Figure 4/4C/180 min.tif]

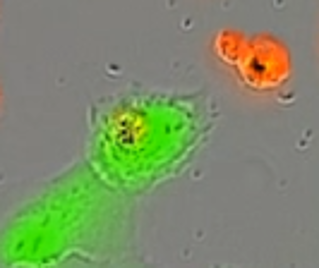

Supplement: Supplementary file 5 — Source data Fig. 4 [file 44318_2024_130_MOESM5_ESM.zip › Figure 4/4C/60 min.tif]

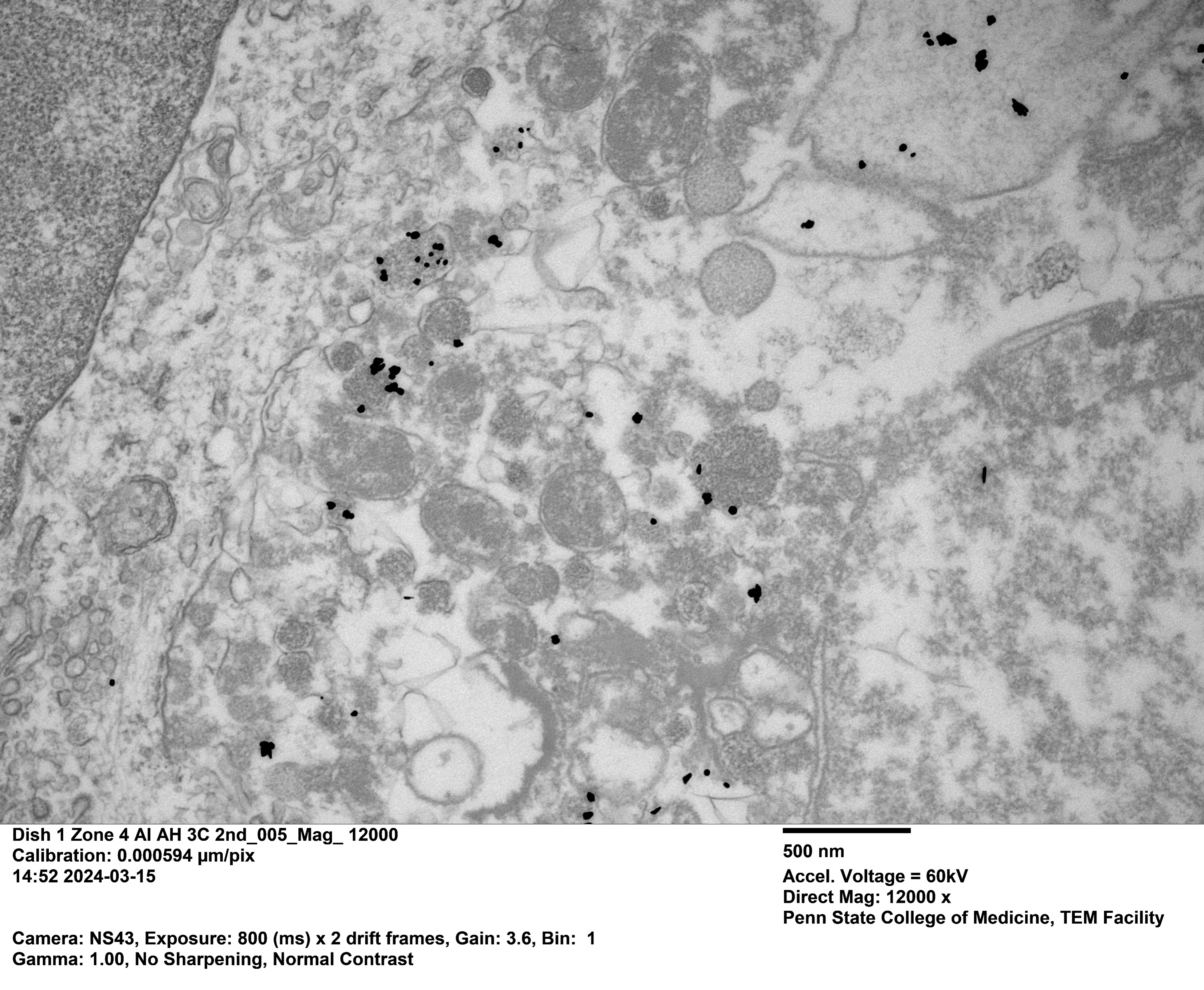

Supplement: Supplementary file 5 — Source data Fig. 4 [file 44318_2024_130_MOESM5_ESM.zip › Figure 4/4G/12000x.tif]

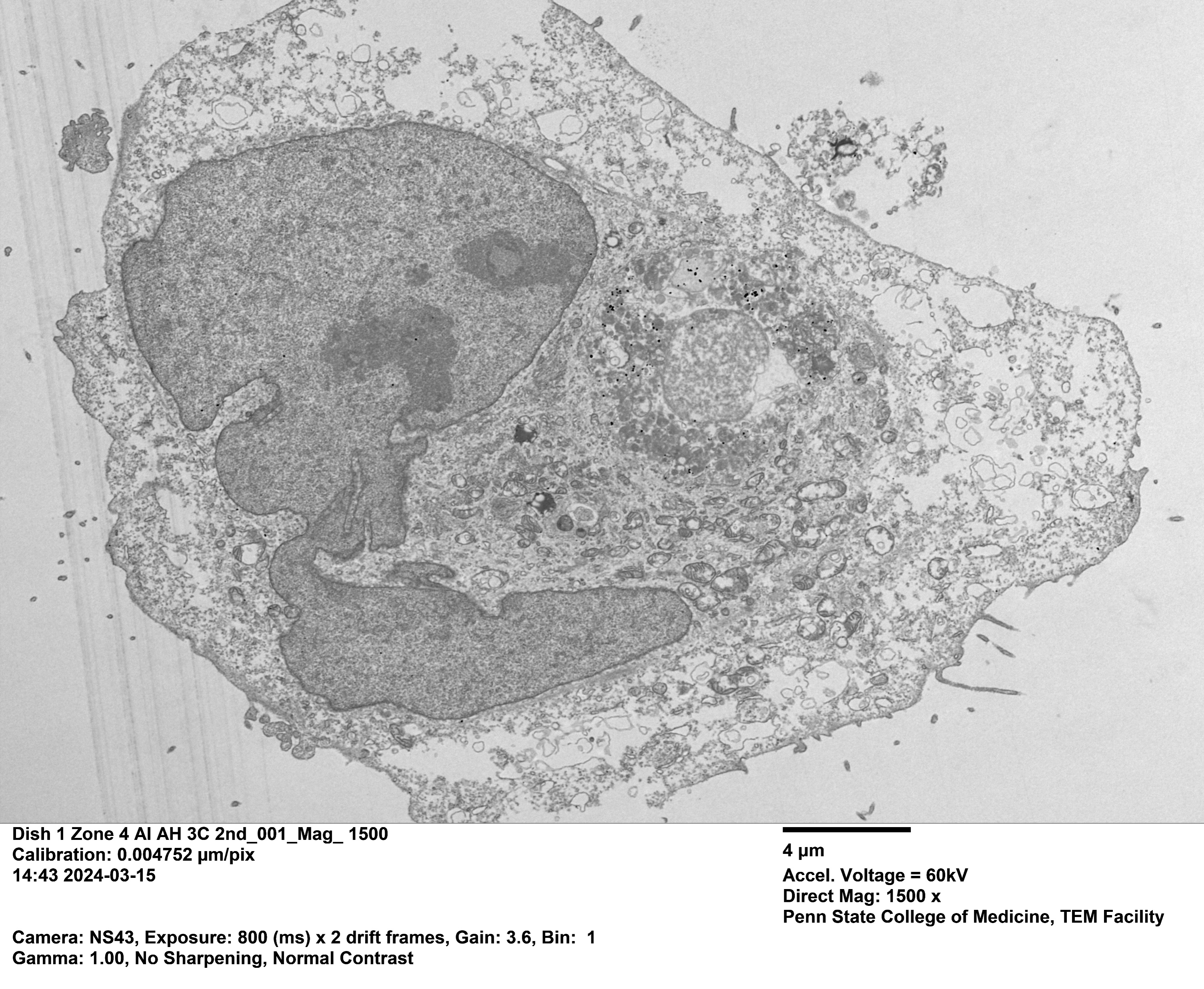

Supplement: Supplementary file 5 — Source data Fig. 4 [file 44318_2024_130_MOESM5_ESM.zip › Figure 4/4G/1500x.tif]

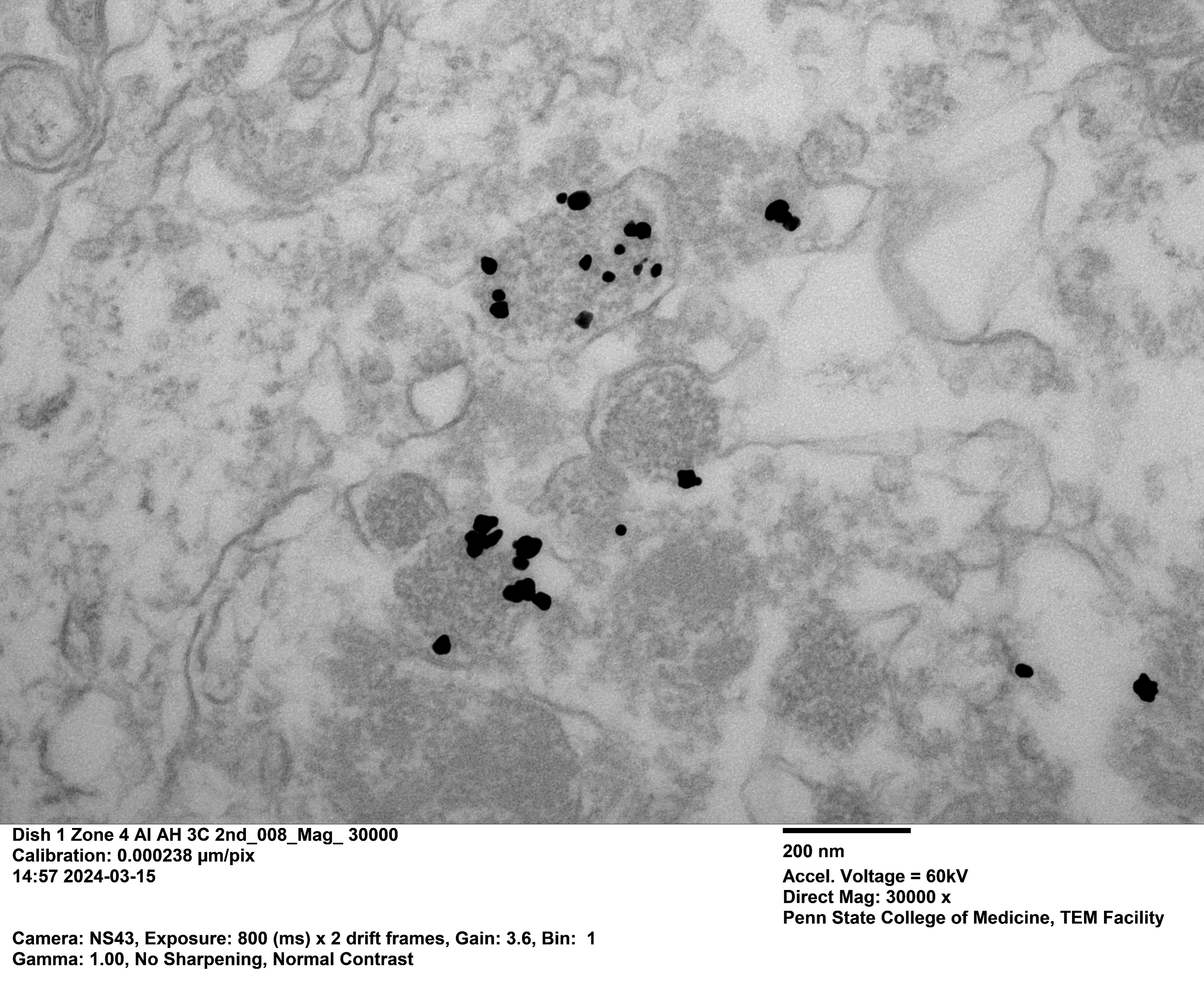

Supplement: Supplementary file 5 — Source data Fig. 4 [file 44318_2024_130_MOESM5_ESM.zip › Figure 4/4G/30000x.tif]

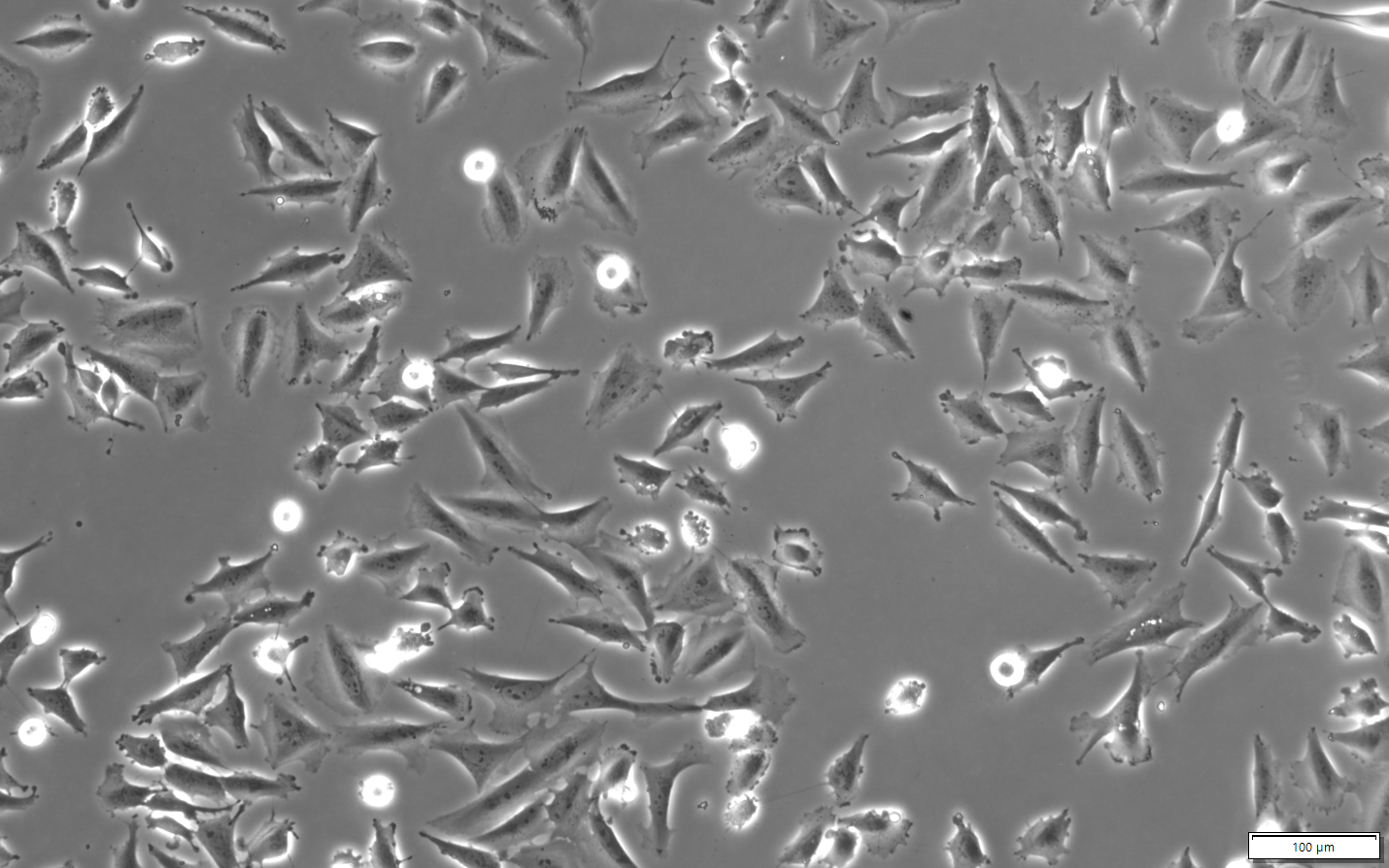

Supplement: Supplementary file 6 — Source data Fig. 5 [file 44318_2024_130_MOESM6_ESM.zip › Figure 5/5A/DMSO.tif]

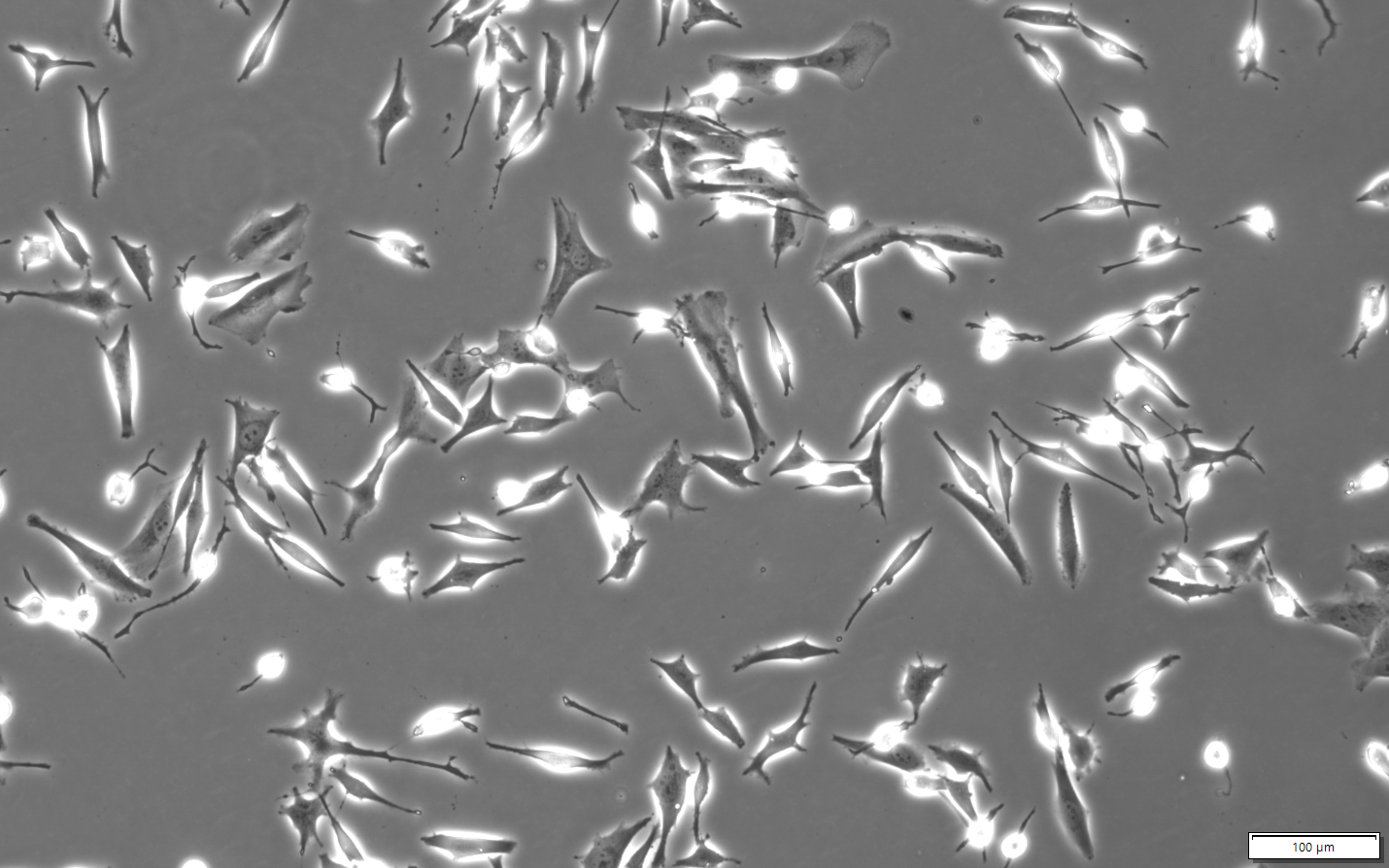

Supplement: Supplementary file 6 — Source data Fig. 5 [file 44318_2024_130_MOESM6_ESM.zip › Figure 5/5A/Fluvastatin.tif]

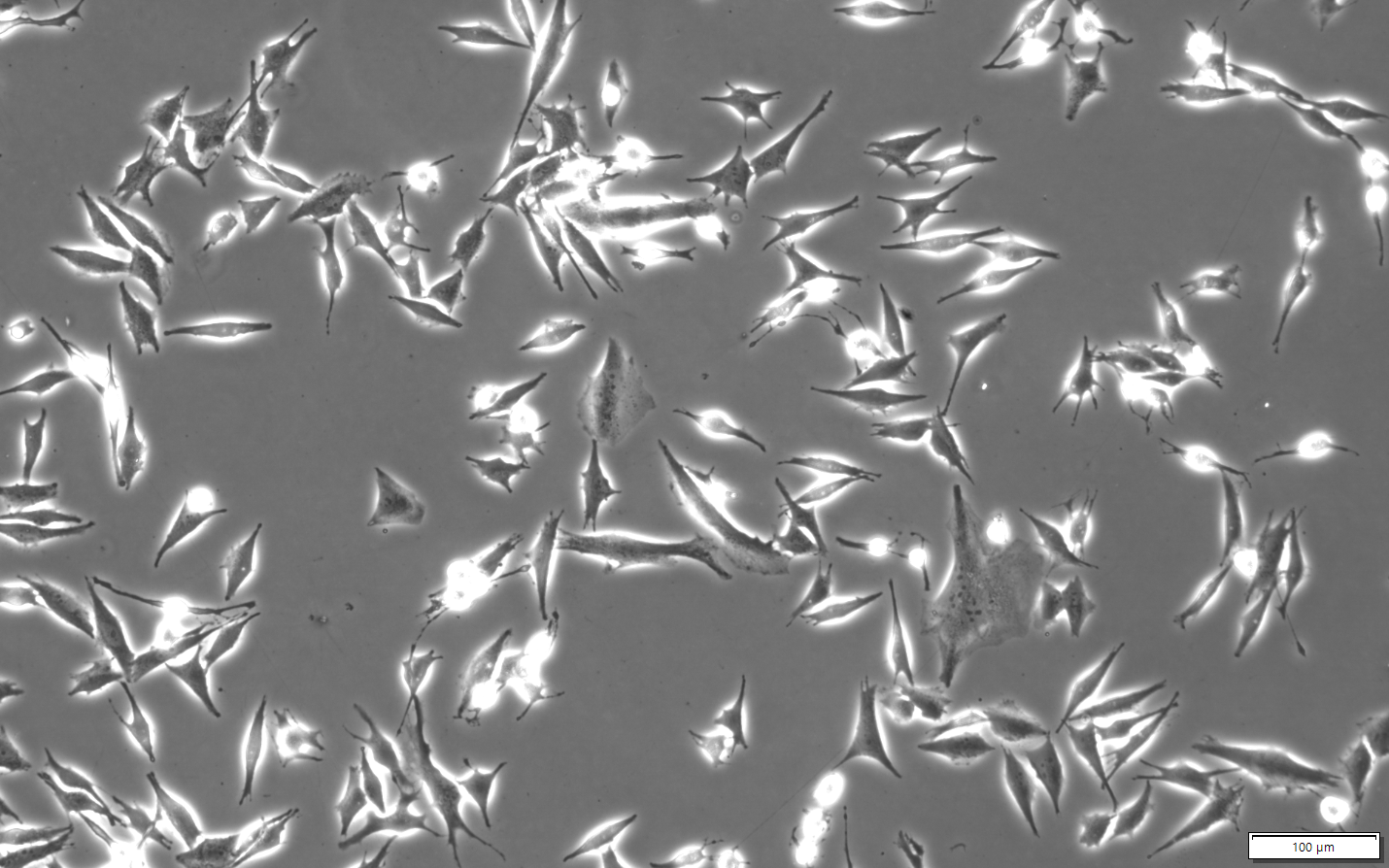

Supplement: Supplementary file 6 — Source data Fig. 5 [file 44318_2024_130_MOESM6_ESM.zip › Figure 5/5A/GGTI-298.tif]

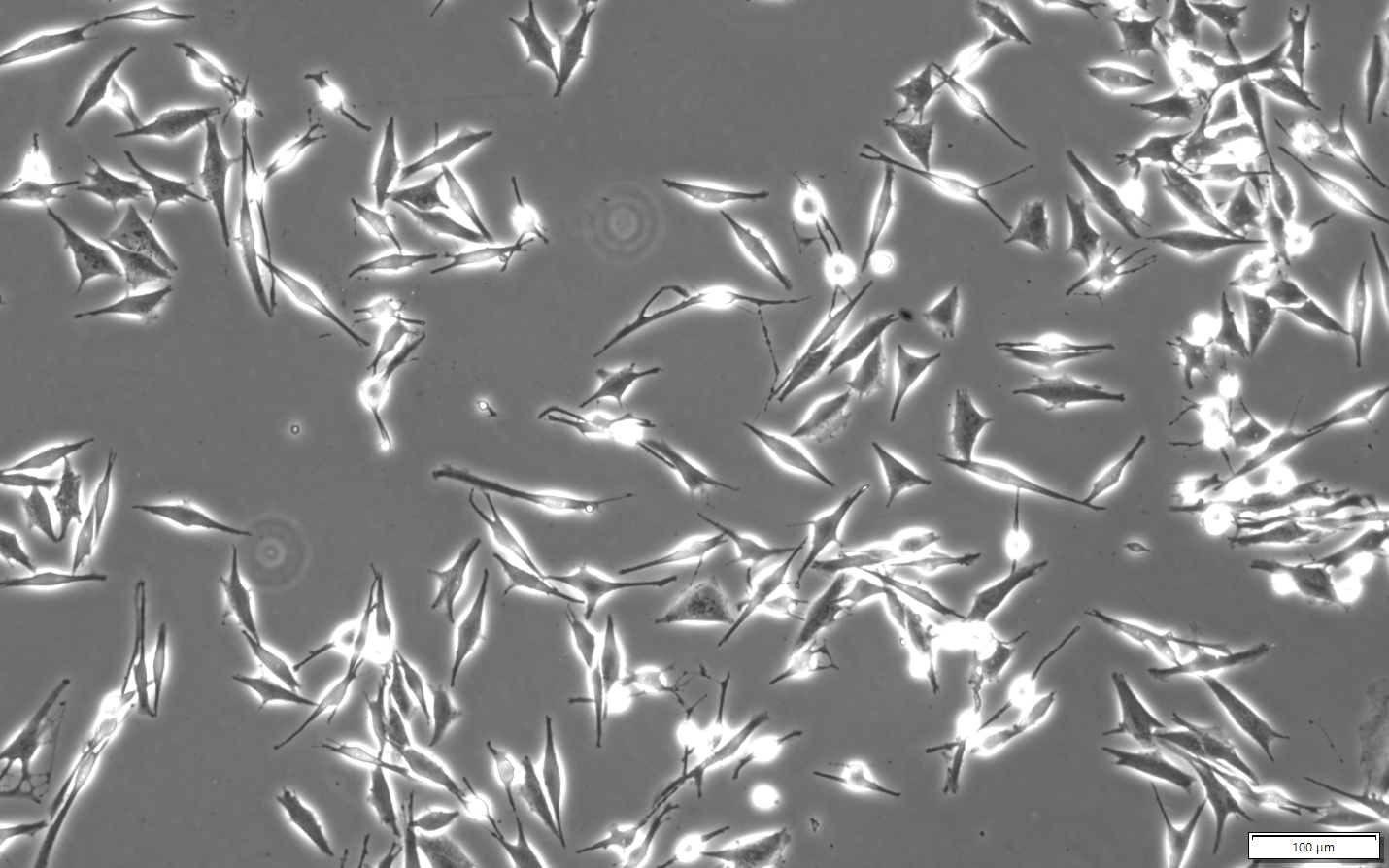

Supplement: Supplementary file 6 — Source data Fig. 5 [file 44318_2024_130_MOESM6_ESM.zip › Figure 5/5A/Simvastatin.tif]

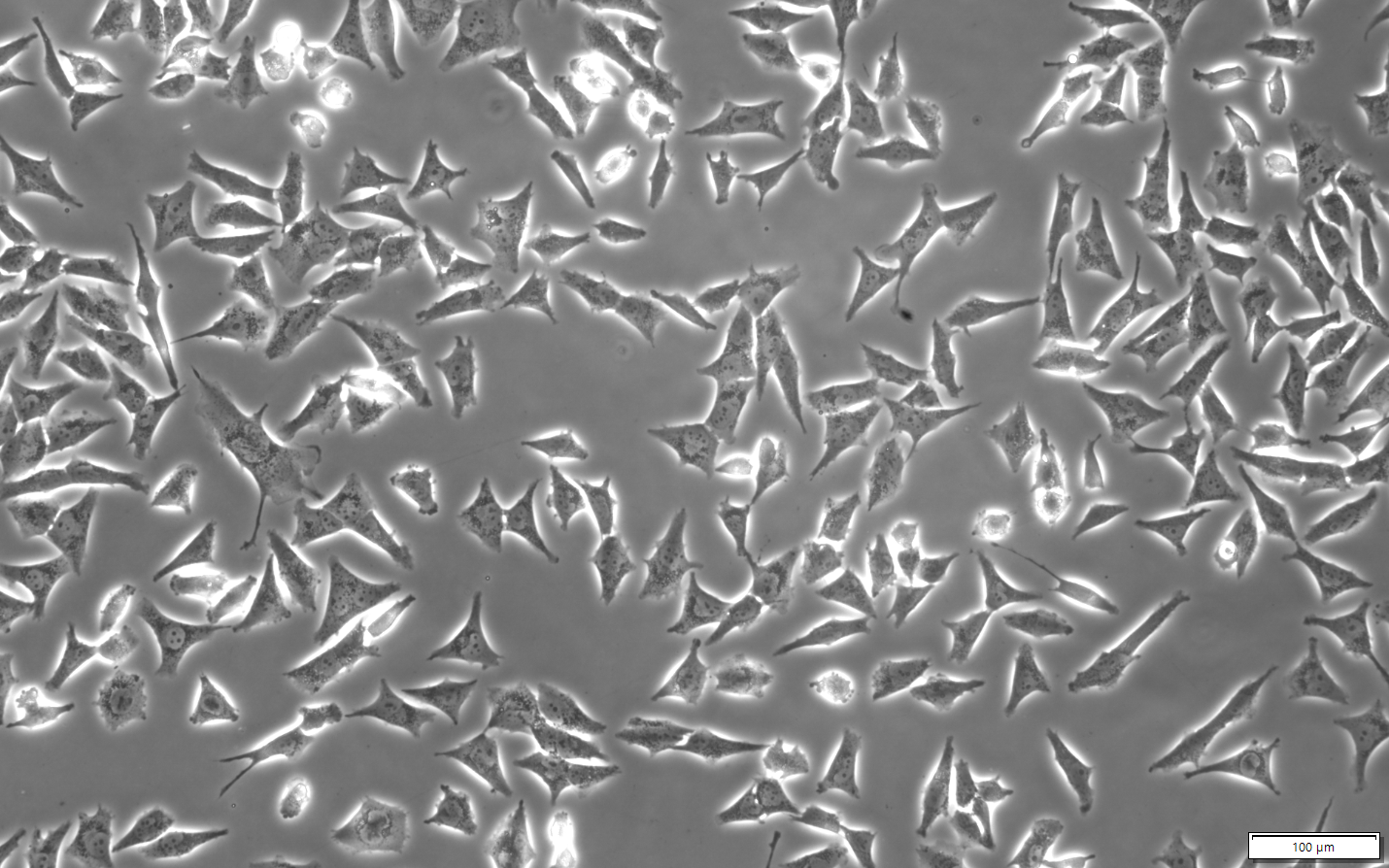

Supplement: Supplementary file 6 — Source data Fig. 5 [file 44318_2024_130_MOESM6_ESM.zip › Figure 5/5A/YM53601.tif]

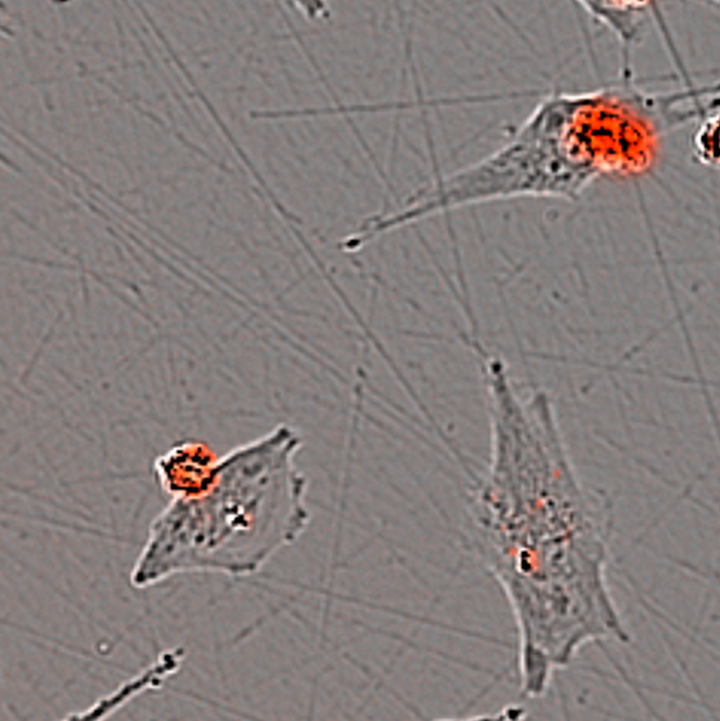

Supplement: Supplementary file 6 — Source data Fig. 5 [file 44318_2024_130_MOESM6_ESM.zip › Figure 5/5D/Attached.tif]

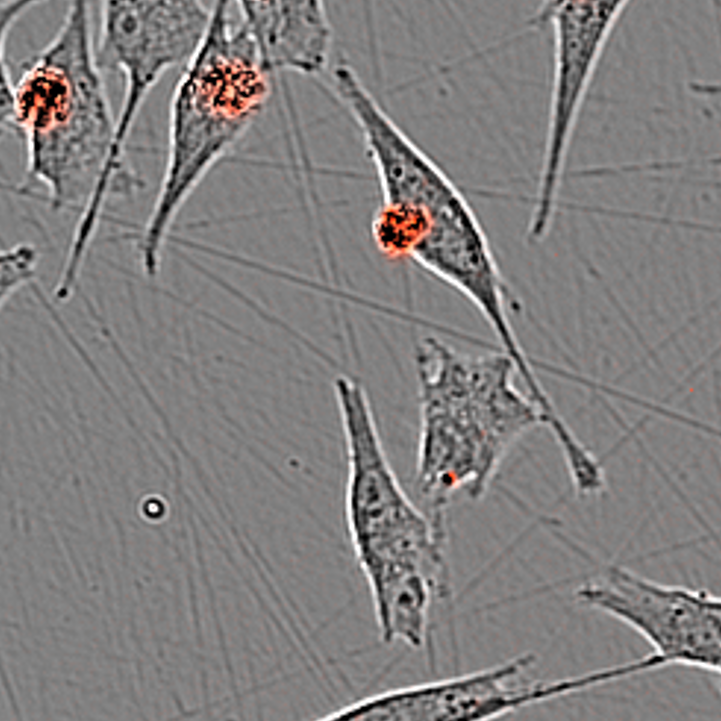

Supplement: Supplementary file 6 — Source data Fig. 5 [file 44318_2024_130_MOESM6_ESM.zip › Figure 5/5D/Entering.tif]

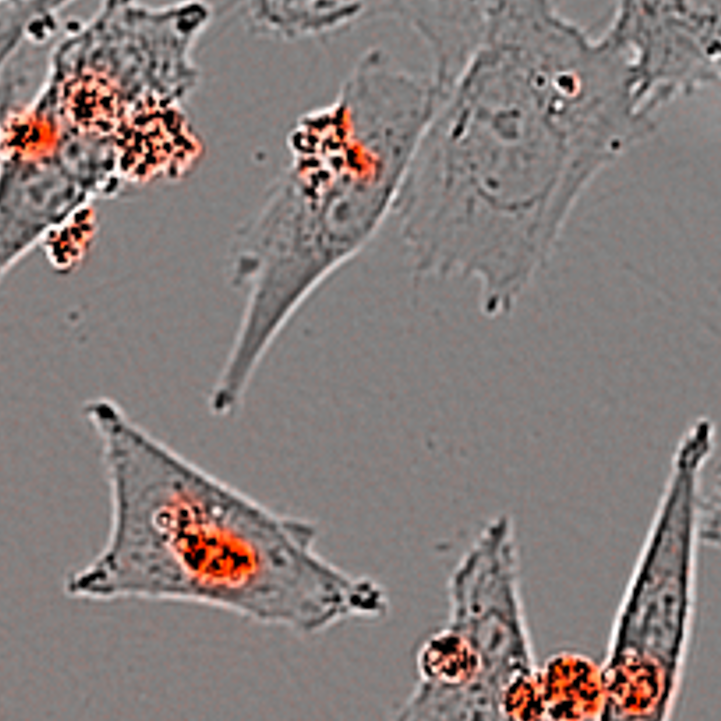

Supplement: Supplementary file 6 — Source data Fig. 5 [file 44318_2024_130_MOESM6_ESM.zip › Figure 5/5D/Internalized.tif]

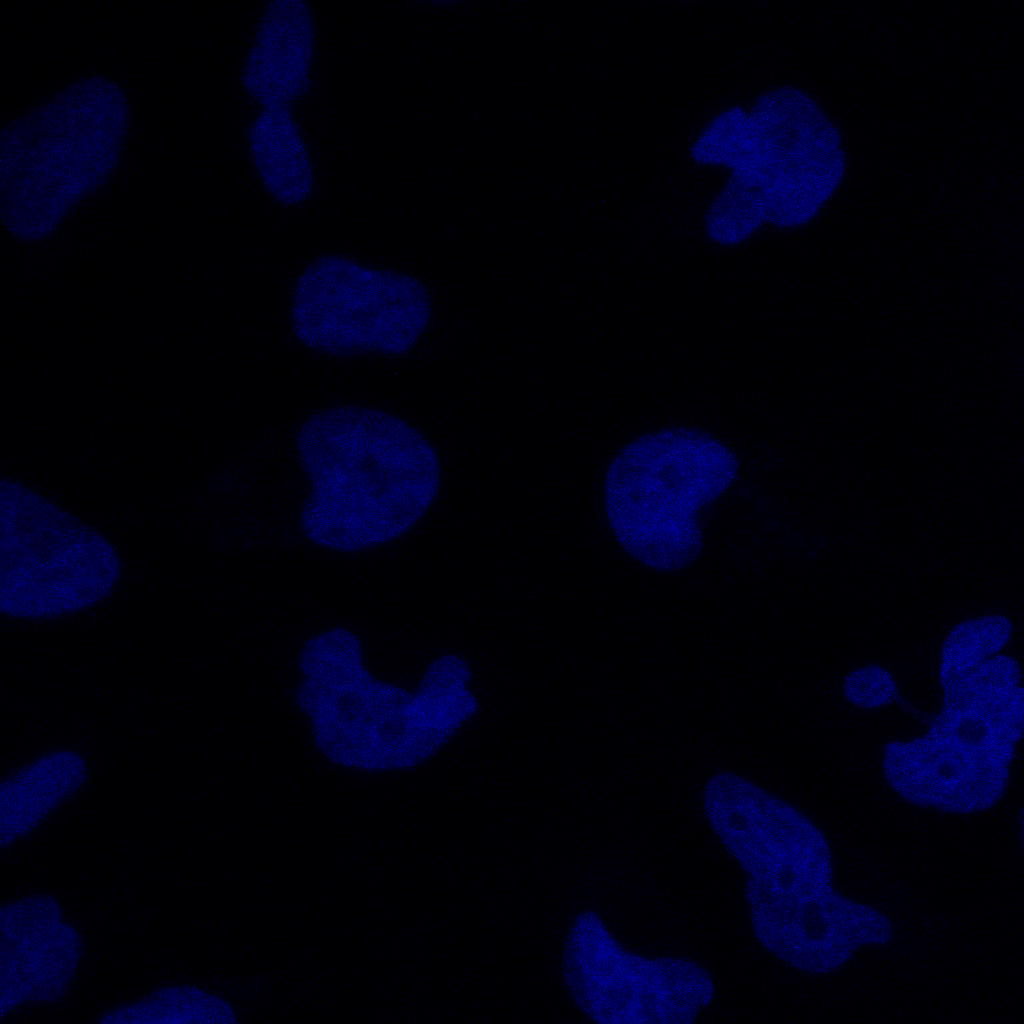

Supplement: Supplementary file 6 — Source data Fig. 5 [file 44318_2024_130_MOESM6_ESM.zip › Figure 5/5F/4SA HL60 coculture DAPI.tif]

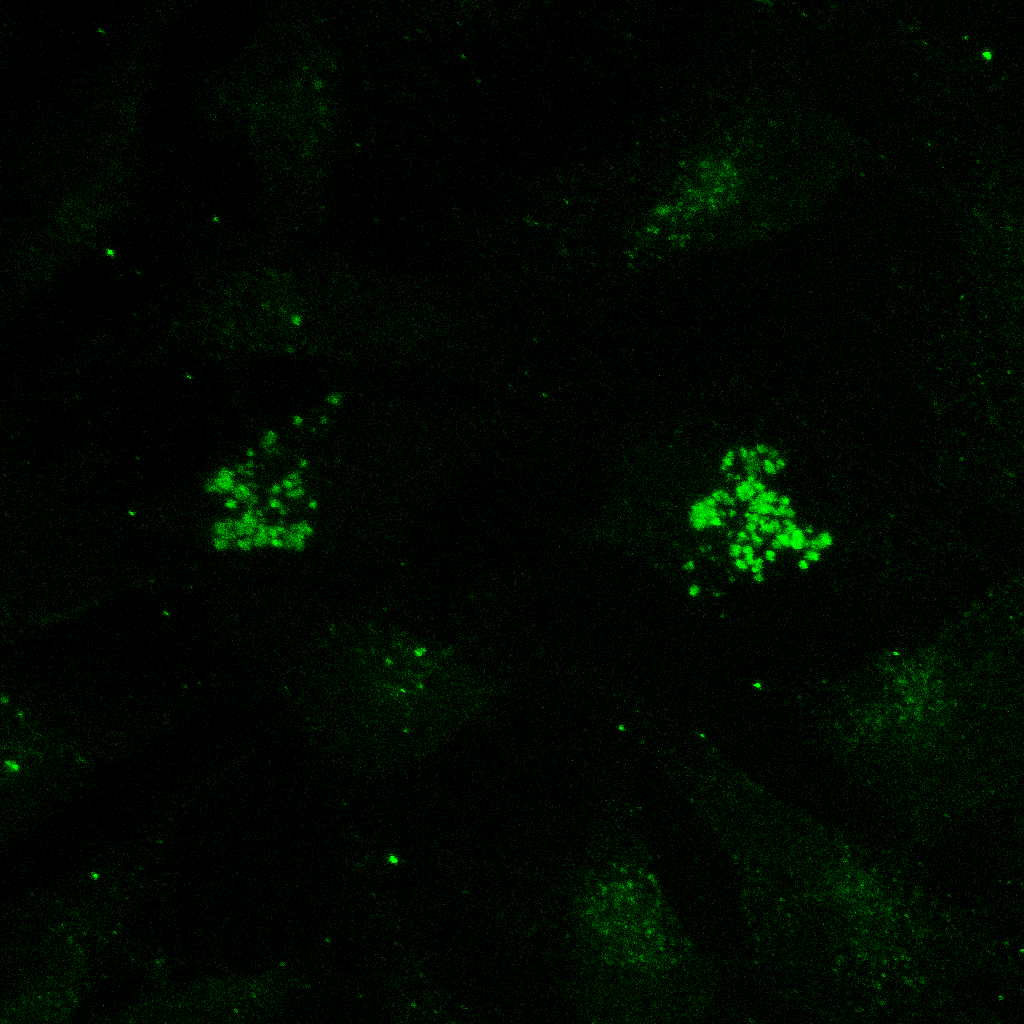

Supplement: Supplementary file 6 — Source data Fig. 5 [file 44318_2024_130_MOESM6_ESM.zip › Figure 5/5F/4SA HL60 coculture LAMP1.tif]

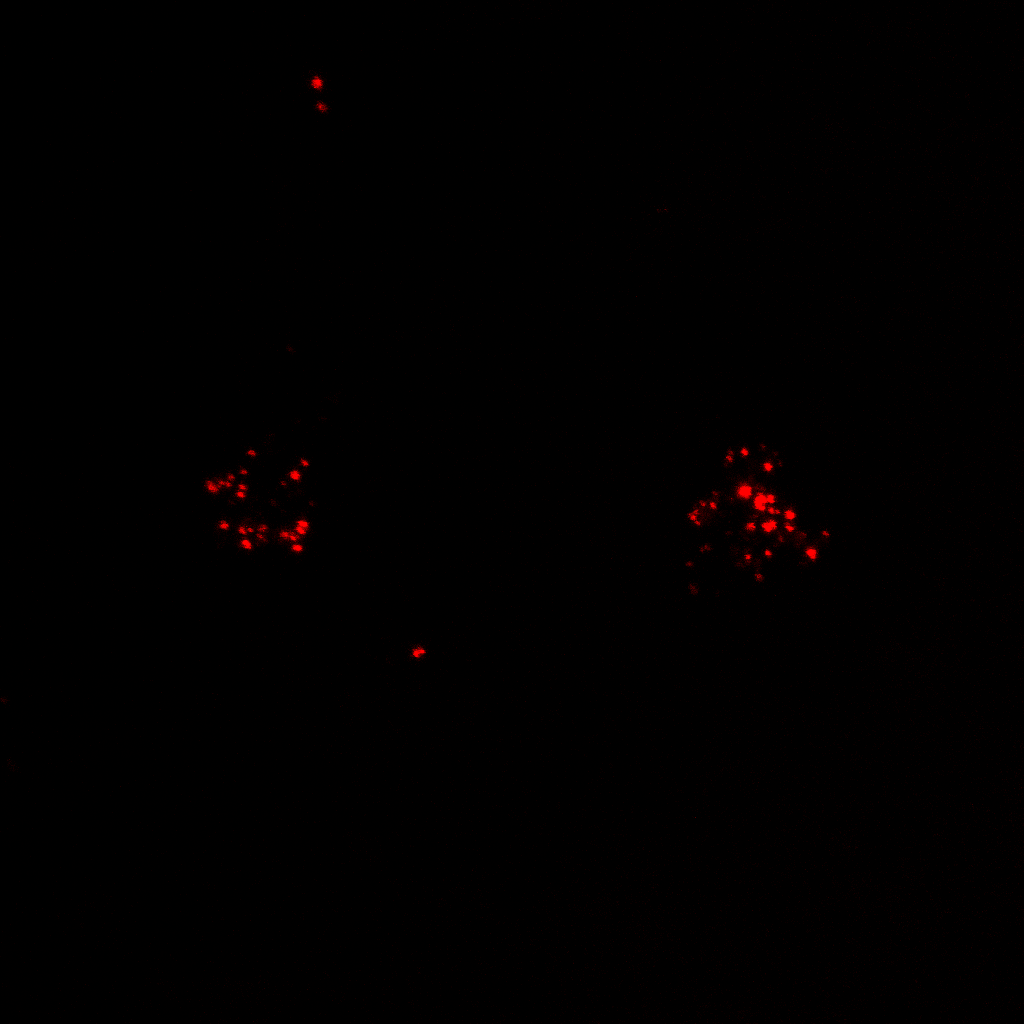

Supplement: Supplementary file 6 — Source data Fig. 5 [file 44318_2024_130_MOESM6_ESM.zip › Figure 5/5F/4SA HL60 coculture PKH26.tif]

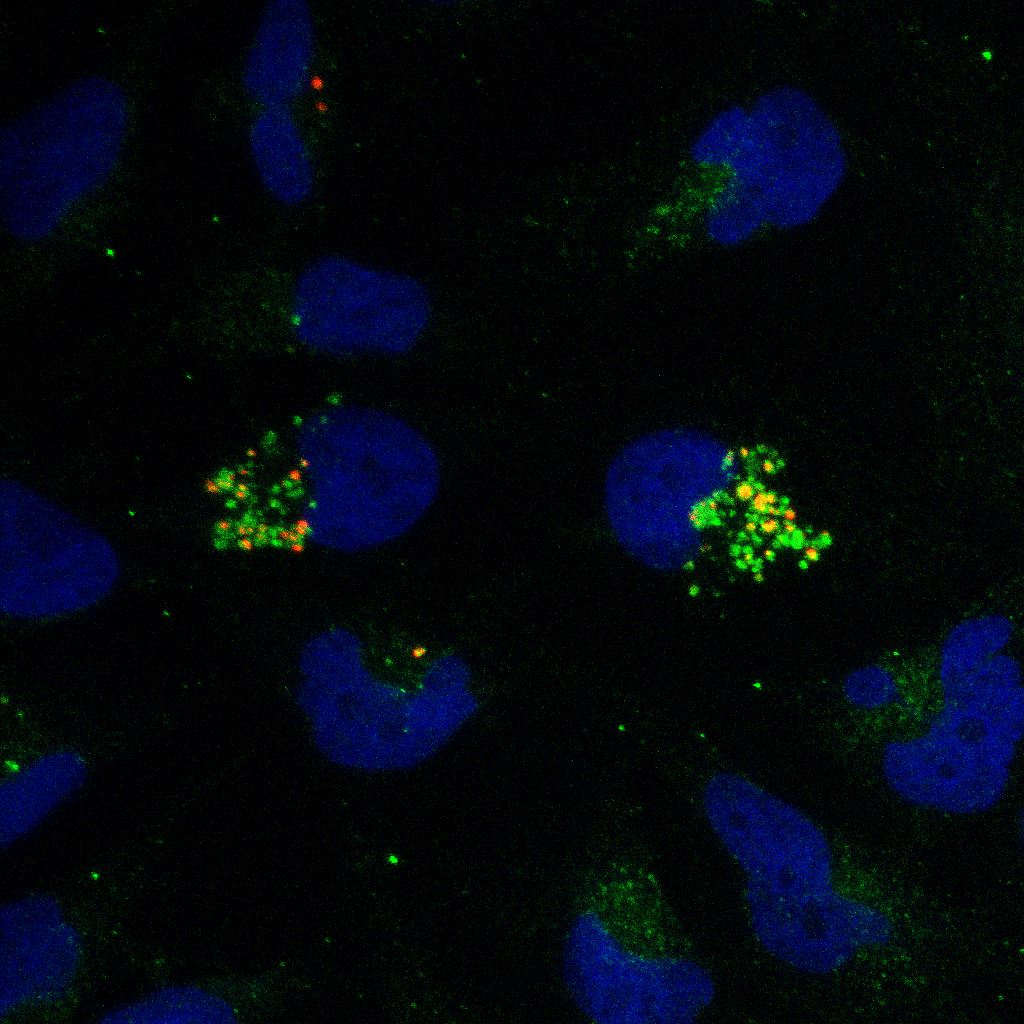

Supplement: Supplementary file 6 — Source data Fig. 5 [file 44318_2024_130_MOESM6_ESM.zip › Figure 5/5F/Merged.tif]

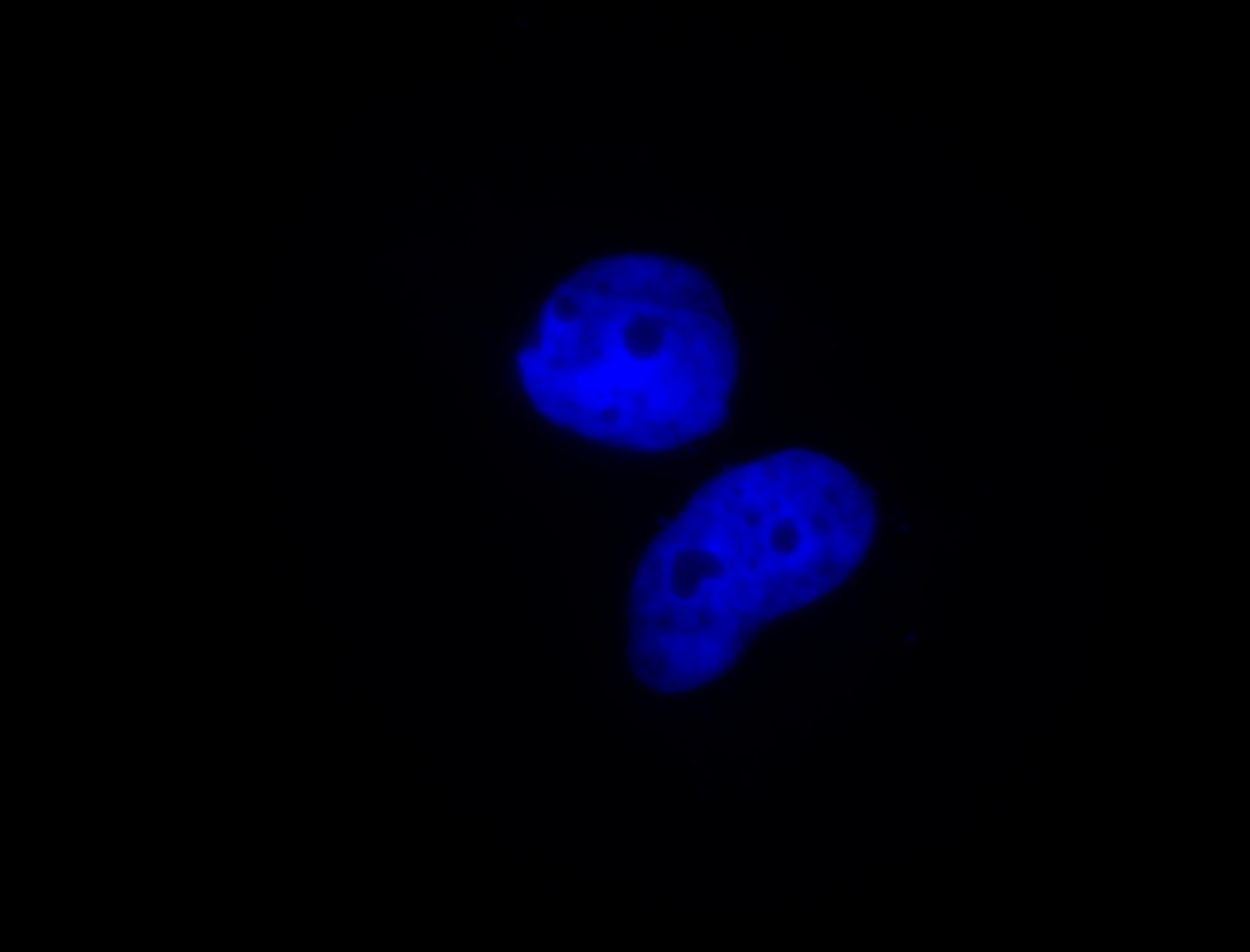

Supplement: Supplementary file 6 — Source data Fig. 5 [file 44318_2024_130_MOESM6_ESM.zip › Figure 5/5H/DAPI.tif]

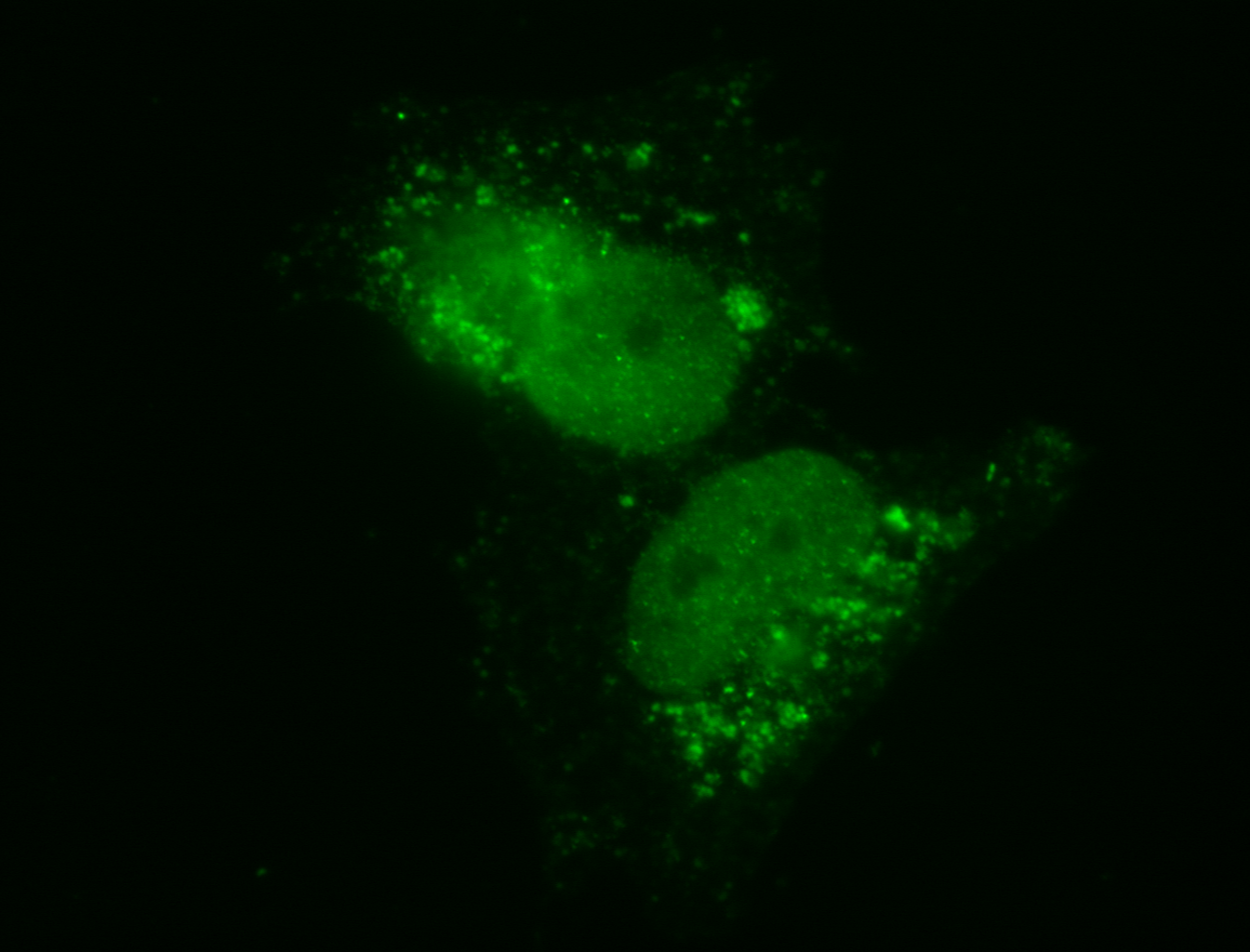

Supplement: Supplementary file 6 — Source data Fig. 5 [file 44318_2024_130_MOESM6_ESM.zip › Figure 5/5H/LC3B.tif]

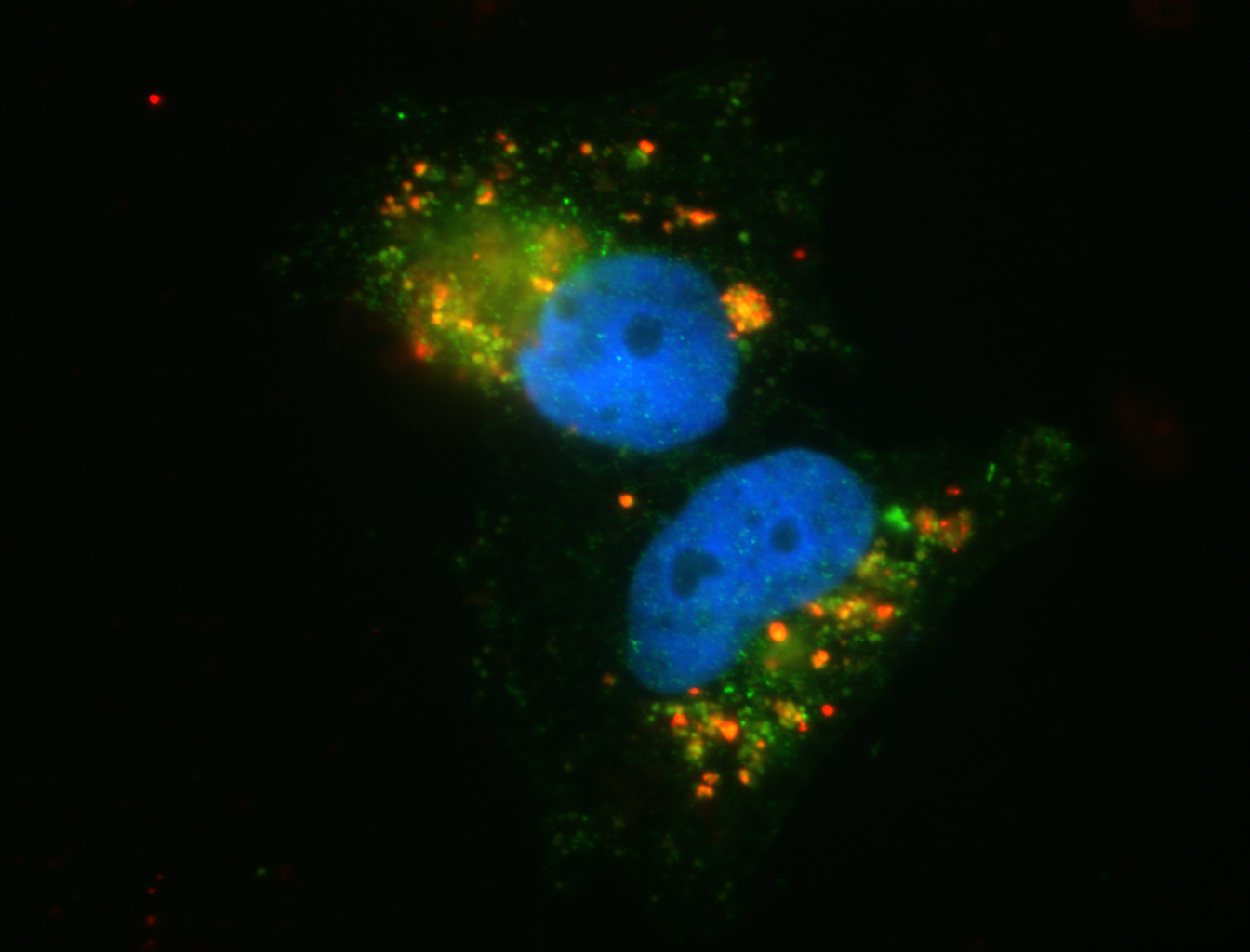

Supplement: Supplementary file 6 — Source data Fig. 5 [file 44318_2024_130_MOESM6_ESM.zip › Figure 5/5H/Merged.tif]

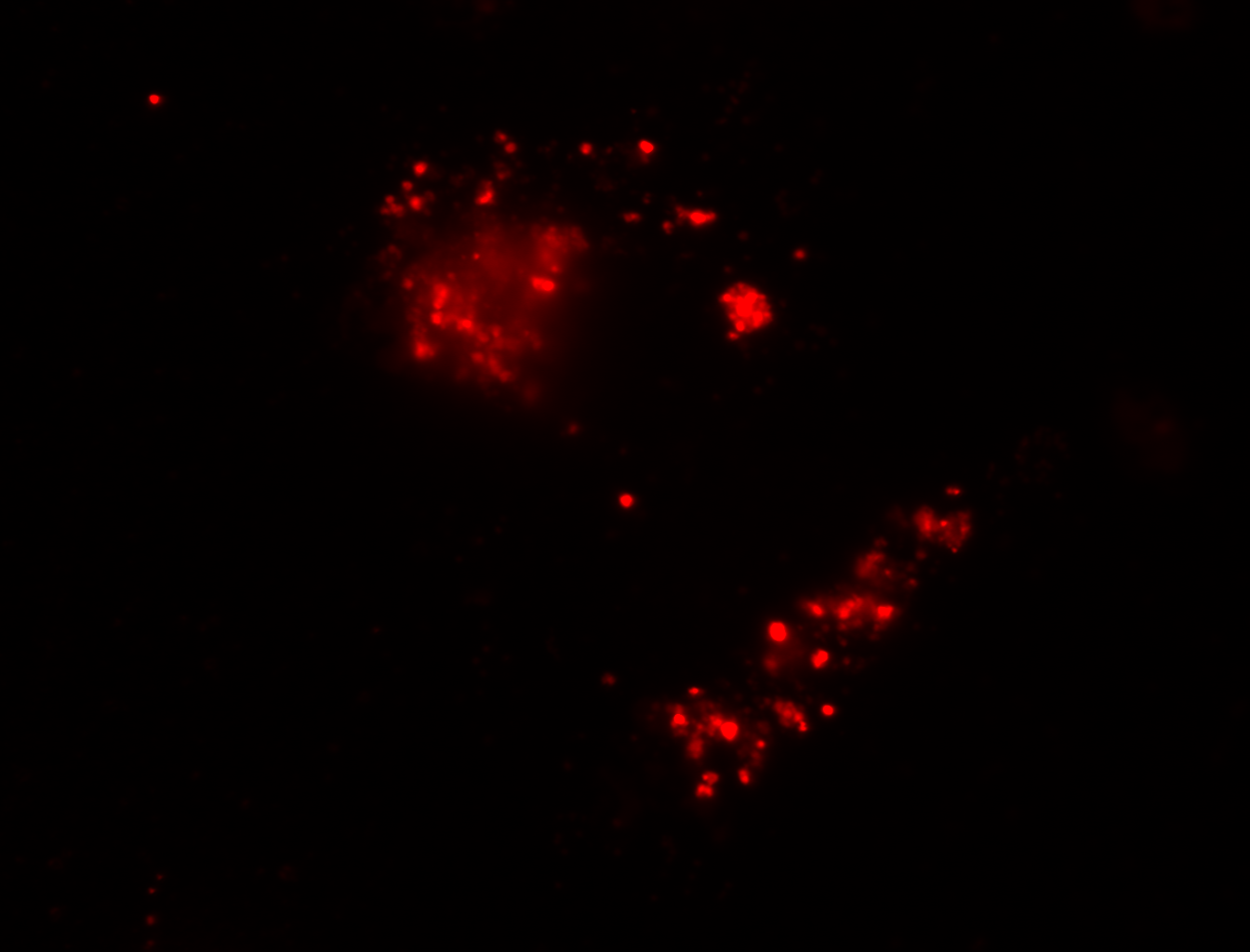

Supplement: Supplementary file 6 — Source data Fig. 5 [file 44318_2024_130_MOESM6_ESM.zip › Figure 5/5H/PKH26.tif]

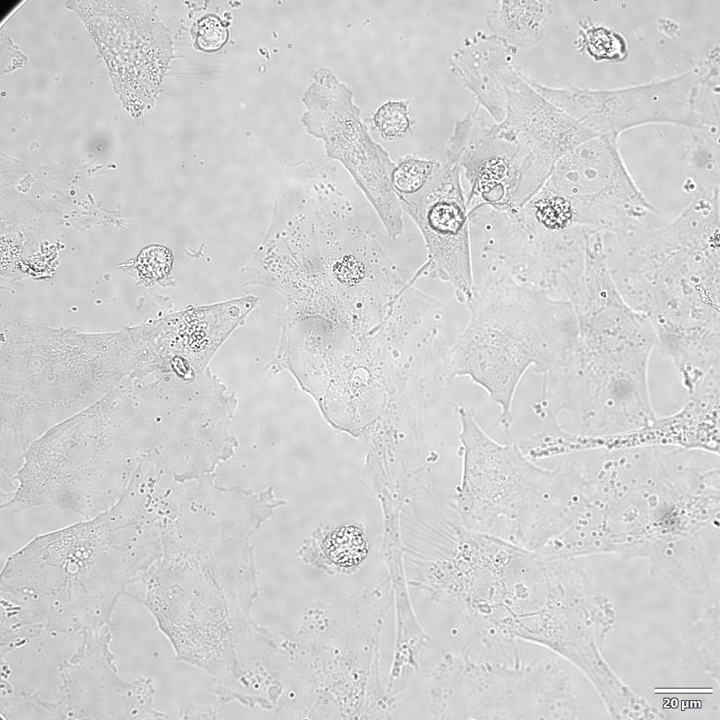

Supplement: Supplementary file 7 — Source data Fig. 6 [file 44318_2024_130_MOESM7_ESM.zip › Figure 6/6A/BF.tif]

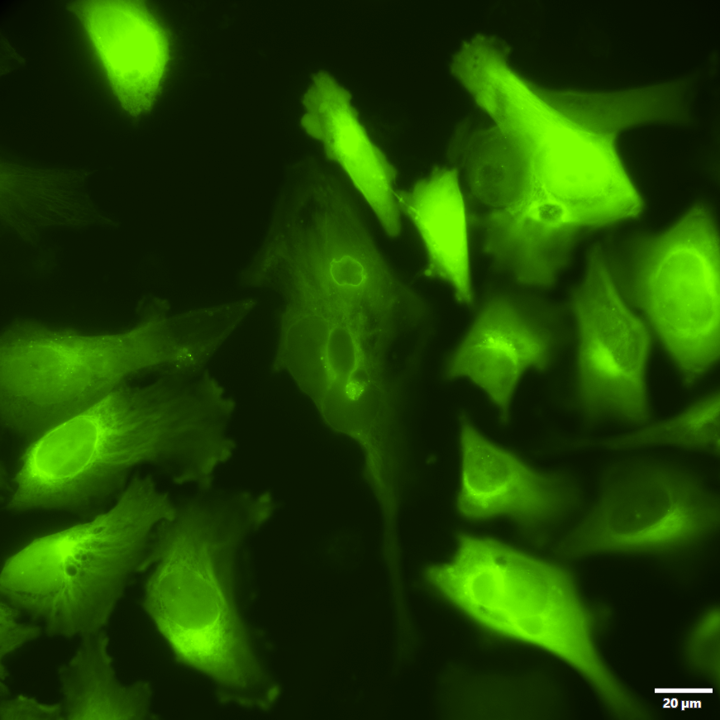

Supplement: Supplementary file 7 — Source data Fig. 6 [file 44318_2024_130_MOESM7_ESM.zip › Figure 6/6A/LC3B-GFP.tif]

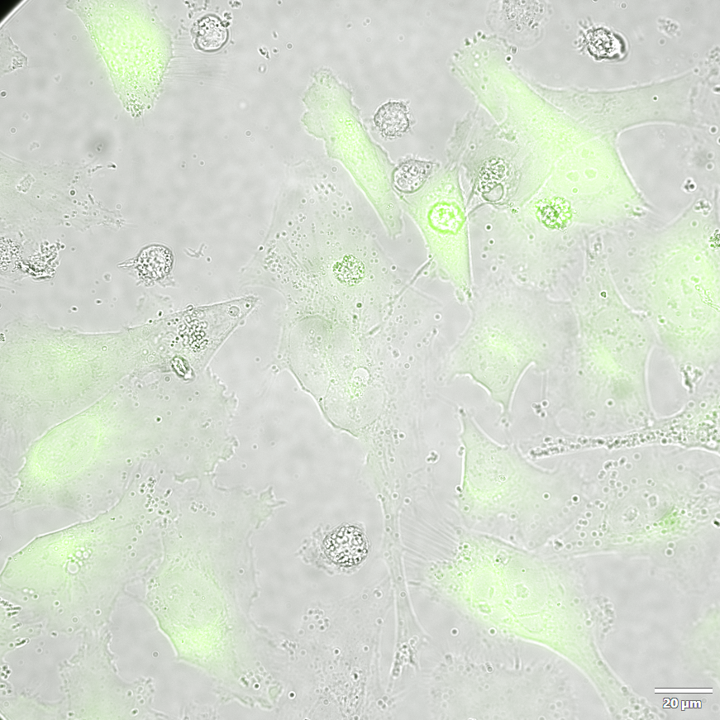

Supplement: Supplementary file 7 — Source data Fig. 6 [file 44318_2024_130_MOESM7_ESM.zip › Figure 6/6A/Merged.tif]

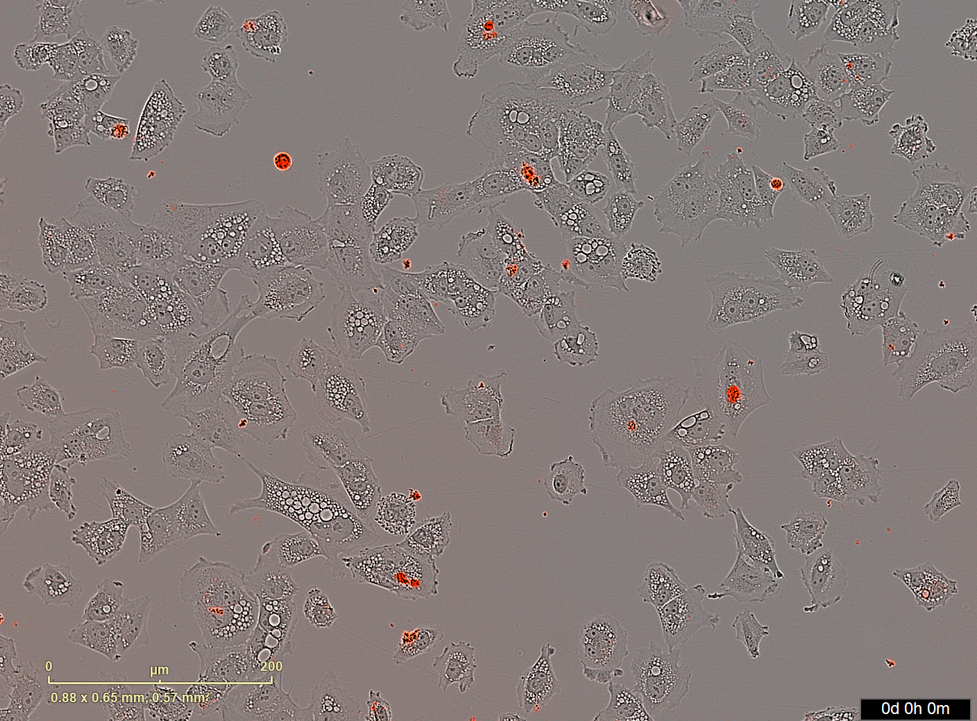

Supplement: Supplementary file 7 — Source data Fig. 6 [file 44318_2024_130_MOESM7_ESM.zip › Figure 6/6B/10 uM VPS34-IN1.tif]

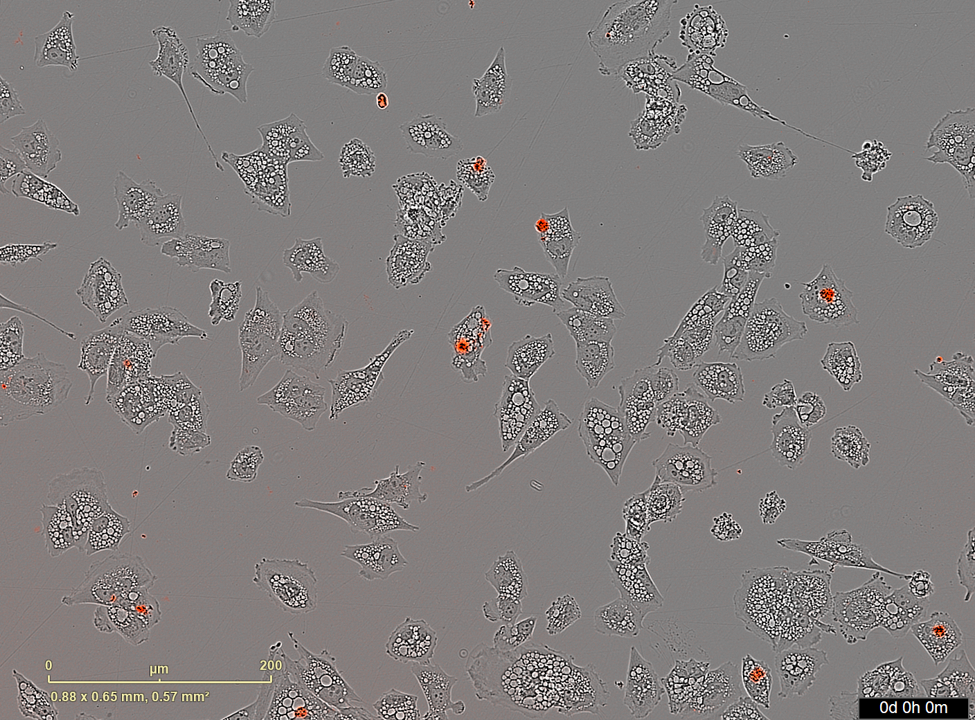

Supplement: Supplementary file 7 — Source data Fig. 6 [file 44318_2024_130_MOESM7_ESM.zip › Figure 6/6B/20 uM VPS34-IN1.tif]

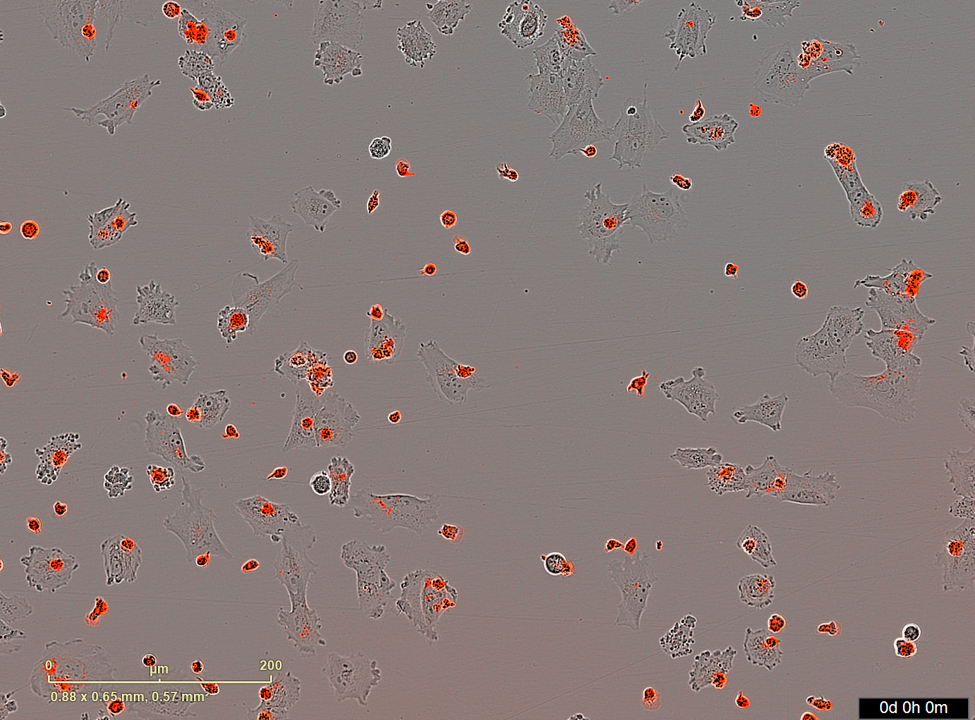

Supplement: Supplementary file 7 — Source data Fig. 6 [file 44318_2024_130_MOESM7_ESM.zip › Figure 6/6B/DMSO.tif]

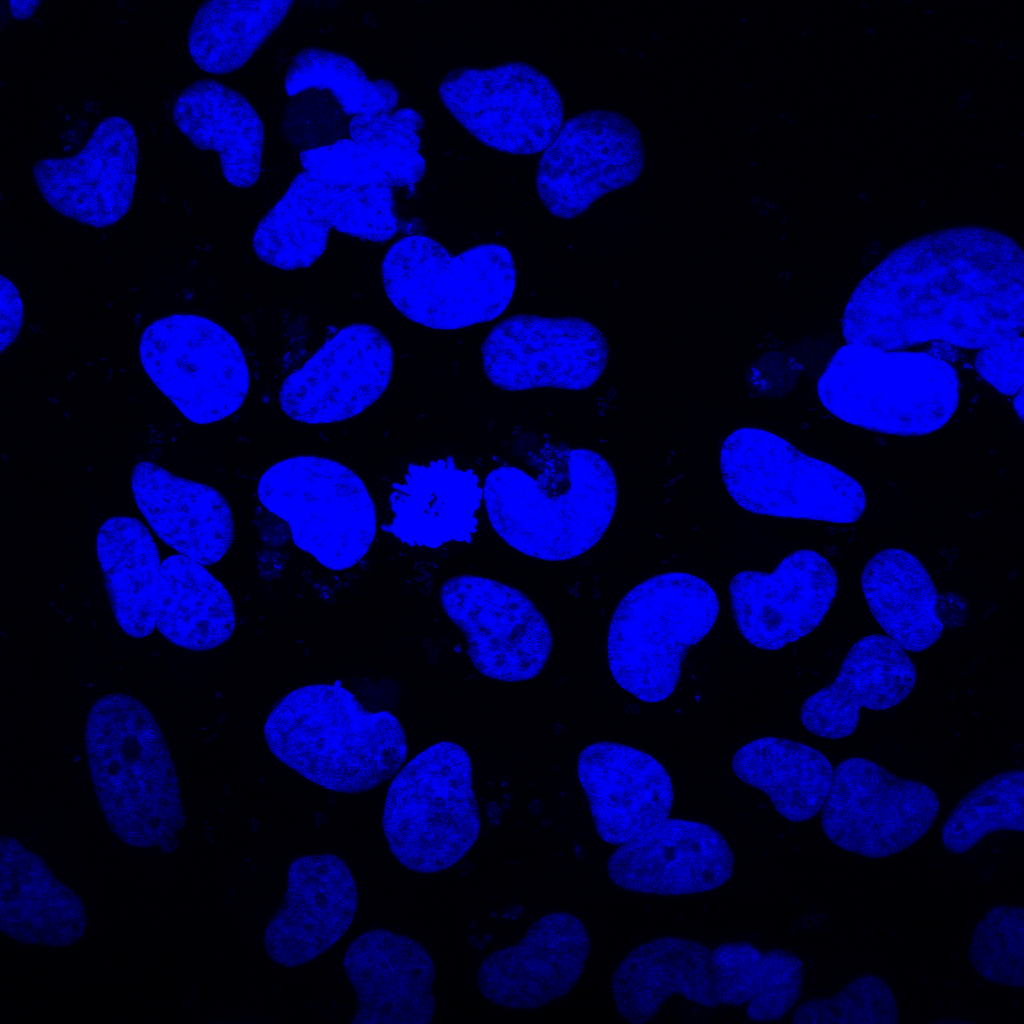

Supplement: Supplementary file 7 — Source data Fig. 6 [file 44318_2024_130_MOESM7_ESM.zip › Figure 6/6H/SC (DMSO) control/MAX_LN229-4SA TAN coculture shVPS34 VPS34in1 MPO PKH.lif - sc dmso 5 - C=0.tif]

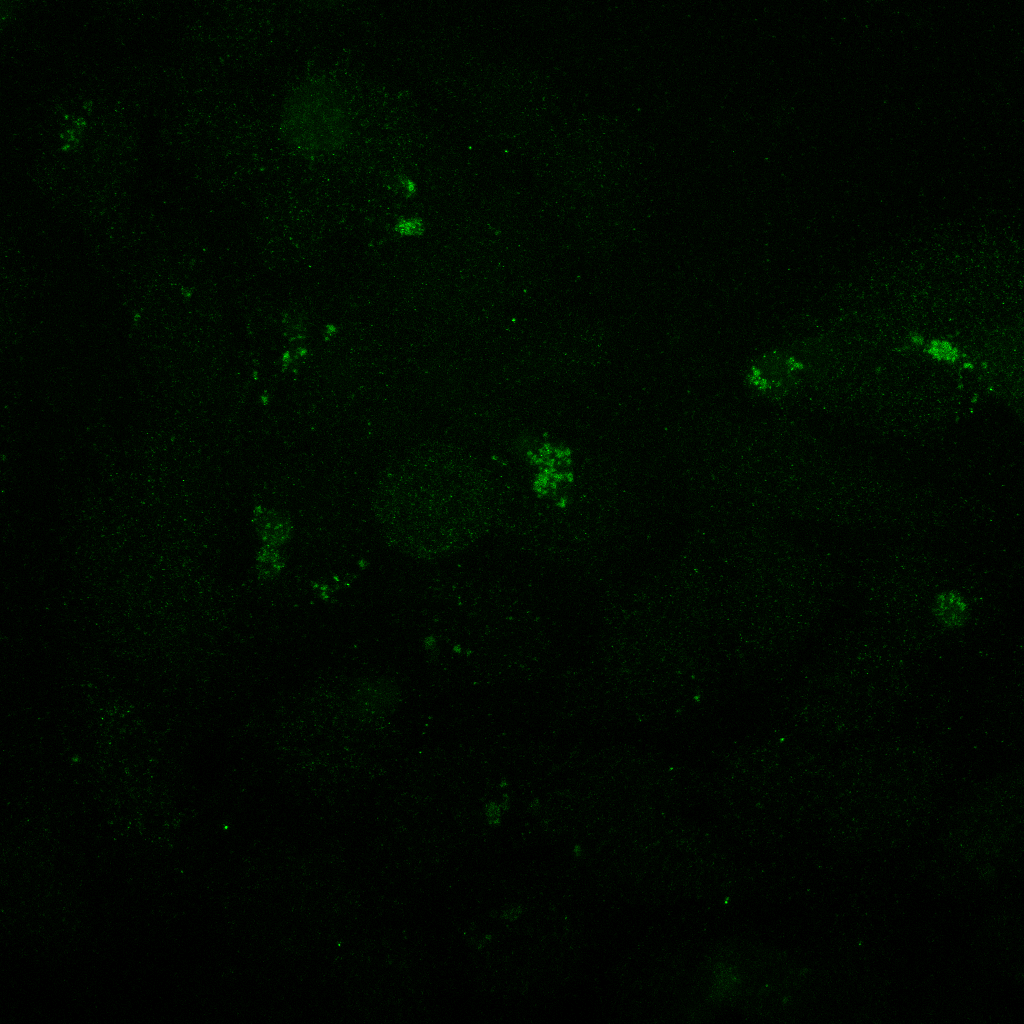

Supplement: Supplementary file 7 — Source data Fig. 6 [file 44318_2024_130_MOESM7_ESM.zip › Figure 6/6H/SC (DMSO) control/MAX_LN229-4SA TAN coculture shVPS34 VPS34in1 MPO PKH.lif - sc dmso 5 - C=1.tif]

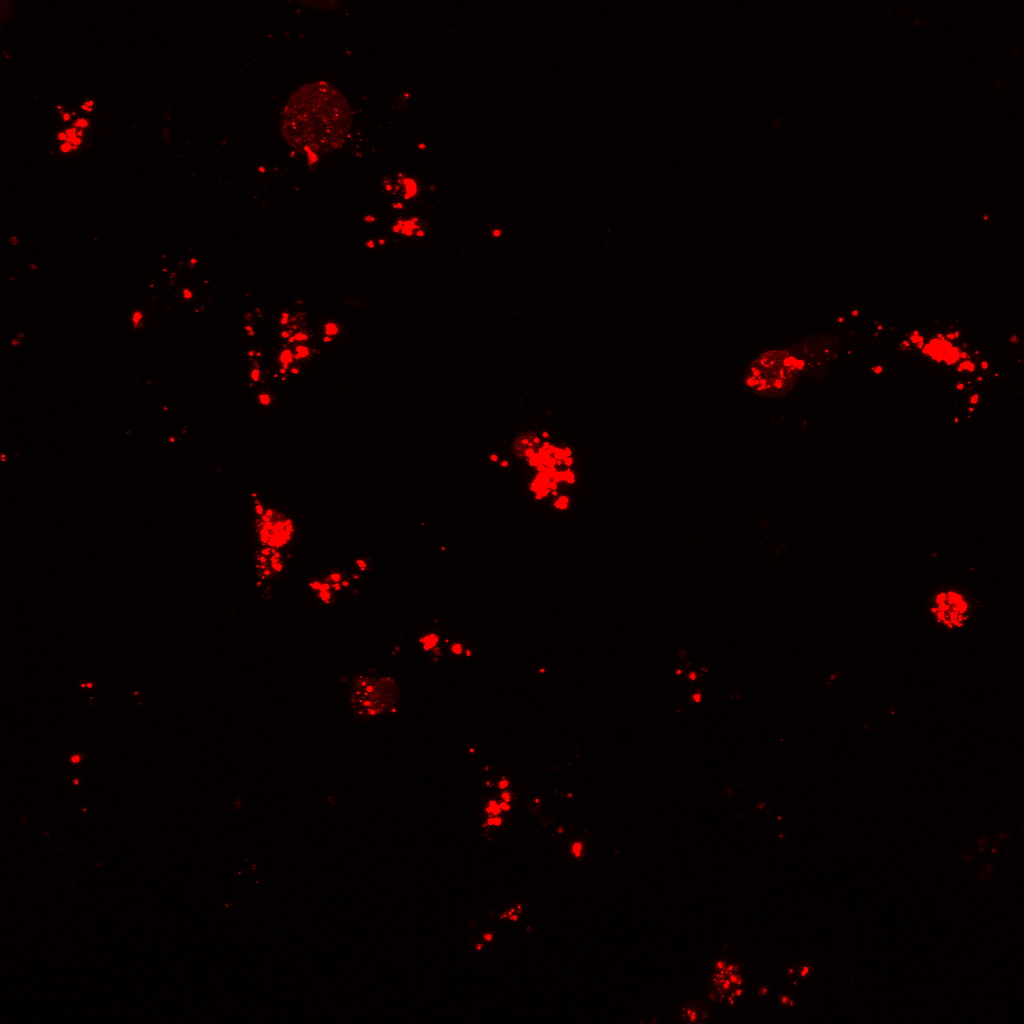

Supplement: Supplementary file 7 — Source data Fig. 6 [file 44318_2024_130_MOESM7_ESM.zip › Figure 6/6H/SC (DMSO) control/MAX_LN229-4SA TAN coculture shVPS34 VPS34in1 MPO PKH.lif - sc dmso 5 - C=2.tif]

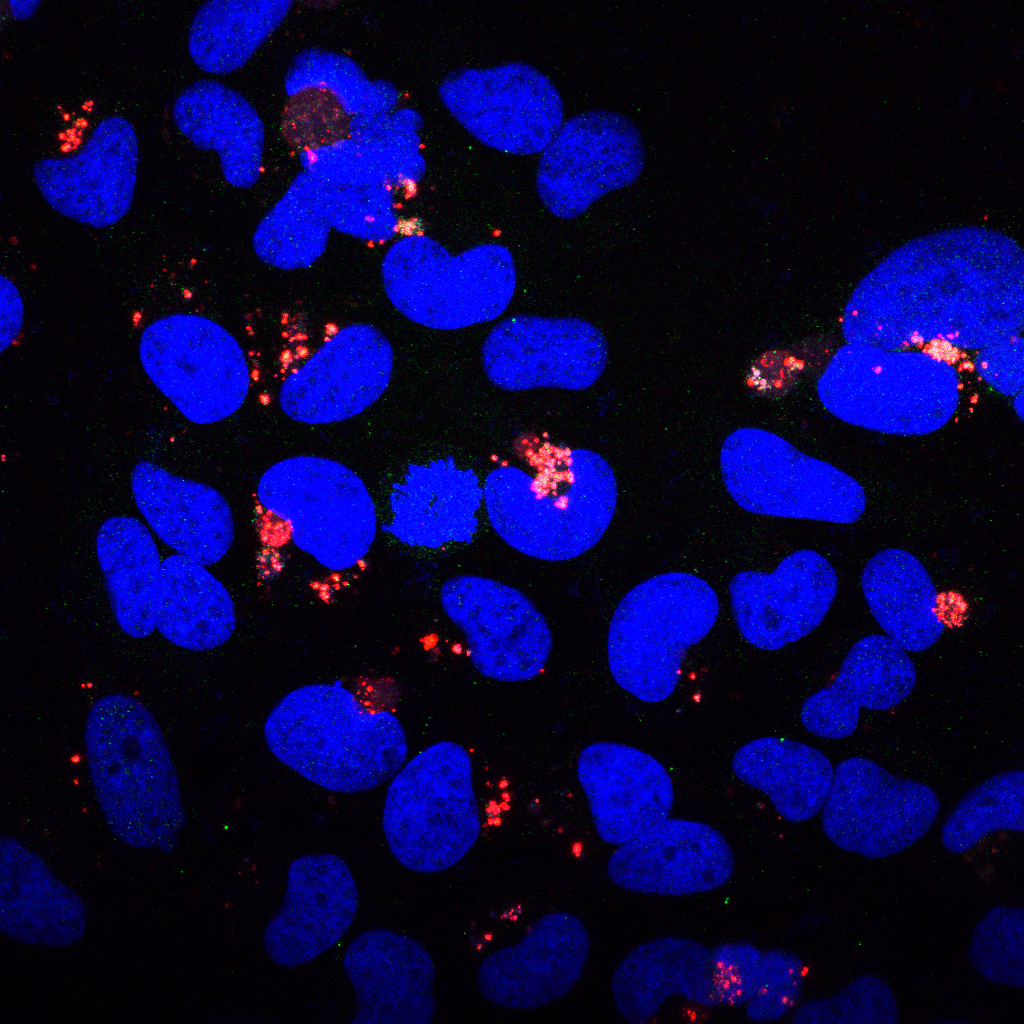

Supplement: Supplementary file 7 — Source data Fig. 6 [file 44318_2024_130_MOESM7_ESM.zip › Figure 6/6H/SC (DMSO) control/merged.tif]

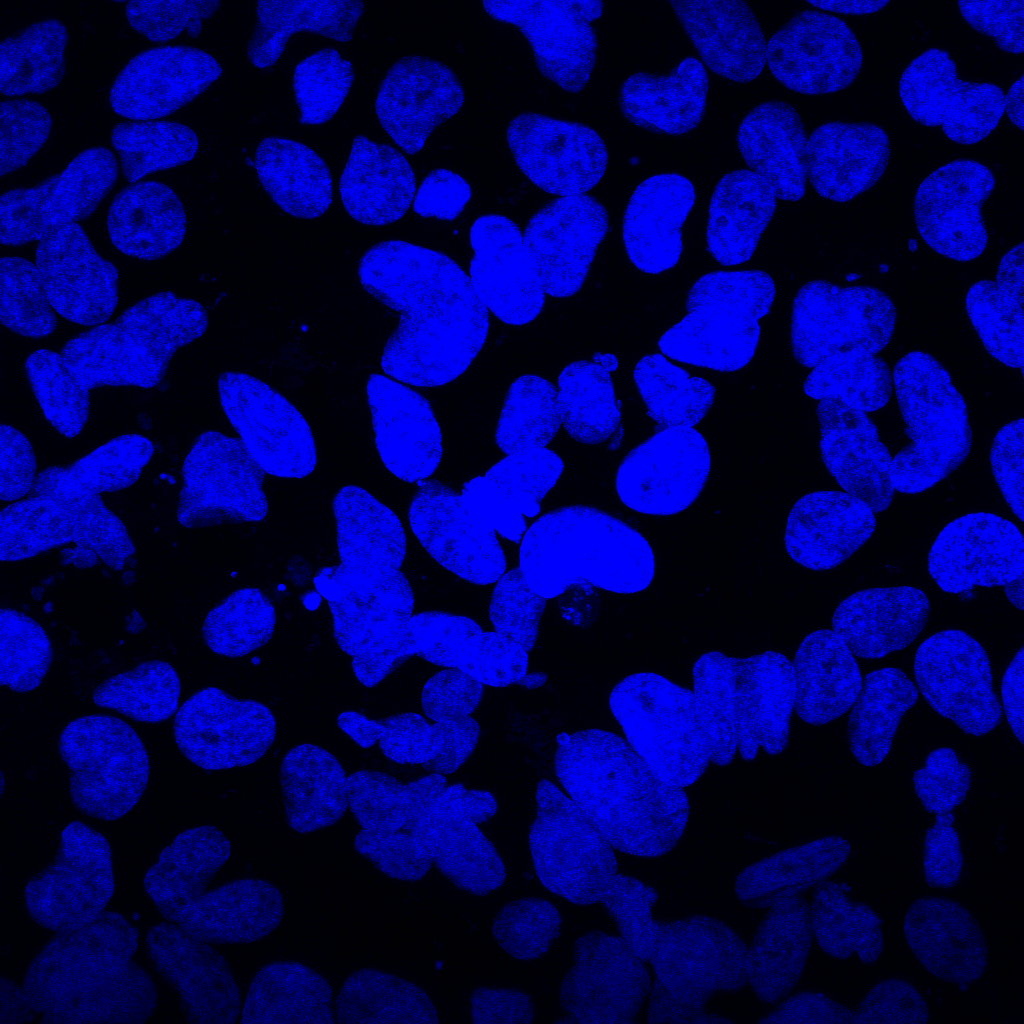

Supplement: Supplementary file 7 — Source data Fig. 6 [file 44318_2024_130_MOESM7_ESM.zip › Figure 6/6H/shVPS34 94/MAX_LN229-4SA TAN coculture shVPS34 VPS34in1 MPO PKH.lif - shvps34 94 3 - C=0.tif]

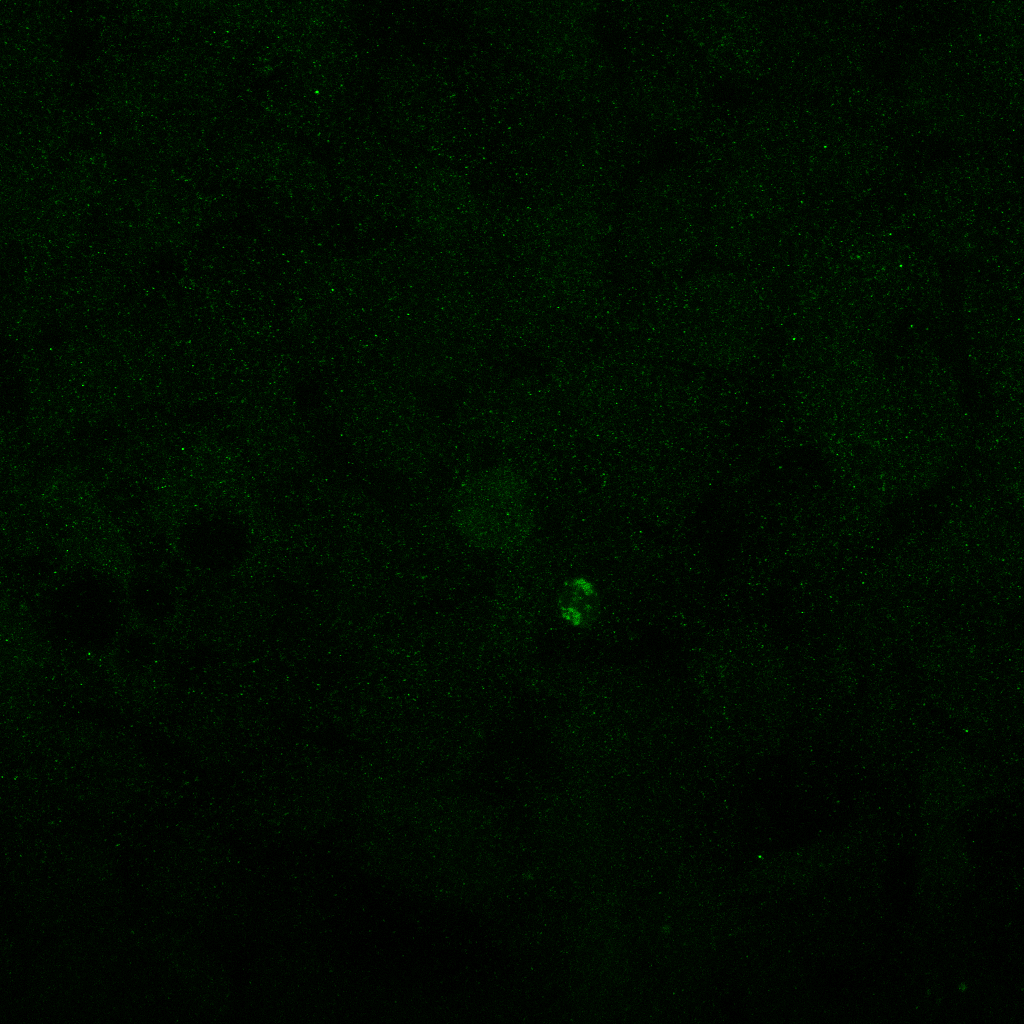

Supplement: Supplementary file 7 — Source data Fig. 6 [file 44318_2024_130_MOESM7_ESM.zip › Figure 6/6H/shVPS34 94/MAX_LN229-4SA TAN coculture shVPS34 VPS34in1 MPO PKH.lif - shvps34 94 3 - C=1.tif]

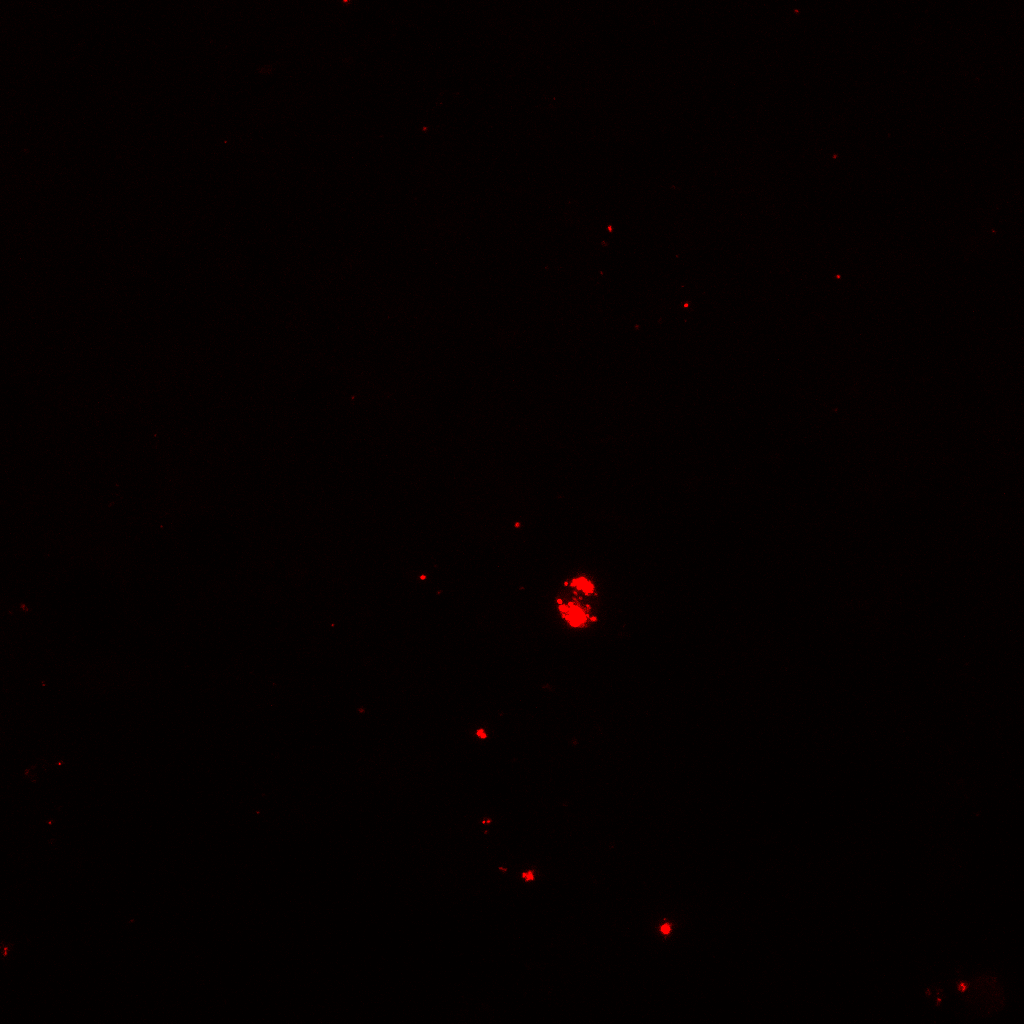

Supplement: Supplementary file 7 — Source data Fig. 6 [file 44318_2024_130_MOESM7_ESM.zip › Figure 6/6H/shVPS34 94/MAX_LN229-4SA TAN coculture shVPS34 VPS34in1 MPO PKH.lif - shvps34 94 3 - C=2.tif]

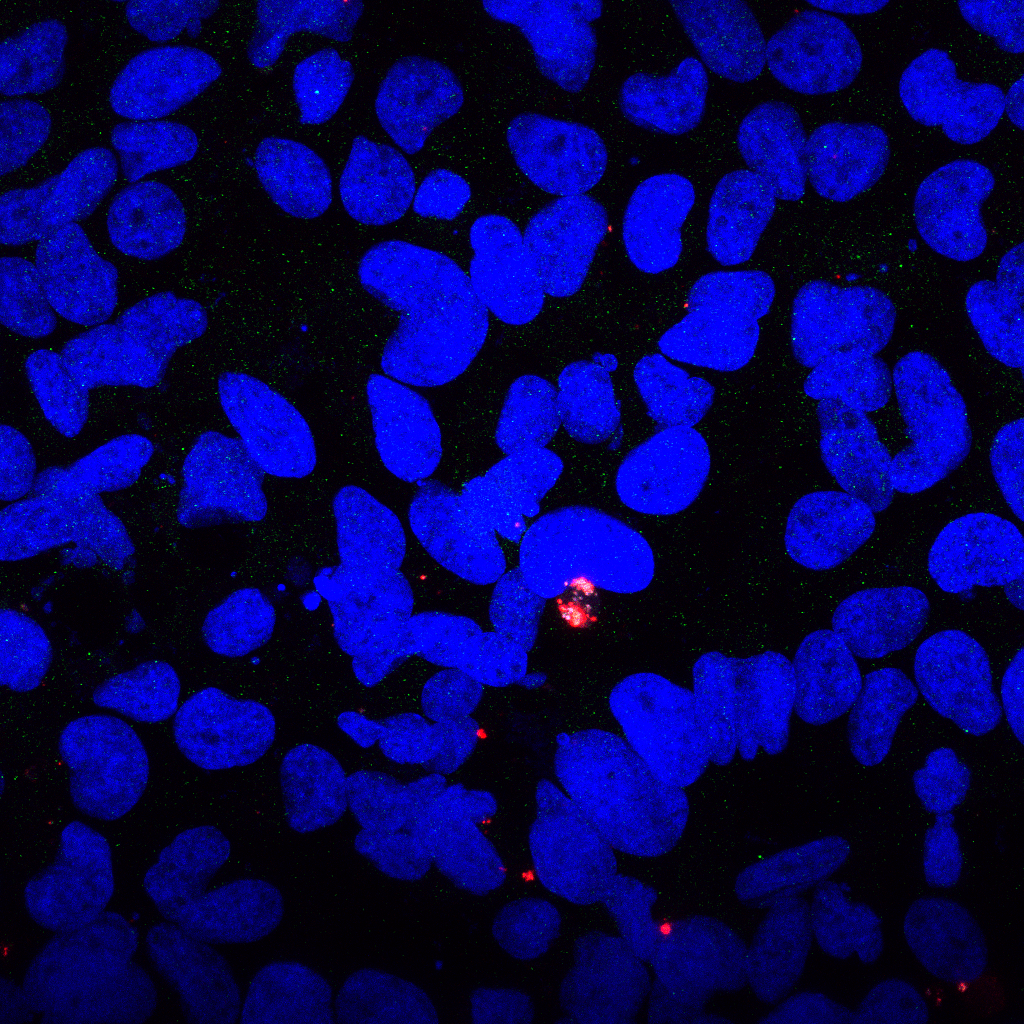

Supplement: Supplementary file 7 — Source data Fig. 6 [file 44318_2024_130_MOESM7_ESM.zip › Figure 6/6H/shVPS34 94/merged.tif]

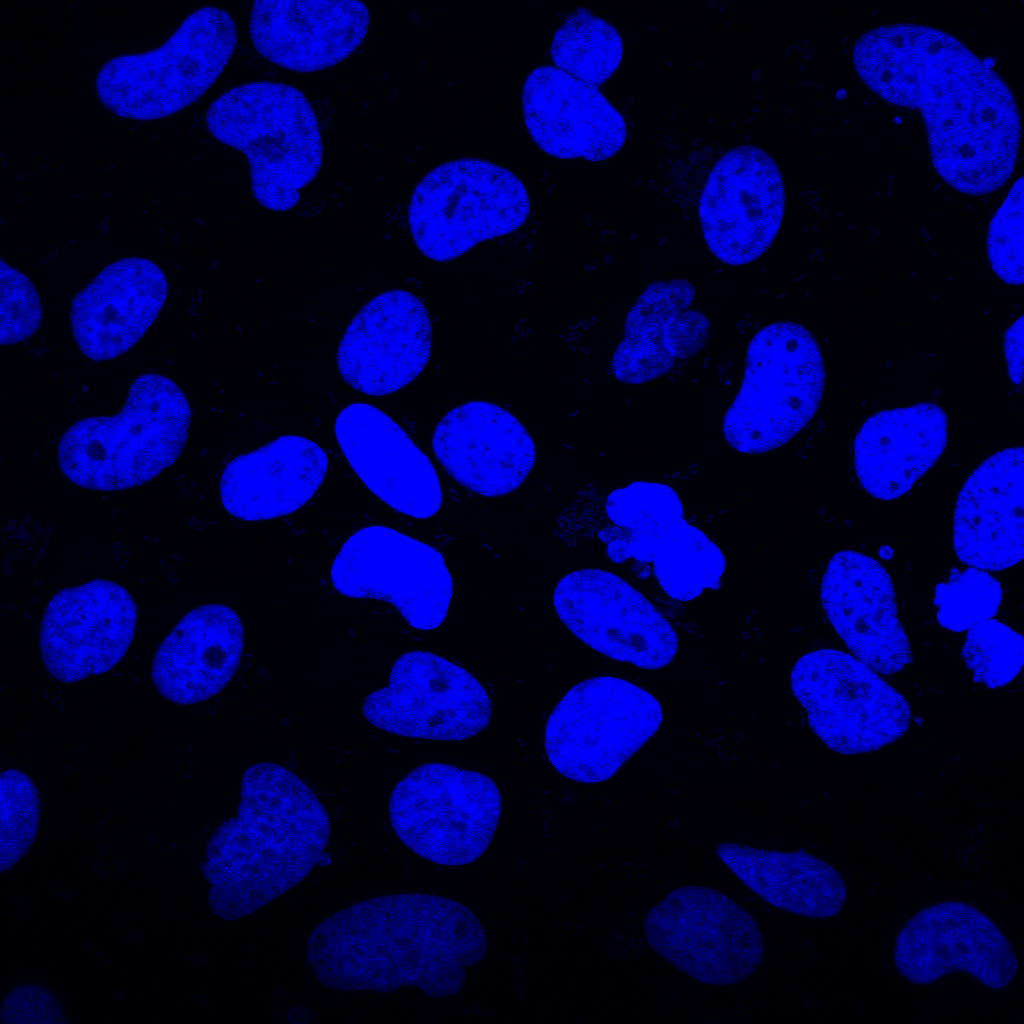

Supplement: Supplementary file 7 — Source data Fig. 6 [file 44318_2024_130_MOESM7_ESM.zip › Figure 6/6H/shVPS34 96/MAX_LN229-4SA TAN coculture shVPS34 VPS34in1 MPO PKH.lif - shvps34 96 3 - C=0.tif]

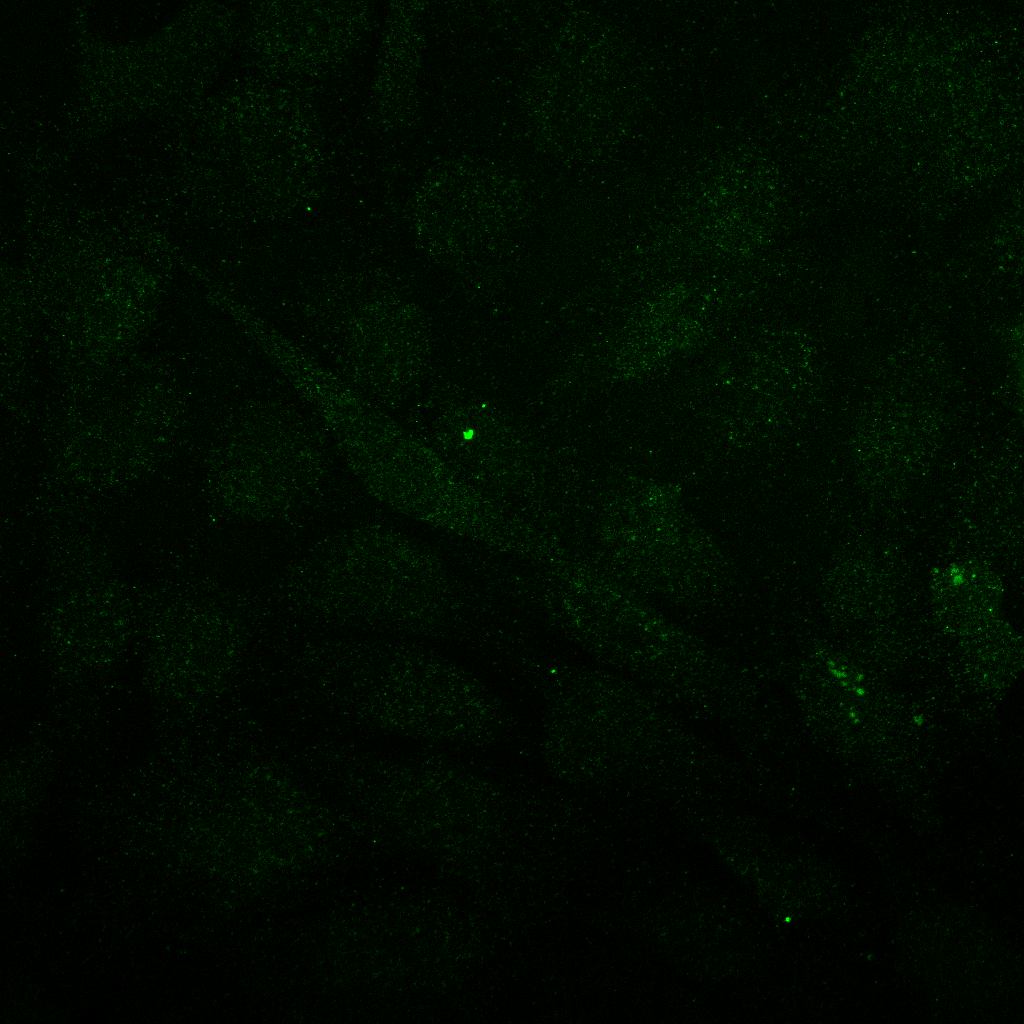

Supplement: Supplementary file 7 — Source data Fig. 6 [file 44318_2024_130_MOESM7_ESM.zip › Figure 6/6H/shVPS34 96/MAX_LN229-4SA TAN coculture shVPS34 VPS34in1 MPO PKH.lif - shvps34 96 3 - C=1.tif]

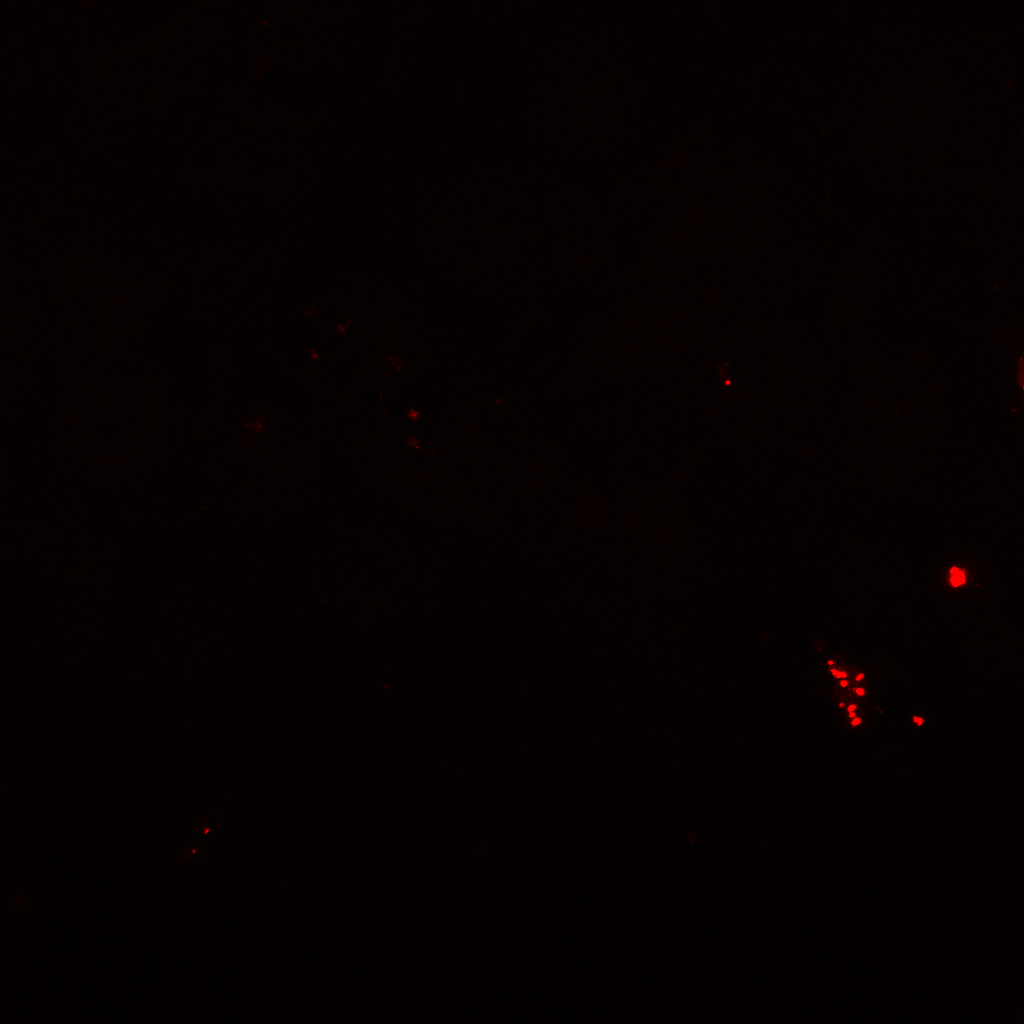

Supplement: Supplementary file 7 — Source data Fig. 6 [file 44318_2024_130_MOESM7_ESM.zip › Figure 6/6H/shVPS34 96/MAX_LN229-4SA TAN coculture shVPS34 VPS34in1 MPO PKH.lif - shvps34 96 3 - C=2.tif]

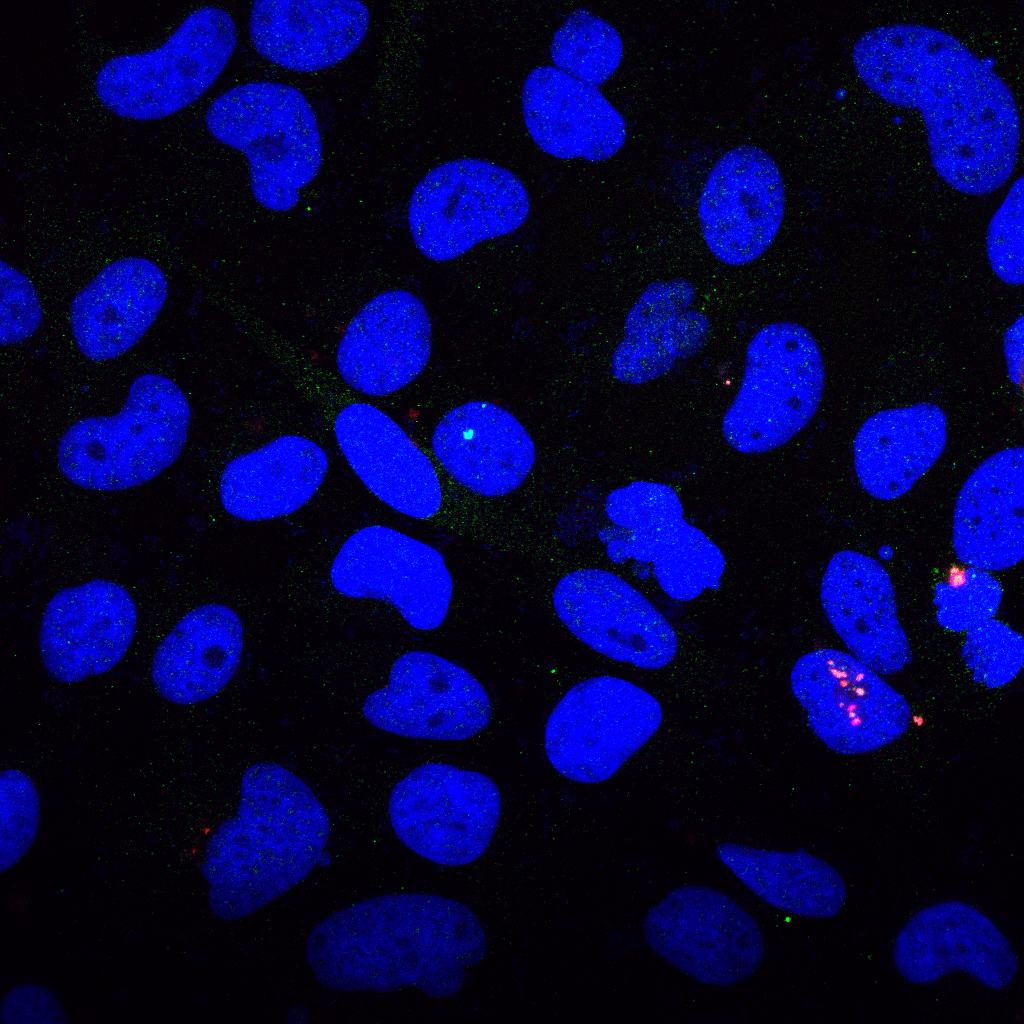

Supplement: Supplementary file 7 — Source data Fig. 6 [file 44318_2024_130_MOESM7_ESM.zip › Figure 6/6H/shVPS34 96/merged.tif]

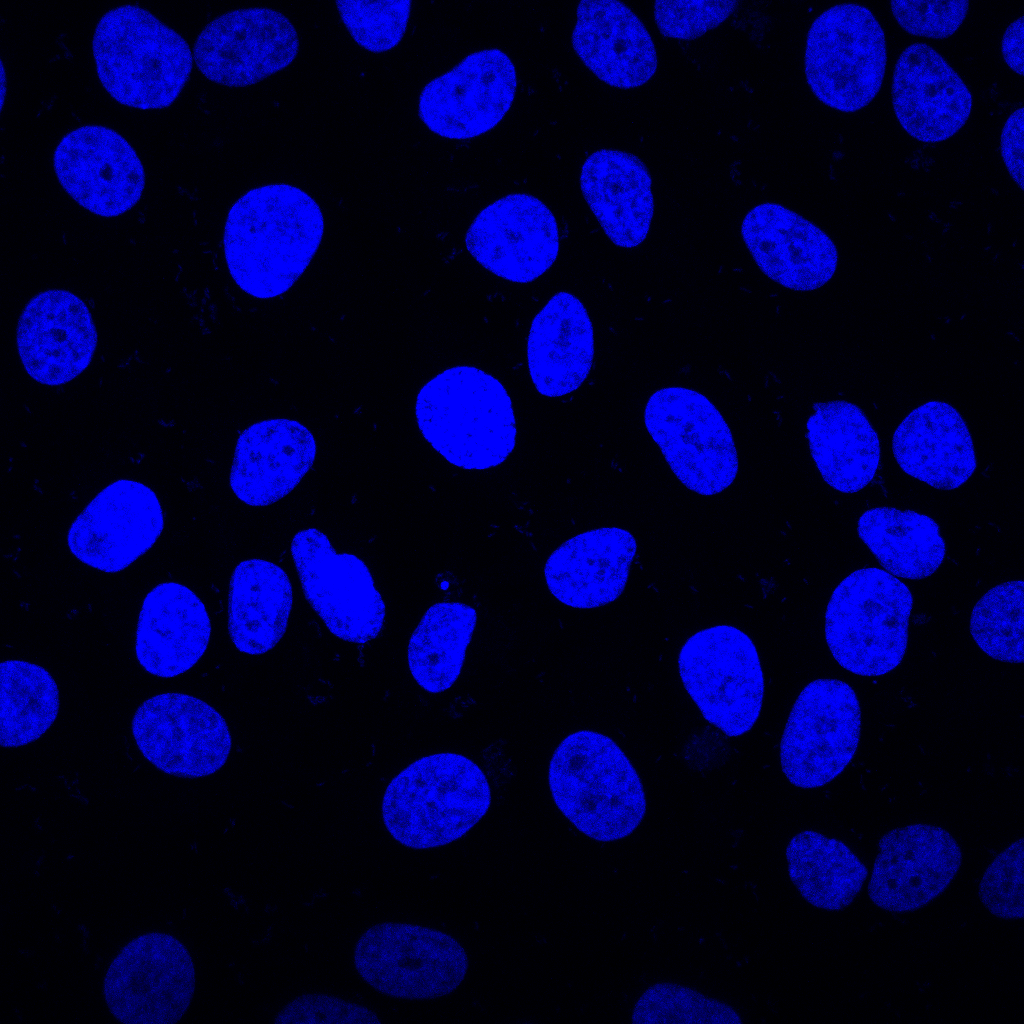

Supplement: Supplementary file 7 — Source data Fig. 6 [file 44318_2024_130_MOESM7_ESM.zip › Figure 6/6H/VPS34IN1 10 uM/MAX_LN229-4SA TAN coculture shVPS34 VPS34in1 MPO PKH.lif - vps34 in1 2 - C=0.tif]

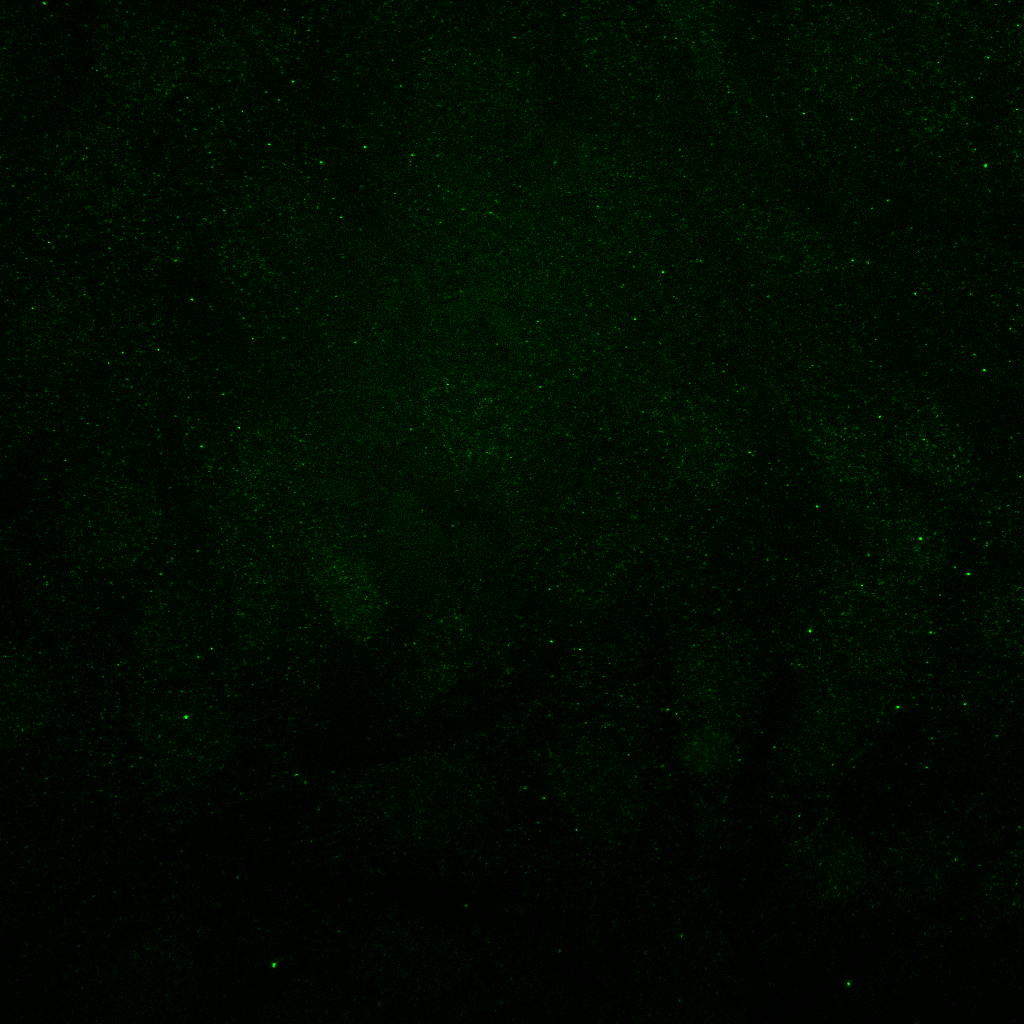

Supplement: Supplementary file 7 — Source data Fig. 6 [file 44318_2024_130_MOESM7_ESM.zip › Figure 6/6H/VPS34IN1 10 uM/MAX_LN229-4SA TAN coculture shVPS34 VPS34in1 MPO PKH.lif - vps34 in1 2 - C=1.tif]

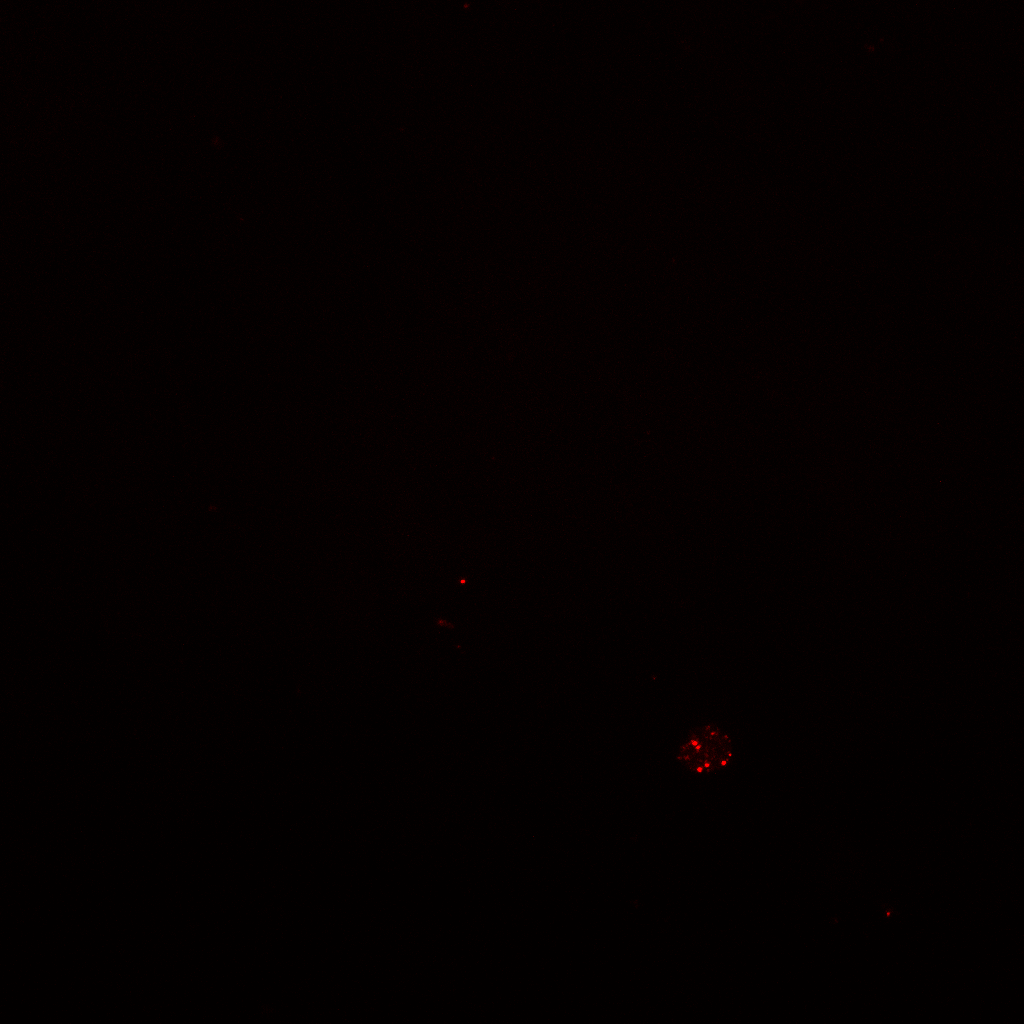

Supplement: Supplementary file 7 — Source data Fig. 6 [file 44318_2024_130_MOESM7_ESM.zip › Figure 6/6H/VPS34IN1 10 uM/MAX_LN229-4SA TAN coculture shVPS34 VPS34in1 MPO PKH.lif - vps34 in1 2 - C=2.tif]

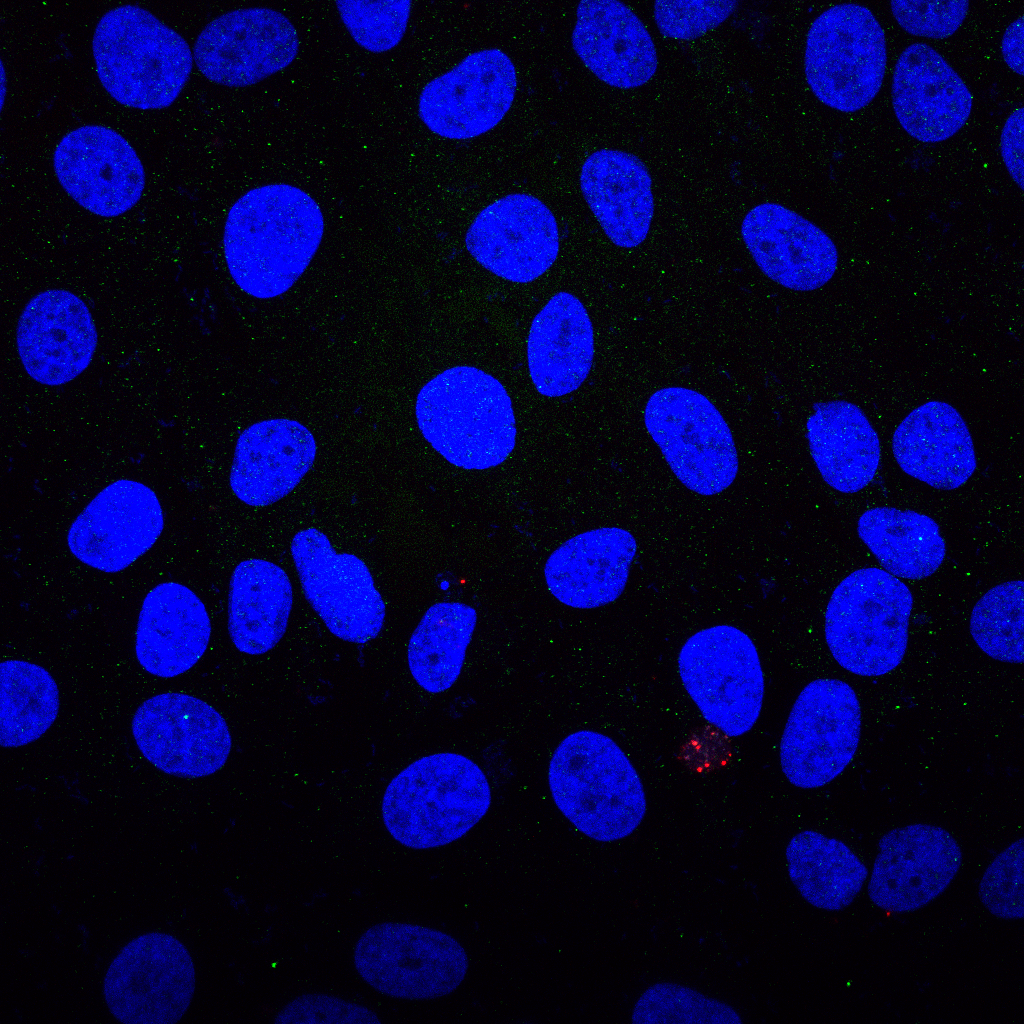

Supplement: Supplementary file 7 — Source data Fig. 6 [file 44318_2024_130_MOESM7_ESM.zip › Figure 6/6H/VPS34IN1 10 uM/merged.tif]

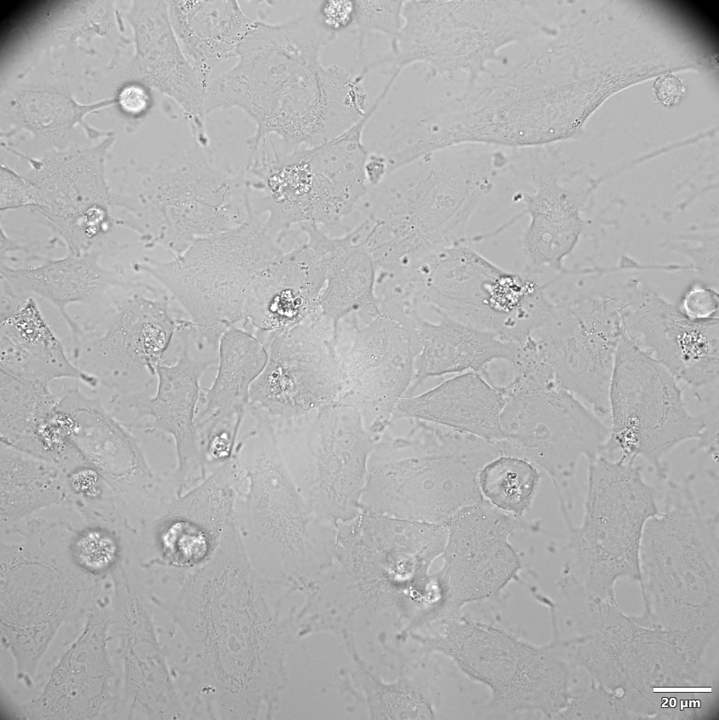

Supplement: Supplementary file 7 — Source data Fig. 6 [file 44318_2024_130_MOESM7_ESM.zip › Figure 6/6M/BF.tif]

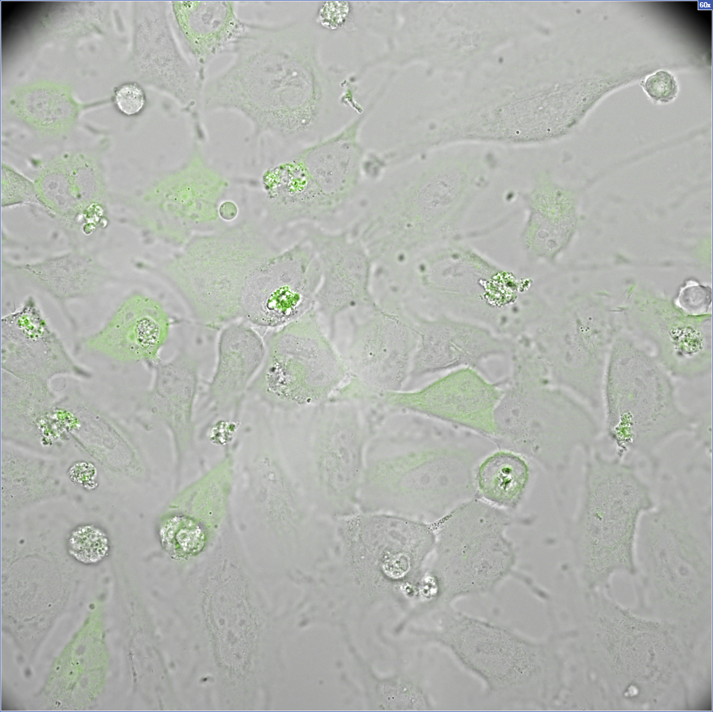

Supplement: Supplementary file 7 — Source data Fig. 6 [file 44318_2024_130_MOESM7_ESM.zip › Figure 6/6M/Merged.tif]

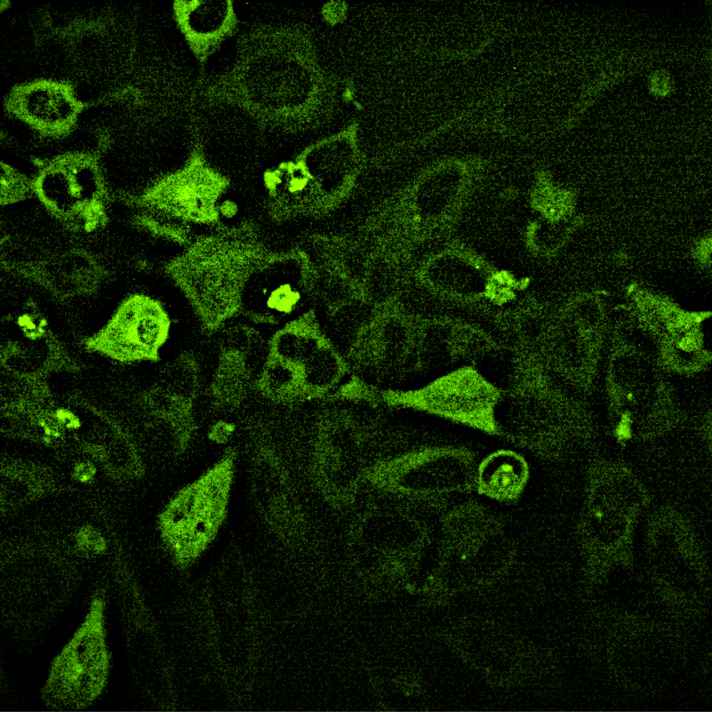

Supplement: Supplementary file 7 — Source data Fig. 6 [file 44318_2024_130_MOESM7_ESM.zip › Figure 6/6M/UVRAG-GFP.tif]

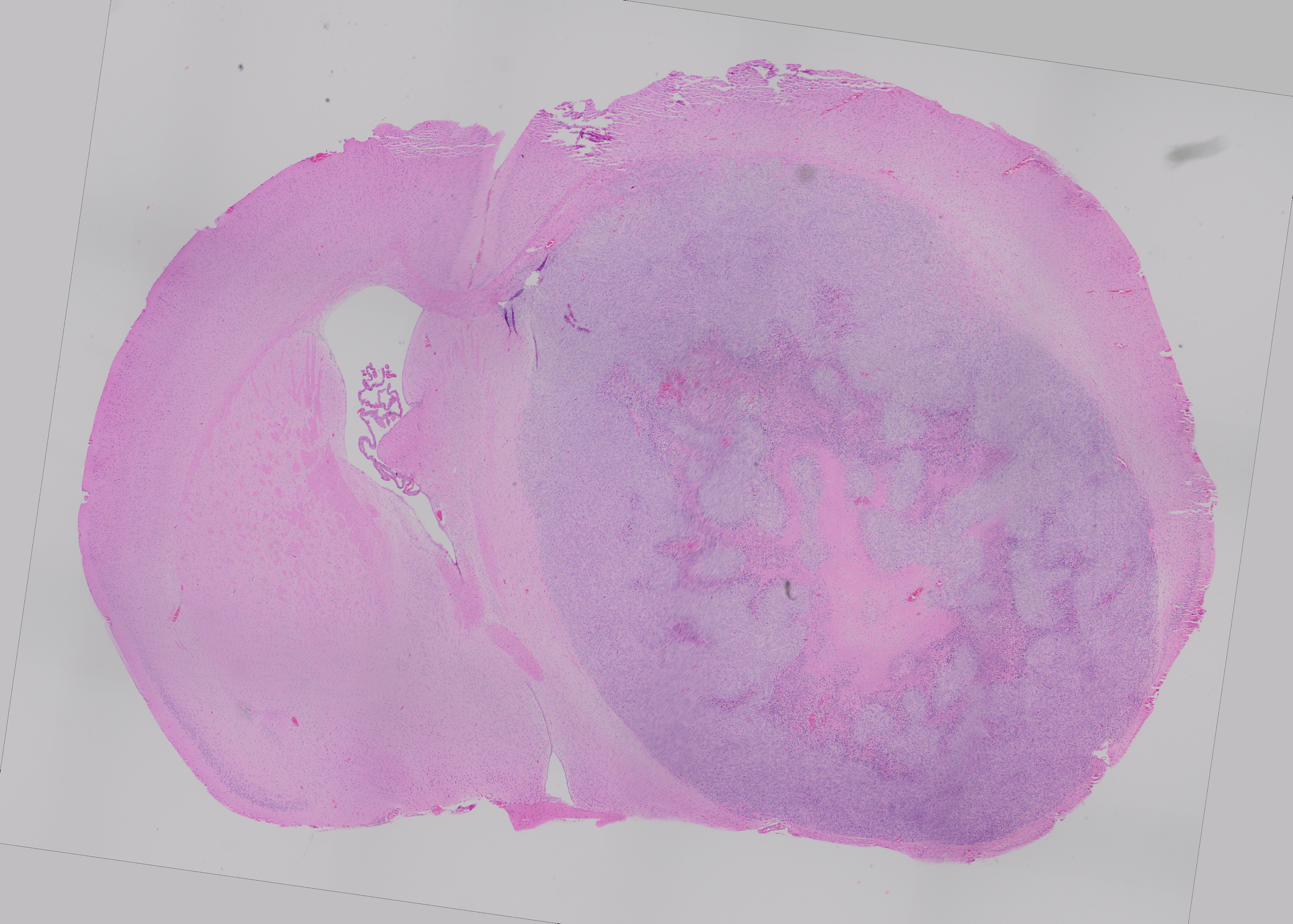

Supplement: Supplementary file 8 — Source data Fig. 7 [file 44318_2024_130_MOESM8_ESM.zip › Figure 7/7C/7C 4-ABAH.tif]
