# Supplementary figures and images for: LC3-associated phagocytosis of neutrophils triggers tumor ferroptotic cell death in glioblastoma (part 2 of 2)
Source: EMBO J. 2024 May 28;43(13):4. doi: 10.1038/s44318-024-00130-4 (PMC11217441; doi:10.1038/s44318-024-00130-4)

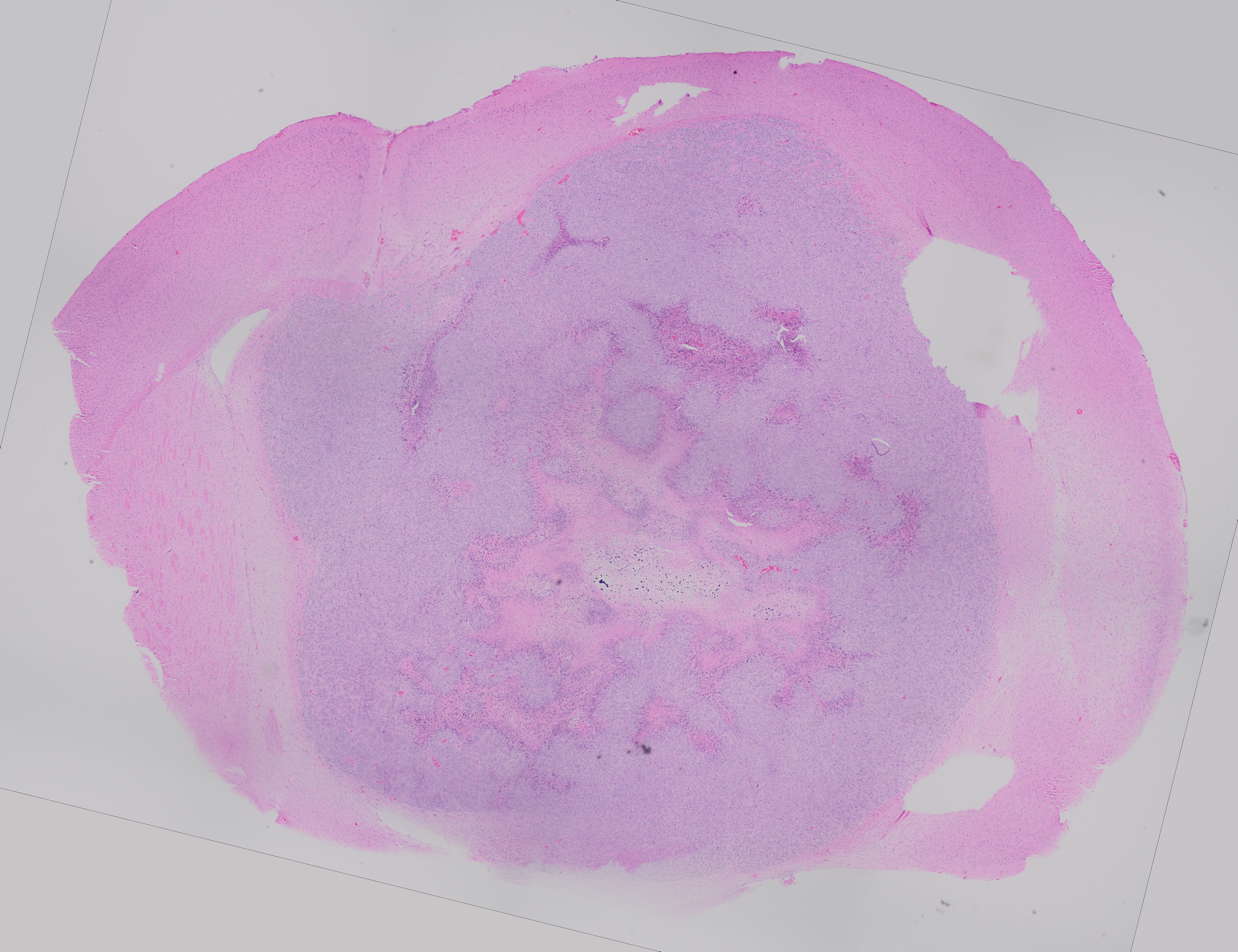

Supplement: Supplementary file 8 — Source data Fig. 7 [file 44318_2024_130_MOESM8_ESM.zip › Figure 7/7C/7C Lipr-1.tif]

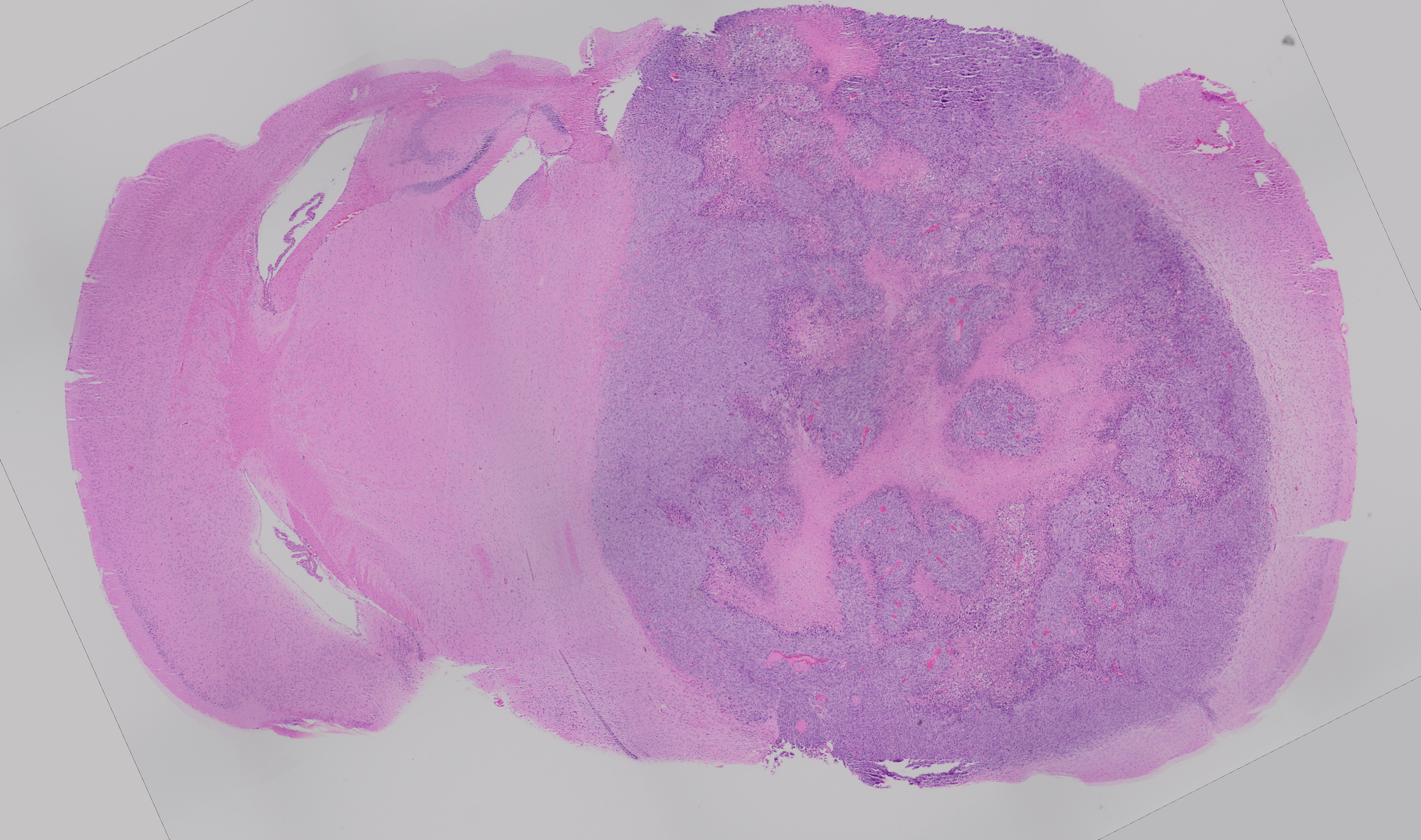

Supplement: Supplementary file 8 — Source data Fig. 7 [file 44318_2024_130_MOESM8_ESM.zip › Figure 7/7C/7C Vehicle.tif]

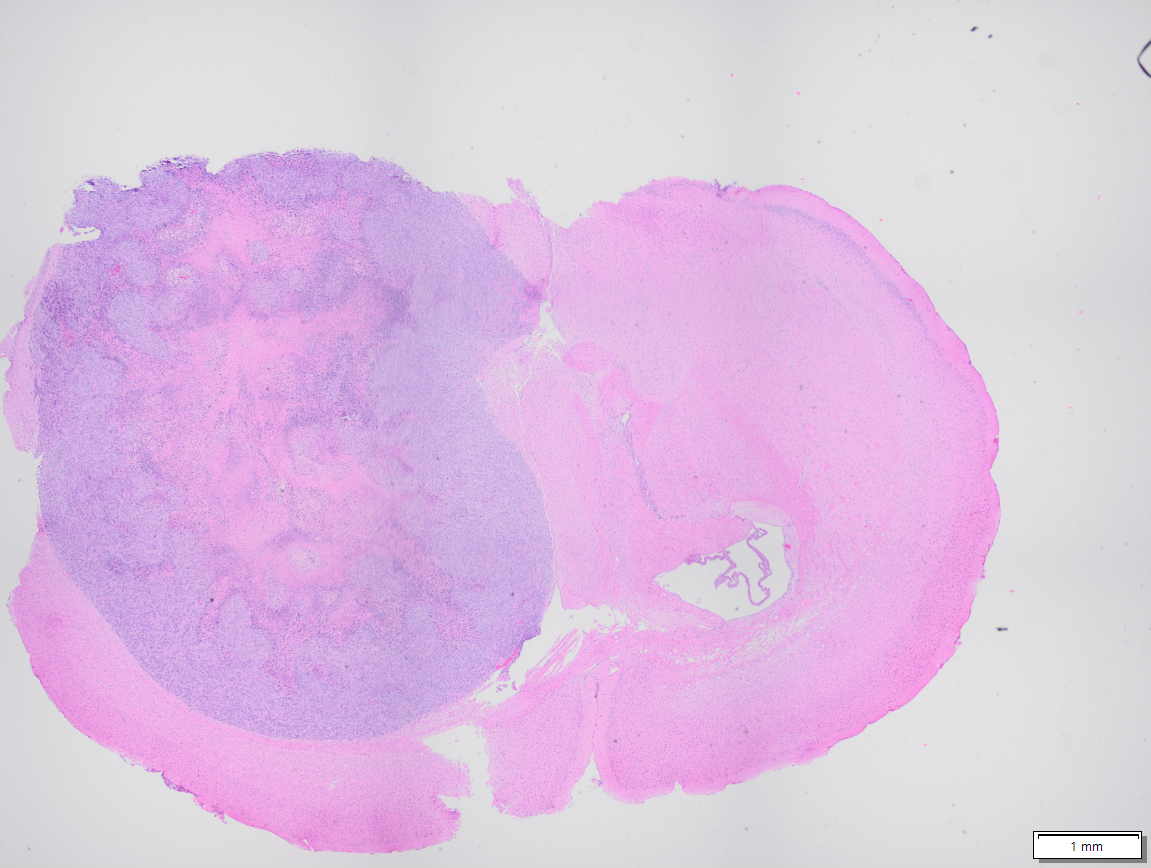

Supplement: Supplementary file 8 — Source data Fig. 7 [file 44318_2024_130_MOESM8_ESM.zip › Figure 7/7H/SC.tif]

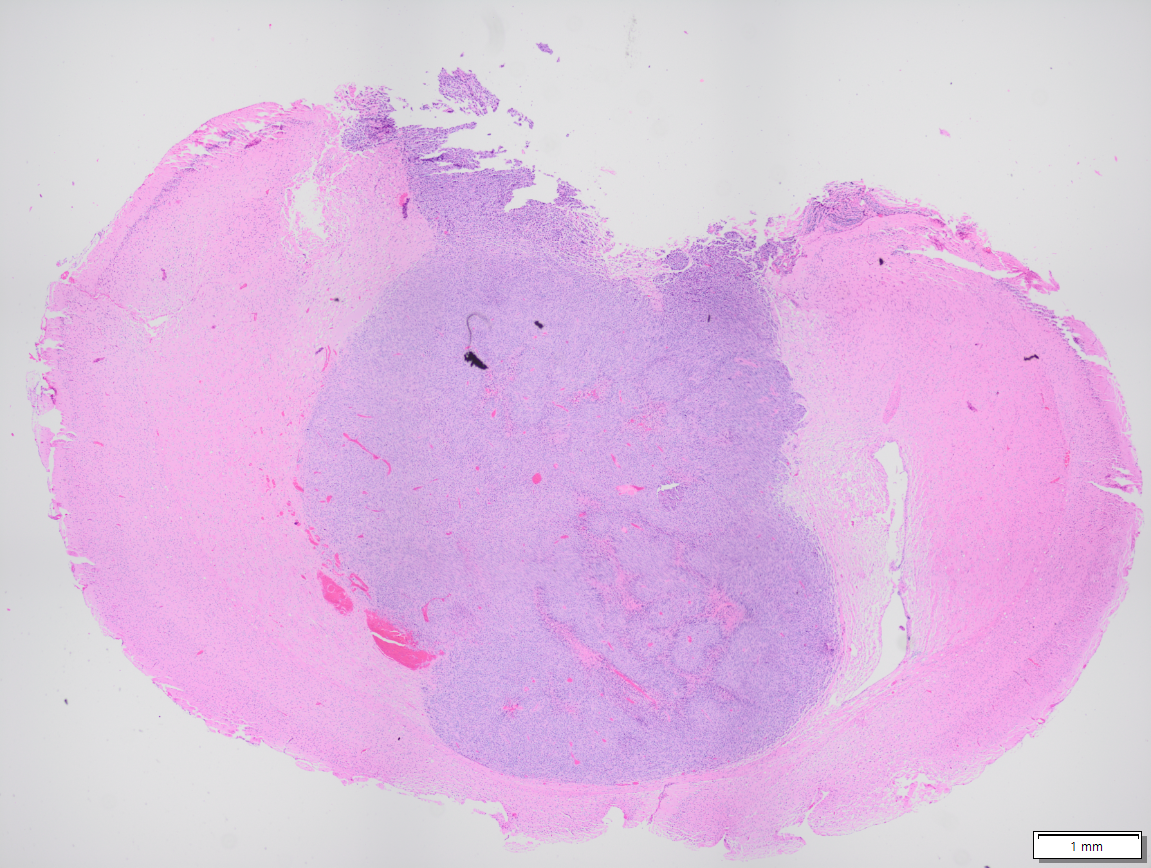

Supplement: Supplementary file 8 — Source data Fig. 7 [file 44318_2024_130_MOESM8_ESM.zip › Figure 7/7H/shVPS34 94.tif]

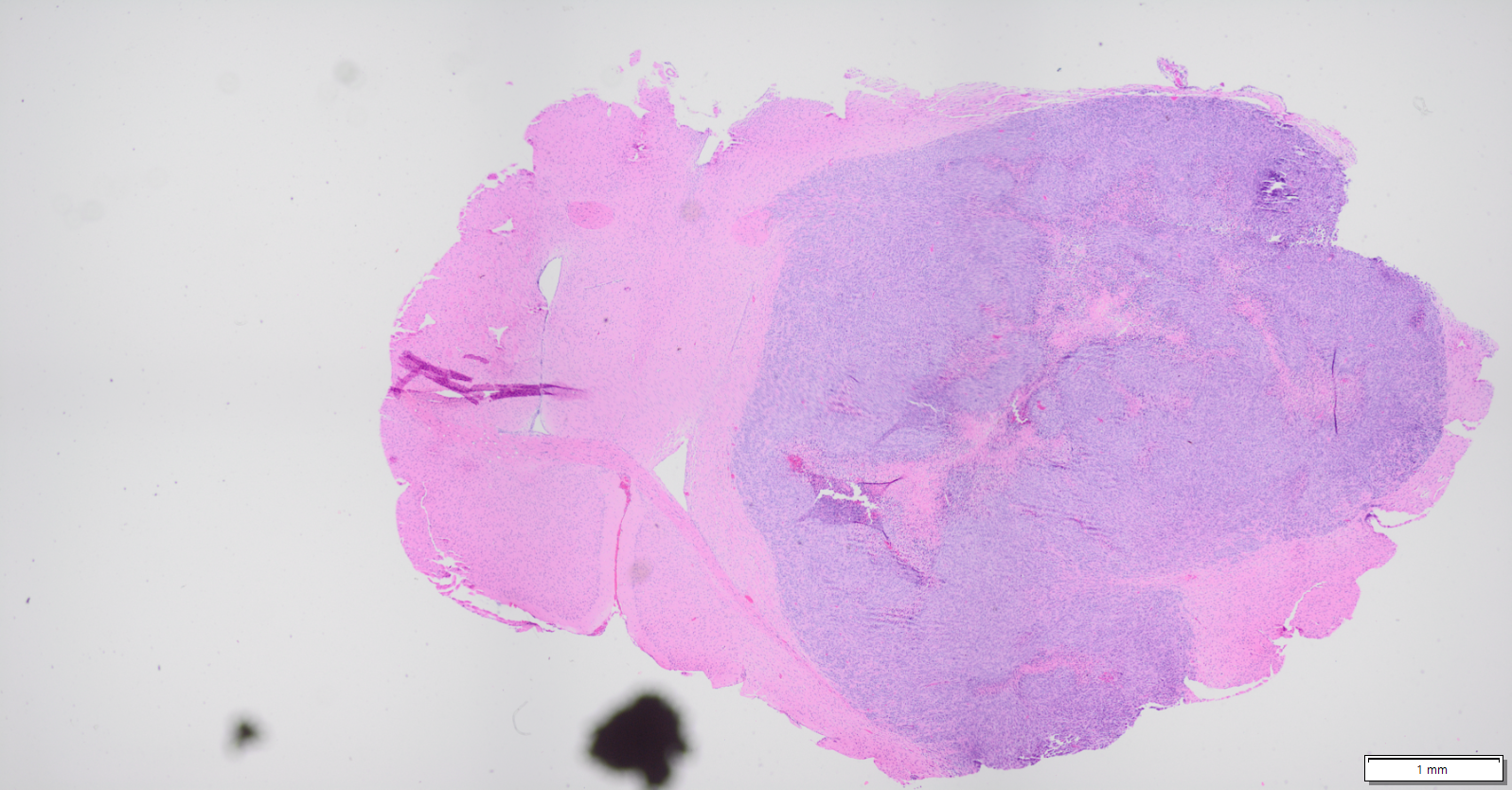

Supplement: Supplementary file 8 — Source data Fig. 7 [file 44318_2024_130_MOESM8_ESM.zip › Figure 7/7H/shVPS34 96.tif]
