# Supplementary material for: LC3-associated phagocytosis of neutrophils triggers tumor ferroptotic cell death in glioblastoma
Source: EMBO J. 2024 May 28;43(13):4. doi: 10.1038/s44318-024-00130-4 (PMC11217441; doi:10.1038/s44318-024-00130-4)
Supplement: Supplementary file 10 — Expanded View Figures [file 44318_2024_130_MOESM10_ESM.pdf]

Expanded View Figures

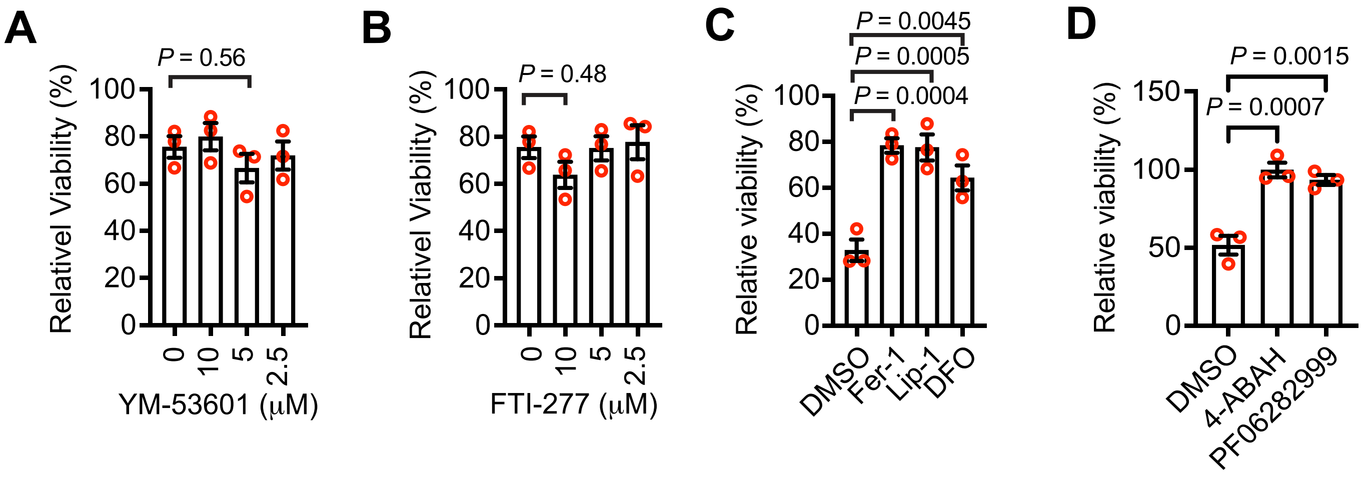

Figure EV1. Viability of LN229$^{TAZ(4SA)}$ cells cocultured with dHL-60 cells in the presence of various inhibitors.

(**A**, **B**) Luciferase assay results for LN229$^{TAZ(4SA)}$ cells cocultured with dHL-60 cells with YM-53691 ($n = 3$ independent experiments), and FTI-277 ($n = 3$ independent experiments). Luminescence readouts of cocultured wells were normalized to their respective LN229$^{TAZ(4SA)}$ cells monocultured wells, both with drugs. One-way ANOVA. (**C**) Luciferase assay results for LN229$^{TAZ(4SA)}$ cells cocultured with dHL-60 cells with ferrostatin-1 (2 μM), liproxstatin-1 (0.2 μM), and DFO (0.2 mM). Luminescence readouts of cocultured wells were normalized to their respective LN229$^{TAZ(4SA)}$ cells monocultured wells, both with drugs ($n = 3$ independent experiments). One-way ANOVA. (**D**) Luciferase assay results for LN229$^{TAZ(4SA)}$ cells cocultured with dHL-60 cells with 4-ABAH (2 μM) or PF06282999 (2 μM). Luminescence readouts of cocultured wells were normalized to their respective LN229$^{TAZ(4SA)}$ cells monocultured wells, both with drugs ($n = 3$ independent experiments). One-way ANOVA. Error bars, s.e.m.

© The Author(s)

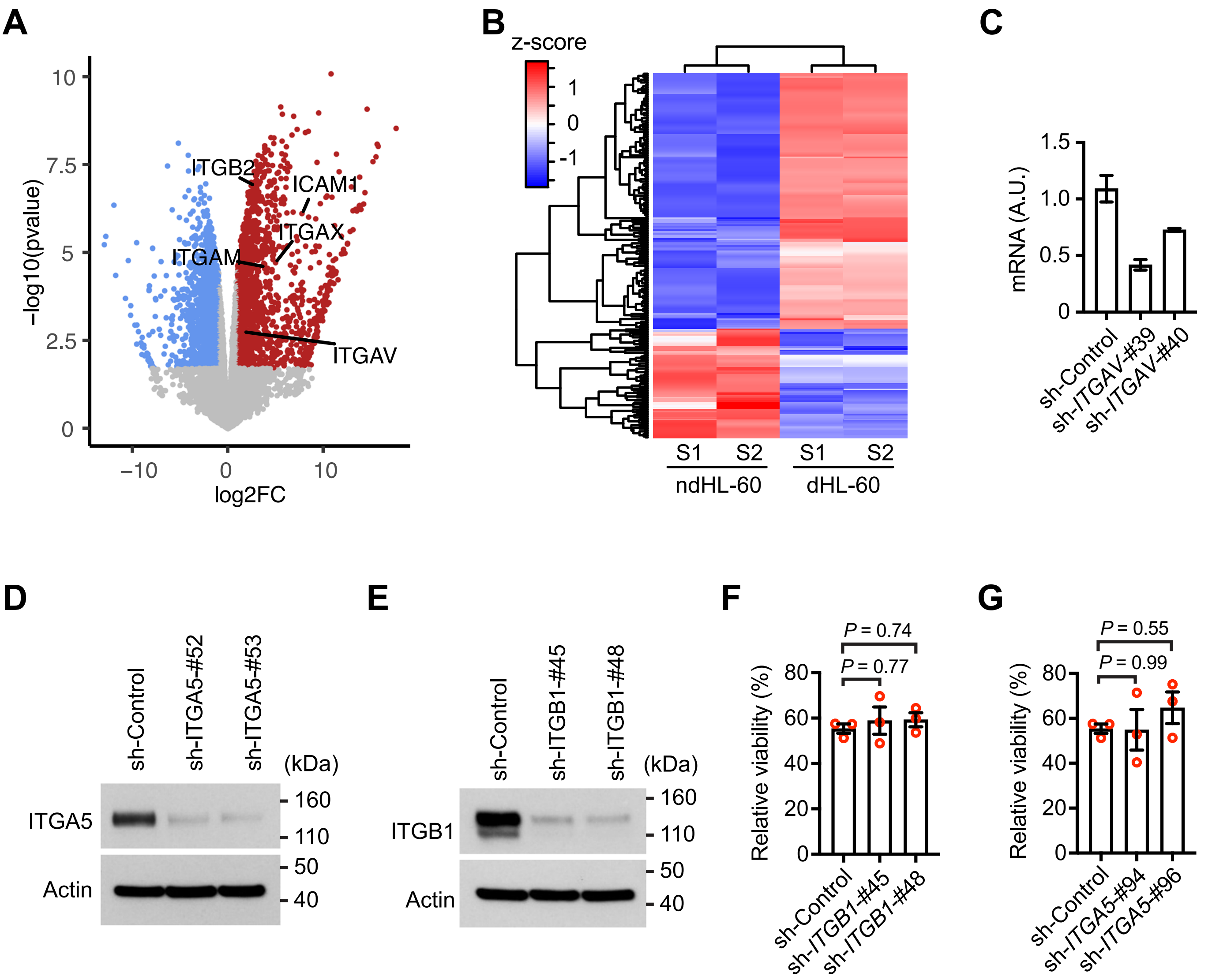

Figure EV2. Integrin expression and function in the coculture of LN229$^{TAZ(4SA)}$ cells and dHL-60 cells.

(**A**) Volcano plot of differentially expressed genes in dHL-60 cells compared to ndHL-60 cells. Upregulated integrin family genes are labeled. The Benjamini-Hochberg False Discovery Rate (FDR) test. (**B**) Heatmap plot of differentially expressed genes related to the neutrophil degranulation pathway. Two replicate samples (S1, S2) were analyzed in each condition. (**C**) dHL-60 cells transduced by indicated shRNAs were subjected to qRT-PCR ($n = 2$ technical replicates). (**D**, **E**) LN229$^{TAZ(4SA)}$ cells transduced by indicated shRNAs were subjected to Western blotting. (**F**, **G**) Cancer cell viability determined using luciferase readouts for LN229$^{TAZ(4SA)}$ cells transduced with indicated shRNAs cocultured with dHL-60 cells. Luminescence readouts of cocultured wells were normalized to their respective monocultured wells for each LN229$^{TAZ(4SA)}$ cell line ($n = 3$ independent experiments). One-way ANOVA. Error bars, s.e.m.

© The Author(s)

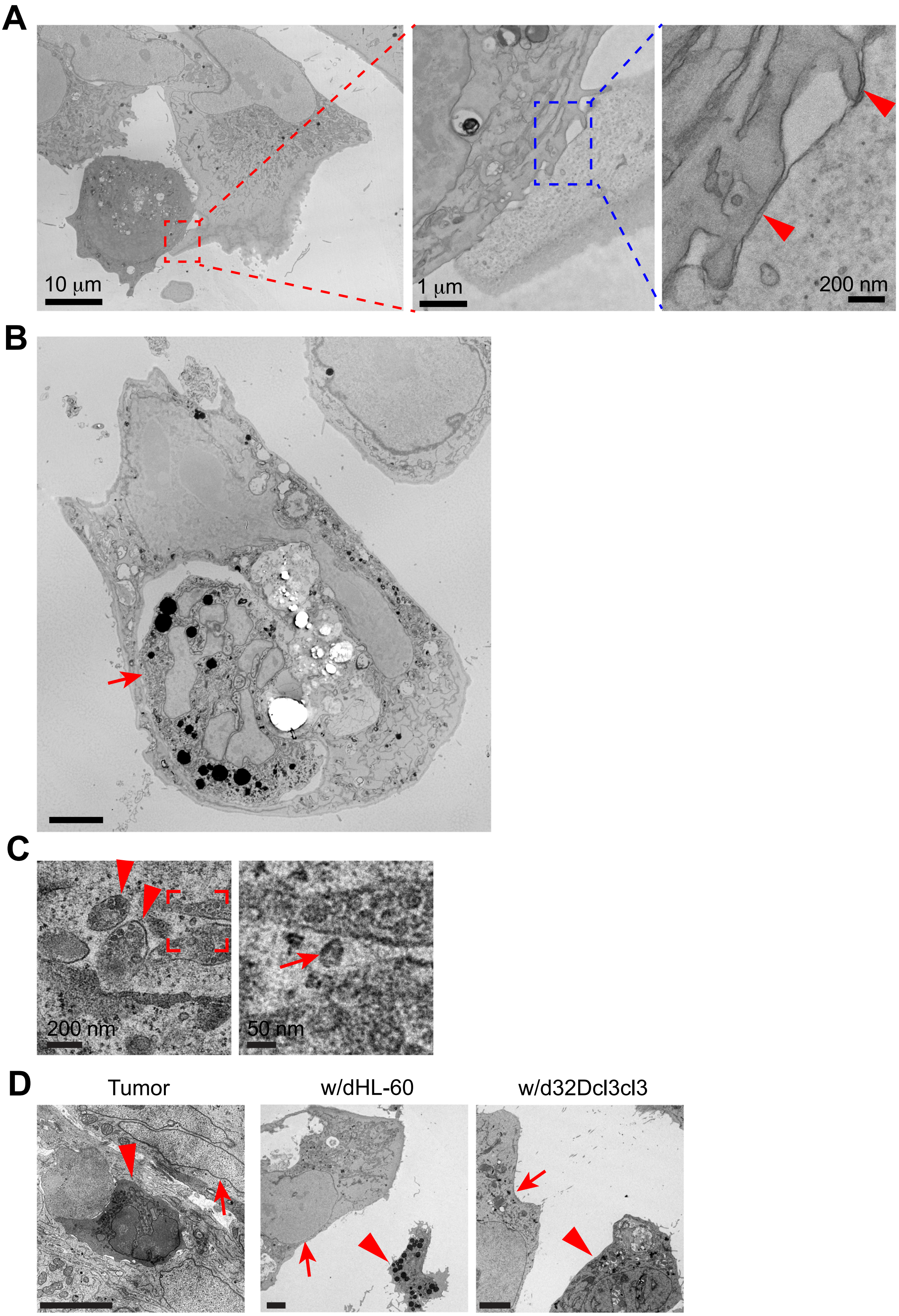
A
10 μm
1 μm
200 nm
B
C
200 nm
50 nm
D
Tumor
w/dHL-60
w/d32Dcl3cl3

Figure EV3. TEM analysis of the coculture of LN229TAZ(4SA) cells and neutrophils.

(**A**) Low- and high-magnification transmission electron microscopy (TEM) images of LN229$^{TAZ(4SA)}$ cells cocultured with dHL-60 neutrophils. The outlined areas are sequentially enlarged and shown as indicated. Arrowheads point adhesion between a tumor cell and a neutrophil. (**B**) TEM images LN229$^{TAZ(4SA)}$ cells cocultured with dHL-60 neutrophils. The arrow points an engulfed neutrophil. Scale bar, 5 μm. (**C**) TEM images of LN229$^{TAZ(4SA)}$ tumor cells cultured with d32Dcl3 neutrophils. The outlined area is shown on the right. Arrowheads point to larger granules. The arrow points to smaller granules. (**D**) TEM images of LN229$^{TAZ(4SA)}$ tumor sections (left), LN229$^{TAZ(4SA)}$ tumor cells cultured with dHL-60 cells (middle), or d32Dcl3cl3 cells (left). Tumor cells (arrows). Neutrophils (arrowheads). Scale bars, 5 μm.

 © The Author(s)

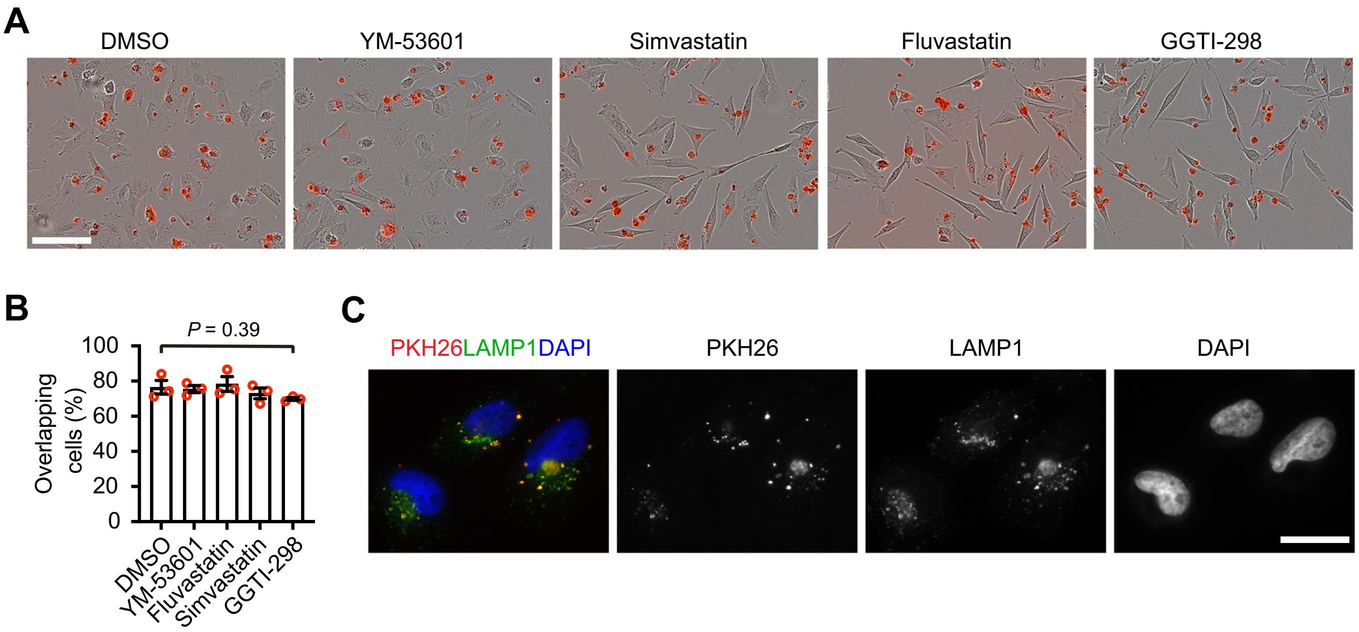

Figure EV4. Internalization of neutrophils by tumor cells.

(**A**) Representative images showing LN229$^{TAZ(4SA)}$ cells cocultured with PKH26-labeled dHL-60 cells with DMSO, simvastatin (2.5 μM), fluvastatin (2.5 μM), GGTI-298 (10 μM), or YM-53601 (10 μM). Scale bar, 100 μm. (**B**) Quantification of LN229$^{TAZ(4SA)}$ cells with overlapping PKH26-labeled dHL-60 cells in the conditions described in (**A**) ($n = 3$ independent experiments). One-way ANOVA. (**C**) Representative immunofluorescent images showing LAMP1 staining, PKH26 signal, and DAPI staining for LN229$^{TAZ(4SA)}$ cells cocultured with PKH26-labeled d32Dcl3cl3 cells ($n = 3$). Scale bar, 20 μm. Error bars, s.e.m.

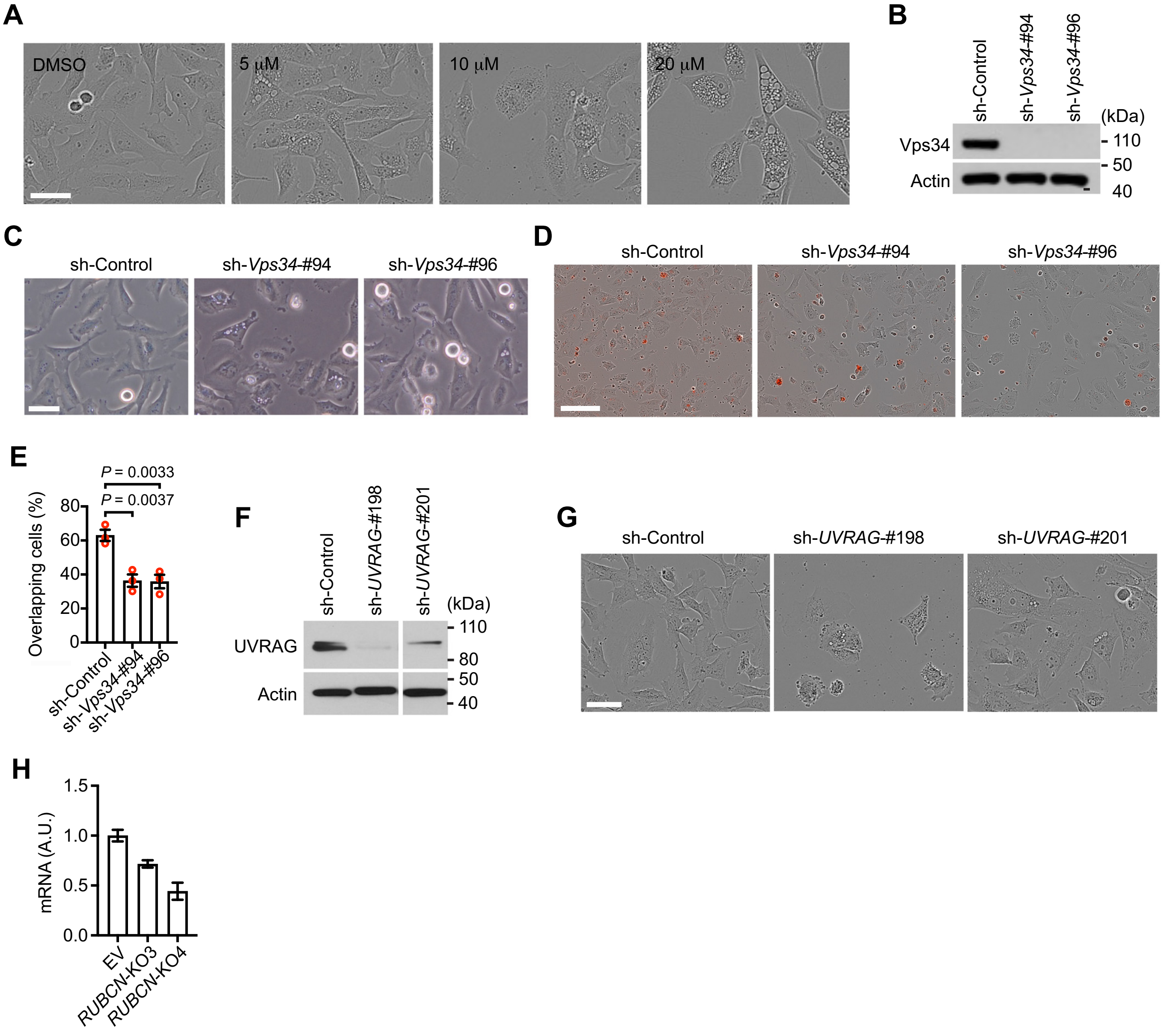

Figure EV5. Characterization of LN229$^{TAZ(4SA)}$ cells upon knocking down various LAP genes.

(**A**) Representative images showing the morphology of LN229$^{TAZ(4SA)}$ cells after 12 h treatment with Vps34-IN1 at listed concentrations. Scale bar, 50 µm. (**B**) LN229$^{TAZ(4SA)}$ cells transduced by indicated shRNAs were subjected to Western blotting. (**C**) Representative images showing the morphology of LN229$^{TAZ(4SA)}$ cells transduced by indicated shRNAs. Scale bar, 50 µm. (**D**) Representative images showing the morphology of LN229$^{TAZ(4SA)}$ cells transduced by indicated shRNAs cocultured with PKH26-labeled dHL-60 cells ($n = 3$). Scale bar, 100 µm. (**E**) Quantification of LN229$^{TAZ(4SA)}$ cells with overlapping PKH26-labeled dHL-60 cells for groups indicated in (**D**) ($n = 3$ independent experiments). One-way ANOVA. (**F**) LN229$^{TAZ(4SA)}$ cells transduced by indicated shRNAs were subjected to Western blotting. (**G**) Representative images showing the morphology of LN229$^{TAZ(4SA)}$ cells transduced by indicated shRNAs. Scale bar, 50 µm. (**H**) LN229$^{TAZ(4SA)}$ cells transduced by indicated gRNAs were subjected to qRT-PCR ($n = 2$ technical replicates). Error bars, s.e.m.

 © The Author(s)
